# HIV serodiscordant sexual partners: social representations of health care professionals

**DOI:** 10.1590/0034-7167-2021-0867

**Published:** 2022-06-24

**Authors:** Valéria Gomes Fernandes da Silva, Isadora Lorena Alves Nogueira, Tatiana Maria Nóbrega Elias, Renata Karina Reis, Nilba Lima de Souza, Rejane Maria Paiva de Menezes

**Affiliations:** I Universidade Federal do Rio Grande do Norte. Natal, Rio Grande do Norte, Brazil.; II Universidade de São Paulo. São Paulo, São Paulo, Brazil.

**Keywords:** HIV, Sexual Partners, Serology, Health Services, Health Professional, VIH, Parejas Sexuales, Serología, Servicios de Salud, Personal de Salud, HIV, Parceiros Sexuais, Sorologia, Serviços de Saúde, Profissional de Saúde

## Abstract

**Objectives:**

to understand the structure of the social representations of health professionals from HIV/AIDS Specialized Care Services about HIV-positive partners.

**Methods:**

this is a qualitative study, based on the structural aspect of Social Representations, developed in specialized services of the metropolitan area of a state in the Northeast Region. Fifty-one professionals were interviewed using the technique of free association of words, processed by the software IRaMuTeQ, by means of prototypical and similarity analysis.

**Results:**

the central nucleus was constituted by the terms “partnership”, “love” and “fear”, showing appreciation of meanings inherent to their beliefs, values and experiences that bring possibilities of reflections for health practices.

**Final Considerations:**

the findings reinforce the impacts generated in the different segments of the lives of people living with HIV and in their emotional bonds. This highlights the need for the implementation of care strategies contemplating the biopsychosocial care model rather than the biological model.

## RESUMO

**Objetivos:**

apreender a estrutura das representações sociais de profissionais de saúde dos Serviços de Assistência Especializada HIV/aids sobre os parceiros que vivem em sorodiferença quanto ao HIV.

**Métodos:**

estudo qualitativo, fundamentado na vertente estrutural das Representações Sociais, desenvolvido em serviços especializados da região metropolitana de um estado da Região Nordeste. Entrevistaram-se 51 profissionais com aplicação da técnica de associação livre de palavras, processadas pelo software IRaMuTeQ, mediante análise prototípica e de similitude.

**Resultados:**

o núcleo central foi constituído pelos termos “parceria”, “amor” e “medo”, evidenciando valorização de sentidos inerentes às suas crenças, valores e experiências que trazem possibilidades de reflexões para as práticas de saúde.

**Considerações Finais:**

os achados reforçam os impactos gerados nos diferentes segmentos da vida das pessoas que vivem com o HIV e em seus vínculos afetivos. Isso ressalta a necessidade da implementação de estratégias assistenciais contemplando o modelo de cuidado biopsicossocial em detrimento do modelo biológico.

## RESUMEN

**Objetivos:**

aprender estructura de representaciones sociales de personal de salud de Servicios de Asistencia Especializada VIH/SIDA sobre parejas que viven en serodiferencia cuanto al VIH.

**Métodos:**

estudio cualitativo, fundamentado en la vertiente estructural de Representaciones Sociales, desarrollado en servicios especializados de la región metropolitana de un estado del Nordeste. Entrevistados 51 profesionales con aplicación de técnica de asociación libre de palabras, procesadas por software IRaMuTeQ, mediante análisis prototípico y de similitud.

**Resultados:**

el núcleo central constituido por los términos “compañerismo”, “amor” y “miedo”, evidenciando valorización de sentidos inherentes a sus creencias, valores y experiencias que traen posibilidades de reflexiones para las prácticas de salud.

**Consideraciones Finales:**

hallados refuerzan los impactos generados en los diferentes segmentos de la vida de las personas que viven con VIH y en sus vínculos afectivos. Eso señala la necesidad de implementación de estrategias asistenciales contemplando el modelo de cuidado biopsicosocial en detrimento del modelo biológico.

## INTRODUCTION

HIV infection is currently treated as a chronic disease, and therefore requires permanent monitoring from the people affected^(1)^. Through the adherence to antiretroviral therapy (ART), the Specialized Care Services (SCS) in Sexually Transmitted Infections STI/HIV/AIDS are faced with the different realities experienced by this public, among which is the experience of affective/sexual relationships of mixed serology in relation to HIV - serodiscordant, as they are also called^(2)^.

The availability of effective therapeutic methods has provided, through adherence to ART, improved morbidity and mortality and quality of life of people living with HIV as well as reduced viral load (VL), which globally reached the concept of undetectable equal to untransmissible (I=I). In this context, serodiscordant partners are present in the routine of SCSs, seeking continuous care and strategies that converge to their needs^(1,2,3)^.

Among the different circumstances surrounding mixed serology are the difficulties partners experience in coping with the serological difference^(4)^. Because it is an infection that has sexual intercourse as the main form of transmission, maintaining safe sex becomes a challenge for prevention and integral health care for couples living in the context of HIV^(5)^.

Besides this, the adoption of secrecy about the HIV status with the partner or family, the deficit of knowledge and understanding regarding the care that is indispensable for this new reality, the stigmas and prejudices perceived in different social spheres, and the family confrontation that is often full of oppression and lack of support configure the scenario that these partners experience^(6)^.

Faced with these dilemmas, partners seek in health services the therapy that the condition of infection requires, as well as the reception and psycho-emotional support necessary to face the pathological condition, which is beyond the biological realm; they try to live a healthy affective relationship, surrounded by care and support, which is often only found in the figure of the health professional^(5)^.

Thus, the role and conduct adopted by health professionals in relation to HIV serodiscordant partnerships are essential to ensure that these individuals feel welcomed and confident to expose their weaknesses, fears, acquired intimacies, that is, that a bond and trust is established between patient and professional^(7)^.

Besides playing a decisive role in the construction of bonds, care and embracement of partners, the health professionals of the SCSs are committed to disseminating knowledge to other professionals in the service network, such as those in Primary Health Care (PHC), and to society, in order to favor the deconstruction of ingrained prejudices involving serodiscordant relationships with regard to HIV^(8)^.

From this perspective, the need to know the perception of health professionals of the SCSs about aspects related to people living with HIV serodiscordance is highlighted. This is important because these professionals are responsible for providing care in its broadest dimension, going through the affective, emotional, family and social follow-up, under the perspective that the thought built by a subject about a phenomenon comes from different senses, meanings, knowledge and experiences^(4,9-10)^.

Thus, this study is based on the following question: What are the social representations of professionals from Specialized STI/HIV/ AIDS Care Services about partners living with HIV serodiscordance?

Considering the theoretical framework of Social Representations (SR), the relationship between the context in which health professionals are inserted and their conduct pattern is highlighted.

## OBJECTIVES

To understand the structure of the social representations of health professionals working in Specialized Care Services for STI/ HIV/AIDS about HIV serodiscordant partners.

## METHODS

### Ethical aspects

The data presented consists of a cut of a master’s thesis developed within the Postgraduate Program in Nursing of the Federal University of Rio Grande do Norte (UFRN), approved by the Research Ethics Committee (REC). The participants consented to participate by signing the Free and Informed Consent Term (FICT); and were identified by the letter P followed by numbers according to the order of the interviews (P1, P2, P3...), to preserve their anonymity.

### Theoretical and methodological framework and study design

This is a qualitative research, based on the theoretical and methodological framework of the Social Representations Theory (SRT ), under the structural aspect of Jean Claude Abric^(11)^. To maintain the methodological rigor of the study, the Consolidated criteria for reporting qualitative research (COREQ) was used as a supporting tool^(12)^.

### Study Scenario

The study was developed in three STI/HIV/AIDS SCS units out of the 14 available in the state of Rio Grande do Norte: two managed by the municipality and one by the state. The choice of these three services is justified by the fact that they are the largest units in terms of population coverage; and because they are located in the metropolitan area of Natal, the state capital. They provide care and follow-up to people living with HIV, within the National Program of STI/HIV/AIDS.

Each service had a minimum multi-professional care team, which works directly with the users and consists of a physician, a social worker, a psychologist, a pharmacist, a nurse, and a nursing technician. The largest unit had 31 caregivers; the second, 13; and the smallest, nine professionals. In addition, the three services had a local coordinator and a coordinator responsible for the program at the municipal and state level.

### Study participants

The sample included 51 professionals from the multi-professional teams of the three specialized services. Inclusion criteria were defined as: being a professional of the basic multi-professional health team of the SCSs, composed of physicians (infectologist or general practitioner, gynecologists), social workers, psychologists, pharmacists and nursing staff (nurses and nursing technicians); professionals of the local management and coordination of the SCS and the STI/HIV/AIDS program at municipal and state levels. Professionals who were absent from the service on vacation, on leave or on medical certificate, retired, those who did not answer three contact attempts, and those who worked in more than one of the SCS units selected in the study and had already been included in the data collection were excluded.

### Data collection and organization

Initially, there was an exploratory observation and familiarization with the environment for a month before the collection, for the subsequent capture of participants. Data was collected between the months of October and December 2020, through interviews scheduled with the local coordination according to the professionals’ schedules and availability of service activities; then, we proceeded to the scheduling with the professionals of the care team. The research guidelines and information were provided to each person contacted, who was presented with the Free and Informed Consent Term (FICT), to request their signature.

For the interview stage, a form-type questionnaire was used, with semi-structured questions consisting of personal and professional information, such as gender, age, time working in the service, training and/or post-graduation in the area of STI/HIV/AIDS. In the second part, open questions were included about the phenomenon of the structure of social representations, by applying the Free Association Test of Words or Expressions (*TALP*). The instrument was tested in order to validate the understanding of its content.

The application stage of *TALP* consists in provoking the evocation of words by means of one or more inductive stimuli^(13-14)^. Thus, after filling in the socio-demographic data, the participant was asked to mention five words that came to mind from the inductive term “people living in serodiscordance regarding HIV” and justify the choice of each term with short sentences. The inductive term chosen was considered the expression closest to the language of professionals and free with regard to the possibilities of inducing responses.

The interviews were conducted individually and ensured the privacy of the interviewee in an environment without noise or interruptions from third parties. The duration of the interviews varied between 14 and 58 minutes; and, to ensure anonymity, the identification of the participants was by means of a code: the letter “P” was used to refer to the word “professional”, followed by a cardinal number corresponding to the order of the interviews (P1, P2, P3...).

It is worth noting that the data collection phase took place during the pandemic of COVID-19, therefore the safety protocols instituted by the services were strictly adopted, such as: minimum distance of 1.5 m between the researcher and the participant, rooms without the use of air conditioning, with open doors and windows, continuous use of personal protective mask in order to ensure the protection of the researcher in the field and of the participants.

### Data analysis

In the first stage of the analysis, all evocations were typed in Microsoft Word program, in the manner and order in which they were evoked. Afterwards, they went through the lemmatization process (reduction of the words to the same radical) and categorization by the researcher (grouping of the words that are similar in their senses and meanings), aiming to avoid ambiguities and divergences. After this step, the evocations were transcribed into a spreadsheet of Libre Office software and then processed in the software *Interface de R pour les Analyses Multidimensionelles de Textes et de Questionnaires* (IRaMuTeQ), version 7 alpha 2.

This was followed by the prototypical analysis, based on the evaluation of the saliency of the representational data by means of two items that were defined as criteria: “frequency” and “average order of evocation (AOE)”. In frequency, evocations with AOE ≥ 3 (defined by the researcher) were processed; those evocations with AOE ≤ 2 were considered low; and AOE ≥ 3, high^(14-15)^. Thus, it was possible to obtain the table of four houses, divided into four quadrants. This consists of a central core of the most salient social representations (SRs), commonly evidenced in the upper left quadrant; and the others contain peripheral elements that carry more particular aspects, which, in the totality of their composition, shape the structure of the SRs^(11)^.

The similarity analysis identified the set of semantic categories by a correlation and co-occurrence analysis of the evocations, in order to identify the neighborhood relationship between the terms, the connotation assumed by them, and to confirm or question the centrality hypothesis resulting from the four-case framework^(16-17)^.

The interpretation of the data was based on the theoretical assumptions of the structural approach of SRT^(11)^. The justifications attributed to the terms considered most important were transcribed in full and used to substantiate the terms that make up the four boxes, helping in the understanding of the meanings given to the terms evoked.

## RESULTS

Initially, the characterization of those involved in the study will be presented, followed by the four boxes, justifications for the evocations and similarity analysis.

The 51 participants were mostly female (42), with a predominant age range of 41 to 54 years (23). The predominant level of education was higher education (40), followed by technical education (11), and a lower number of participants reported having specialization or specific training to work in the service (31). As for the professionals’ performance, a large part of them were part of the multi-professional assistance team (45), including doctors (15), nursing technicians (11), pharmacists (7), nurses (6), social workers (3) and psychologists (3). The time of professionals working in the SCSs was in the interval of nine days to 37 years, being most of them in the interval of nine days to three years (26).

In the prototypical analysis, the inductive term “people living with HIV serodiscordance” resulted in 255 evocations and 93 distinct evocations. After treating these words through the lemmatization and categorization process, the number of different words evoked by the professionals was 47, of which 23 (48.93%) were used for processing in the software after excluding the evocations with frequency below three. Thus, after the combined analysis of the “frequency” and “AOE” axes, we obtained the fourbox table illustrated in Chart 1, with a frequency of 9.96 and an AOE of 3.06 on a scale of 1 to 5.

**Chart 1:** Four-box chart related to the inductive term “people living with HIV serodiscordance”, Natal, Rio Grande do Norte, Brazil, 2021

**f ≥ 9.96**	**AOE ≤ 3.06**			**AOE > 3.06**		
	**CENTRAL NUCLEUS (QSE)**			**PERIPHERY NUCLEUS (QSD)**		
		***f***	**AOE**		***f***	**AOE**
	Partnership	31	2.9	Prevention	27	3.4
	Love	19	2.6	Treatment	12	4.3
	Fear	17	2.8	Lack of knowledge	11	3.4
				Care	11	3.3
***f* < 9.96**	**CONTRAST ZONE (QIE)**			**SECOND PERIPHERY (QID)**		
		***f***	**AOE**		***f***	**AOE**
	Acceptance	9	2.7	Dificulties	8	3.4
	Embracing	8	2.4	Responsibility	7	3.4
	Risk	8	1.9	Comprehension	7	3.4
	Sexual behaviour	7	2.6	Life	6	3.8
	Prejudice	7	2.9	Courage	6	3.2
	Negative	6	2.7	Secrecy	5	3.8
	Respect	6	2.7	Loyalty	3	3.3
	Doubts	5	2.8			
	Family	3	2.7			

f – frequency; AOE – average order of evocations.

Source: Data processed in IRaMuTeQ software.

The intersection of the coordinates “frequency” and “AOE” indicated the semantic elements “partnership”, “love” and “fear” in the upper left quadrant as likely representations of the central core, by a high number of participants, with more immediate evocations.

*Love is a feeling that imposes no conditions. You love the other just the way he or she is. When a person loves, he or she does not impose conditions. Love comes naturally.* (P47)

*One has to be the other’s partner, to trust the other; one has to be very companion, partner, and friend, to accept this disease.* (P8)

*There is the fear of losing the person next to you, fear of seeing the person suffer, fear of acquiring HIV.* (P12)

*Will I be able to live happily? Have children? Have medicines? These insecurities lead to fear because it is something much stigmatized.* (P40)

The upper right quadrant had its probable composition centered on the evocations “prevention”, “treatment”, “ignorance” and “care”. These terms, therefore, represent the first periphery of the frame and are configured as probable secondary elements to the central nucleus for presenting high frequency and high AOE.

*When their partner has a compromised health condition, they have this care. One brings care to both of them, and we see this involvement of the negative partner with the partner that is positive in the services.* (P36)

*Being able to know what it is, and how it is transmitted, the difficulties, the forms of protection like PrEP. Not to remain just in what I was told, and not be a hostage of this, but to seek knowledge. There are couples here who are serodiscordant, have a negative child, I see that they sought through knowledge this happy “ending”.* (P29)

In the third quadrant (lower right quadrant), considered a near periphery or second periphery, the probable terms consisted of “difficulties”, “responsibility”, “understanding”, “life”, “courage”, “secrecy” and “loyalty”. These terms represent more particular aspects of the cognitive constructions of the professionals, since a small number of them mentioned them.

*The person who is serodiscordant doesn’t accept it himself, he hides it, sometimes he is not sincere to tell his partner about his HIV status. He/she is going to have a relationship with another, so he/she has to be sincere.* (P25)

*The understanding is to know, within your educational, social and cultural possibilities, your risks when faced with the choice of being together.* (P38)

*A mutual responsibility, both for the carrier patient to take treatment and reach an undetectable viral load, and for the HIVnegative patient to seek for example PrEP, in order to maintain a safe relationship.* (P47)

*Whether you like it or not, the use of condoms is seen as a limitation in sexual intercourse.* (P39)

In the last quadrant (lower left quadrant), called “contrast zone”, are the clearly peripheral elements that had low frequency, but were considered important because they were readily spoken evocations. The probable composition was with the terms “acceptance”, “welcome”, “risk”, “sexual behavior”, “prejudice”, “negative”, “respect”, “doubts” and “family”.

*We assist many couples like this, we do sensitive and qualified listening. They arrive very sensitive in relation to prejudice and discrimination. It is important to place yourself to welcome their anguish, doubts, and insecurities.* (P5)

*Their doubts have to do with knowing the risks of transmission when they are in this type of relationship.* (P35)

*When I think about sex, I imagine that people who are serodiscordant, they look to the service for the best way to have sex.* (P13)

*I think it is possible for a couple that is serodiscordant to start a family, as a couple, with children, independently.* (P16)

**Figure 1:**
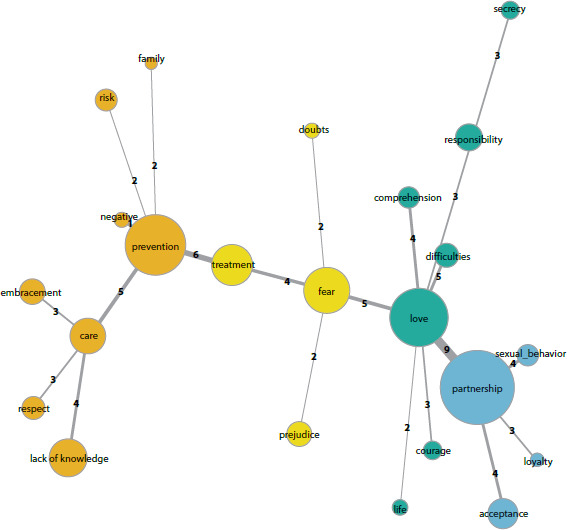
Dendrogram of Similarity

Source: Similarity analysis data processed by IRaMuTeQ software.

Figure 1 below represents the similarity dendrogram from the similarity analysis that reveals the co-occurrences among the words and highlights the indications of connectedness among the evocations. The edges represent the strength of the connection between the association values of the words; and the vertices (circles), the formation of semantic nuclei of meaning, being proportional to the frequency of the evoked words. The colors illustrate the formation of communities, in which the approximation of terms that have a greater relationship with each other is demonstrated.

Its structure shows that the major organizing axes of the representations are revealed by the semantic nuclei “partnership”, “prevention”, “love” and “fear”. This confirmed the centrality of the central zone of the four-house framework, associated with the presence of the peripheral elements that complement and relate to the meaning of each core.

## DISCUSSION

The framework of four houses brings, in its central core, the probable identity and constancy of the group, according to the collective memory and the consensual character of the subjects about an object in the context of social representations^(11)^. The central core hypothesis is seen as the determination of a partnership between serodiscordant people; a union governed by the sentimental dimension of love, understood as something that overcomes everything and becomes the starting point for the possibilities of existence of such relationships.

The literature corroborates these meanings and reveals that the sentimental aspects are configured as an important link between the partners and that the professional should value such aspects during the therapeutic follow-up, in view of the interferences that affective feelings may generate in the health-disease process^(18)^. It is observed that the centrality of this representation reinforces perspectives indicating that the involvement with sex, despite its relevance, gives way to the protagonism of feelings, such as love, care, and even coexistence with the partner. This relationship could be confirmed in the similarity analysis, in which it is possible to notice the strength of the edge relating the terms “partnership” and “love”.

Even in the face of the sense of love, as something that overcomes everything, these people are not exempt from feeling fear of the possible contamination of the partner or of not being accepted and even of not being able to have children. These fears are sharpened, especially in the figure of the HIV-negative partner^(19)^.

On the other hand, the literature also reveals that fear and, many times, prejudice are realities that are being overcome, because having a partner with different serology is associated with positive indicators, with influence on treatment adherence, better social support, which contributes to improved quality of life^(20)^. Scientific advances, particularly in recent years, show that people living with HIV and with an undetectable viral load do not transmit HIV to their sexual partners. Such advances completely change the perspective for HIV prevention among serodiscordant partners^(21-22)^.

The meanings presented in the second quadrant strengthen and give sustainability to the beliefs found in the central core due to the high frequency^(11)^. Thus, if love in these partnerships prevails, then prevention becomes a consequence in order to protect the HIV-negative partner from HIV contamination and minimize fears to live a safer affective and sexual relationship and with quality of life, a hypothesis confirmed in the centrality and connection of these elements in the similarity analysis^(23)^.

Another representation accessed from this second quadrant, likely to bring meaning to the element “fear” of the central nucleus, is the lack of knowledge. This is because, for the professionals, when it comes to HIV infection, partners are not always fully aware of the forms of transmission and prevention, so that such ignorance leads to daily living with fear^(18)^. This possible representation is ratified by the similarity analysis (Figure 1), when we see the connection relation of two communities, represented by the central nucleus “prevention” and “treatment”; and in its peripheries, connections with the terms “negative”, “care”, “ignorance” and “fear”, respectively.

The third quadrant reveals probable representations considered less important for health professionals, given the low frequency of elements and the lack of readiness to evoke them. Even being considered by professionals as courageous people for living a relationship of this nature, the representations also **reveal themselves: in the imbricated difficulties of sexuality**, experienced by partners and seen as feelings of guilt; in the lack of knowledge; and in the resistance to the use of preventive methods, such as condoms, requiring from partners more involvement, knowledge and awareness about the risks of an unprotected sexual relationship.

The study by Boa et al.^(24)^ (2018), on the social representations of women living with HIV, highlights the difficulty to experience sexuality between partners, more precisely the sexual act. This circumstance is seen as regulated and endowed with normative prescriptions, imposed by health professionals, so that sexual intercourse comes to be seen in a limited and not very pleasurable way.

A broad approach to HIV prevention methods is pointed out as a fundamental strategy by health professionals. In it, the use of condoms should not be the only method presented to partners, within the diversity that combined prevention makes possible, because each partnership must be welcomed and evaluated according to their singularities and difficulties^(10)^.

It is worth saying that treatment as prevention to reach an undetectable viral load is a scientifically proven effective method that brings positive implications for the sexual health of HIV serodiscordant couples^(21-22)^.

Another meaning that emerged was the responsibility with the partner’s health, as well as loyalty, considered indispensable in this relationship so that there is no secrecy, especially regarding positive serology, a crucial factor when taking into account the quality of life of both partners. This relationship can be visualized in the similarity dendrogram.

As for the contrast zone quadrant, the elements present show a complementary relationship with the first periphery - second quadrant^(11)^ — by bringing meanings that refer to the coexistence between partners, such as the acceptance of the HIV-negative partner and the necessary respect that the relationship requires, an aspect evidenced in the professionals’ reports.

In turn, the element “risk”, even presenting a low frequency, was the most readily evoked term in the entire table, which reveals its importance in the representations of health professionals, when facing the serodiscordant relationship as a risky relationship. This occurs not only because of the possibility of contamination of the HIV-negative partner, but because it is a relationship with risks of suffering, evidenced by the possibility of ending the relationship or losing the partner.

Also, a sense to be considered is the prejudice in the family, work, and circle of friends and even among partners. Perhaps this situation happens because of the lack of knowledge about HIV, associating it with the term “fear”, as can be seen in the similarity analysis, since partners may feel fear of social exclusion^(6)^.

Finally, still in the contrast zone, there is a need for an articulation of reproductive planning policies in this context, which is pointed out in the literature as a way to supply this demand for information that is still apparently distant from these users^(1,7)^.

In addition, there is the barrier of fear and shame for seeking the service with the aim of resolving doubts about the desire to have children. It is a reality for partners, arising from the fear of receiving judgments and of being considered undeserving of conception^(10)^.

The embracement between serodiscordant partners is a strategy that should always be prioritized, due to the need for consensus and conceptual understanding about HIV, especially for the seronegative partner, who usually has more doubts about the risks of contamination^(5)^.

Similarity analysis indicates that the terms in the contrast zone of the four-house framework appear on the periphery and relate to the central nuclei of prevention, partnership, fear, and care, which contain characteristics that give meaning to the elements representing the central zone.

### Study limitations

The study had limitations regarding the comprehensiveness of the study scenarios, as only professionals working in the metropolitan area of Natal were approached, making it impossible to generalize the results. Moreover, the social isolation caused by the pandemic of COVID-19 made it impossible to access some professionals who were away from their activities.

### Contributions to the Area

As a contribution, the results of this study indicate aspects of the biopsychosocial context, experienced by partners that can guide and direct the attention of other health professionals, including nurses, so that they can provide humanized and embracing care. In this sense, the present study reinforces the importance of updating knowledge because it deals with a phenomenon that has significant social impact and is inserted in a context in which policies and guidelines constantly evolve.

In addition, nursing plays an essential role in health education activities, both in services and in the social sphere. Therefore, understanding the nuances that involve serodiscordance as to HIV potentializes, in addition to the acquisition of knowledge, the dissemination of information in order to have increasingly aware partners.

It is worth highlighting the innovative character of this production, for dealing with a sensitive topic that covers HIV infection in serodiscordant sexual partners and that can awaken initiatives, from professionals and political decision-makers, for the restructuring or implementation of projects and specific conducts aimed at this public.

## FINAL CONSIDERATIONS

The study allowed us to understand that the structure of the social representations of professionals from Specialized Care Services for STI/HIV/AIDS about partners living with HIV serodiscordance has its centrality linked to the meaning of the partnership that serodiscordant individuals are willing to establish. Such partnership is guided by the feeling of love, seen as an essential factor in the relationship, which makes them able to face the fears inherent to the infection condition.

In addition to enabling the approach of the theme for health professionals from different areas of the network of services that provide assistance to people living with the virus, this study shows that the representations accessed reinforce how HIV infection impacts all aspects of the lives of these people and their emotional bonds. This highlights the need for the implementation of care strategies that contemplate the biopsychosocial care model rather than conducts centered on the biological care model.

It is reiterated the relevance of further studies in order to confirm the centrality of the elements of the central nucleus and to expand the study population beyond the professionals in specialized services. This is because we understand the importance of accessing representations of the phenomenon in question for settings that also deal with these demands, such as those where PHC professionals work.

## SUPPLEMENTARY MATERIAL

Manuscript resulting from Dissertation. Silva, VGF. Social representations of HIV serodiscordant people by health professionals in Specialized Care Services [Internet]. 2021. Federal University of Rio Grande do Norte. Available at: https://repositorio.ufrn.br/handle/123456789/32397.

Silva VGF, Silva CJA, Cassiano AN, Silveira BRD, Carvalho EA, Menezes RMP. Social representations of HIV/AIDS serodiscordant by health professionals: a study study [previous notice]. Online Braz J Nurs. 2020;19. Available from: https://doi.org/10.17665/1676-4285.20206427.

0034-7167-reben-75-06-e20210867-sup01

0034-7167-reben-75-06-e20210867-sup02
