# Supplementary material for: HIV serodiscordant sexual partners: social representations of health care professionals
Source: Rev Bras Enferm. 2022 Jun 24;75(6):e20210867. doi: 10.1590/0034-7167-2021-0867 (PMC9728823; doi:10.1590/0034-7167-2021-0867)
Supplement: 0034-7167-reben-75-06-e20210867-sup01 [file 0034-7167-reben-75-06-e20210867-sup01.pdf]

UNIVERSIDADE FEDERAL DO RIO GRANDE DO NORTE
CENTRO DE CIÊNCIAS DA SAUDE
DEPARTAMENTO DE ENFERMAGEM
PROGRAMA DE PÓS-GRADUAÇÃO EM ENFERMAGEM
MESTRADO ACADÊMICO

VALÉRIA GOMES FERNANDES DA SILVA

REPRESENTAÇÕES SOCIAIS DAS PESSOAS EM SORODIFERENÇA AO HIV POR PROFISSIONAIS DE SAÚDE NOS SERVIÇOS DE ASSISTÊNCIA ESPECIALIZADA

NATAL-RN
2021

VALÉRIA GOMES FERNANDES DA SILVA

REPRESENTAÇÕES SOCIAIS DAS PESSOAS EM SORODIFERENÇA AO HIV POR PROFISSIONAIS DE SAÚDE NOS SERVIÇOS DE ASSISTÊNCIA ESPECIALIZADA

Dissertação de mestrado apresentada ao Programa de Pós-Graduação em Enfermagem do Centro de Ciências da Saúde da Universidade Federal do Rio Grande do Norte, como requisito parcial para obtenção de Título de Mestre em Enfermagem.

Orientador(a): Profª. Dra. Rejane Maria Paiva de Menezes.

Área de concentração: Enfermagem na Atenção à Saúde.

Linha de pesquisa: Desenvolvimento tecnológico em saúde e enfermagem.

NATAL-RN
2021

Universidade Federal do Rio Grande do Norte - UFRN
Sistema de Bibliotecas - SISBI
Catalogação de Publicação na Fonte. UFRN - Biblioteca Setorial Bertha Cruz Enders - -Escola de Saúde da UFRN - ESUFRN

Silva, Valeria Gomes Fernandes da.
Representações sociais das pessoas em sorodiferença ao HIV por profissionais de saúde nos serviços de assistência especializada / Valeria Gomes Fernandes da Silva. - 2021.
105f.: il.

Dissertação (Mestrado em Enfermagem)-Universidade Federal do Rio Grande do Norte, Cetro de Ciências da Saúde, Programa de Pós-graduação em Enfermagem. Natal, RN, 2021.
Orientadora: Rejane Maria Paiva de Menezes.

1. HIV - Dissertação. 2. Parceiros sexuais - Dissertação. 3. Serviços de saúde - Dissertação. I. Menezes, Rejane Maria Paiva de. II. Título.

RN/UF/BS-Escola de Saúde CDU 616.98:578.828

Elaborado por MAGALI ARAUJO DAMASCENO DE OLIVEIRA - CRB-15/519

VALÉRIA GOMES FERNANDES DA SILVA

REPRESENTAÇÕES SOCIAIS DAS PESSOAS EM SORODIFERENÇA AO HIV POR PROFISSIONAIS DE SAÚDE NOS SERVIÇOS DE ASSISTÊNCIA ESPECIALIZADA

Dissertação apresentada ao Programa de Pós-graduação em Enfermagem, da Universidade Federal do Rio Grande do Norte (UFRN), como requisito parcial para obtenção do título de Mestre em Enfermagem.

Julgamento: APROVADA, em 25 de fevereiro de 2021.

BANCA EXAMINADORA

____

Dra. Rejane Maria Paiva de Menezes
Universidade Federal do Rio Grande do Norte
Orientadora – Presidente da Banca

____

Dr. Francisco Arnoldo Nunes de Miranda
Universidade Federal do Rio Grande do Norte
Membro Interno

____

Dra. Renata Karina Reis
Universidade de São Paulo
Membro Externo à Instituição

____

Dra. Maria Aparecida Alves de Oliveira Serra
Universidade Federal do Maranhão
Membro Externo à Instituição

À minha família, por me motivar e apoiar na realização deste sonho. E a todas às pessoas que experienciam a vivência da sorodiferença para o HIV, por me motivarem a buscar compreensão e entendimento sobre esse fenômeno nos serviços de saúde.

AGRADECIMENTOS

Agradeço a **Deus**, por estar comigo integralmente nessa jornada, e ser luz em minha mente e meus passos em cada decisão e caminho percorrido.

À minha **família**, por me apoiar e compreender em cada etapa, principalmente na decisão de sair de casa em busca do sonho de fazer um mestrado. Aqui menciono meus pais, **Valdirene Fernandes Martins** e **Sinval Gomes da Silva**, minha irmã **Vanessa Fernandes**, minha fonte de fortaleza, apoio e inspiração.

À minha orientadora, **Prof. Dra. Rejane Maria Paiva de Menezes**, por me acolher, me orientar, me ensinar e proporcionar experiencias e aprendizados na vida acadêmica que levarei por toda a vida. Agradeço ainda por toda sua paciência, compreensão e empatia para comigo durante esses dois anos de formação.

À minha **Turma Mestrado 2019**, por caminharem comigo nessa jornada compartilhando alegrias e aprendizados.

Ao meu grupo de amigos **Isabelle da Silva**, **Bruno Neves**, **Renato Sales**, **Ana Carolina Carino** e **Renata Marinho**, por me acolherem em seus corações, pelo laço de amizade construído e fortalecido nas batalhas mais difíceis dessa jornada. Vocês provaram que na Pós-Graduação podemos encontrar amizade verdadeira.

À **Alex Miranda**, por todo companheirismo, incentivo, força e dedicação em me ajudar em todos os momentos dessa trajetória.

À **Ricardo Borges**, um grande amigo que me incentivou e plantou em mim o desejo de ir em busca do mestrado na UFRN.

Aos amigos, **Ana Paula Carino** e **Alexandre Carino**, que me acolheram como família na cidade de Natal, me dando suporte afetivo que foi crucial nos momentos difíceis dessa jornada.

À doutoranda **Alexandra Cassiano** e Pós-doutora **Mônica Lima**, pela ajuda e apoio em minha pesquisa com suas experiências, apoio este que se estendeu às minhas dificuldades pessoais.

Às coordenadoras do Núcleo de IST/Aids e Hepatites Virais da Secretaria Municipal de Saúde de Natal **Emilly Bezerra S. de Miranda,** do Estado do RN **Juliana Campos**

Soares e da Secretaria Municipal de Saúde de Parnamirim, **Izabell Crystinna Lima Alves,** pela permissão e apoio prestado durante todas as fases deste estudo, desde o fornecimento de informações na sua elaboração a implementação e participação.

Às coordenadoras dos Serviços de Assistência Especializada IST/HIV/Aids do município de Parnamirim **Izabell Crystinna Lima Alves**, de Natal **Thaise Cristhine Fernandes Galvão e Figueirêdo** e do Estado do RN **Roberta Torres de Matos Serejo Alves**, pela colaboração e assistência antes e durante a etapa de coleta de dados.

À todos os **profissionais de saúde da equipe multiprofissional dos SAE Natal, SAE Hospital Giselda Trigueiro e SAE Parnamirim**, pela colaboração e empenho em contribuir com minha pesquisa mesmo diante das adversidades causadas pela pandemia da COVID-19.

À minha banca examinadora, **Profa. Dra. Maria Aparecida de Oliveira Serra** responsável por me apresentar o mundo da pesquisa na graduação, parceria esta que se estendeu no mestrado; **Profa. Dra. Renata Karina Reis** na qual tenho profunda admiração por ser referência nacional na temática deste estudo; **Prof. Dr. Francisco Arnoldo Nunes de Miranda**, por me privilegiar com seu vasto conhecimento sobre a Teria das Representações Sociais, e seu acolhimento e generosidade ímpar em nossos momentos. Gratidão por contribuírem com meu trabalho.

À **Coordenação de Aperfeiçoamento de Pessoal de Nível Superior** pelo auxílio por meio da Bolsa de Pós-graduação concedida durante o curso de mestrado.

Ao **Grupo de Estudos e Pesquisas em Enfermagem, Saúde e Envelhecimento (GREPENSE)**, pelo companheirismo na pesquisa e experiencias vivenciadas, que foram de extrema importância nessa trajetória de amadurecimento e aprendizado.

Ao **Programa de Pós-Graduação em Enfermagem da Universidade Federal do Rio Grande do Norte**, juntamente com todo o corpo docente pela dedicação na formação de mestres e doutores em enfermagem.

À minha terapeuta **Clemonice Alves**, que entrou em minha vida no momento oportuno para me ajudar a vencer momentos difíceis durante essa fase final do mestrado.

*À cidade de **Natal-RN**, por me acolher e me proporcionar experiências de vida inesquecíveis.*

RESUMO

No contexto dos serviços de assistência à saúde especializados, constantemente os profissionais se deparam com a realidade das pessoas que convivem com a sorodiferença para o HIV. O presente estudo tem como objetivo analisar as Representações Sociais sob a vertente estrutural das pessoas em sorodiferença para o HIV, elaboradas por profissionais de saúde de Serviços de Assistência Especializada em Infecções Sexualmente Transmissíveis HIV/Aids. Trata-se de estudo descritivo, qualitativo, ancorado nos pressupostos da Teoria das Representações Sociais, na vertente estrutural. O local de realização incluiu, três Serviços de Assistência Especializada em IST/HIV/Aids da região metropolitana do estado do Rio Grande do Norte, e teve a participação de 51 profissionais de saúde, atuantes em equipes multiprofissionais assistenciais, além dos profissionais coordenadores dos respectivos serviços e programas de saúde. A coleta de dados ocorreu no período de outubro a dezembro de 2020, e foi mediada pelo uso da Técnica de Associação Livre de Palavras associada ao modelo de substituição e descontextualização (zona muda), com o termo indutor “Pessoas em sorodiferença para o HIV”. Os dados foram analisados através do processamento prototípico (quadro de quatro casas) de cada matriz, para as quais calcularam-se, automaticamente, as frequências intermediárias (±9.96 matriz 1 e ±9,95 matriz 2) e as Ordens Médias de Evocações (matriz 1: ±3,06; matriz 2: ±2,95), e a análise de similitude, afim de verificar as composições e co-ocorrência das palavras. O processamento analítico se deu no software Interface de *R pour les Analyses Multidimensionnelles de Textes et de Questionnaires* (IRAMUTEQ), versão 7 alpha 2. A pesquisa foi aprovada pelo Comitê de Ética em Pesquisa da Universidade Federal do Rio Grande do Norte, 30794020.6.0000.5537. Participaram 51 profissionais, a maioria do sexo feminino (82,35%) e com faixa etária de 41 anos a 54 anos (45,10%). Quanto ao nível de escolaridade, 78,43% possuíam ensino superior e 21,57% ensino técnico, 41,18% relataram possuir especialização ou capacitação específica para atuar no serviço. Do total de participantes, 11,76% eram coordenadores dos serviços e programas IST/HIV/Aids, e 88,24% profissionais da equipe assistencial multiprofissional, destes, sendo 33,33% médicos, 24,44% técnicos de enfermagem, 15,55% farmacêuticos, 13,33% enfermeiros, 6,66% assistente social e 6,66% psicólogos. Quanto ao tempo de formação dos profissionais, houve variação de 1 ano e 10 meses a 46 anos de profissão, assim como o tempo de serviço no SAE esteve no intervalo de 9 dias a 37 anos. Na análise prototípica, verificou-se que o termo indutor referente a matriz 1, resultou em 255 evocações e 93 palavras distintas, enquanto que a matriz 2 (zona de substituição e de descontextualização) teve 255 evocações e 156 palavras distintas. Após os processos de lematização e categorização, o número de evocações diferentes foram de 47 para a matriz 1 e 51 para a matriz 2. Após a exclusão das evocações com frequência inferior a três, resultou-se em um aproveitamento das evocações de 48,93% (23) e 43,13% (22), respectivamente. Na matriz 1, a possível representação das pessoas que vivem em sorodiferença para o HIV, se cristalizou semanticamente no núcleo central, pelos termos: parceria (f=31; OME: 2.9), amor (f=19; OME: 2.6), medo (f=17; OME: 2.8). Na matriz 2, as representações foram objetivadas pelos termos preconceito (f=24; OME:

2.5), desconhecimento (f=21; OME: 2.1), medo (f=16; OME: 2.8), loucura (f=10; OME: 2.4) e amor (f:10; OME: 2.5). Na análise de similitude a matriz 1, os termos que apresentaram forte co-ocorrência e conexidade foram: “parceria”, “amor” e “medo”; e a matriz II, com os termos: “preconceito”, “desconhecimento”, “medo”, “loucura” e “amor”. As Representações Sociais tidas pelos profissionais de saúde acerca da sorodiferença se concentraram nos significados atribuídos estruturalmente ao amor, medo, parceria, assistência, preconceito, desconhecimento e loucura o que denota a valorização de sentidos normativos que trazem possibilidades de reflexões para as práticas de saúde.

Palavras-chave: HIV. Parceiros sexuais. Serviços de saúde. Equipe de Assistência ao Paciente. Psicologia social. Enfermagem.

ABSTRACT

In the context of specialized health care services, professionals are constantly faced with the reality of people living with HIV serodifference. The present study aims to analyze the Social Representations under the structural aspect of people in HIV serodifference, elaborated by health professionals from Specialized Assistance Services in Sexually Transmitted Infections HIV / AIDS. This is a descriptive, qualitative study, anchored on the assumptions of the Theory of Social Representations, in the structural aspect. The place of performance included three Specialized Care Services in STI / HIV / AIDS in the metropolitan region of the state of Rio Grande do Norte, and had the participation of 51 health professionals, working in multidisciplinary care teams, in addition to the coordinating professionals of the respective health services and programs. Data collection took place from October to December 2020, and was mediated by the use of the Free Word Association Technique associated with the substitution and decontextualization model (silent zone), with the inductive term “People in serodifference for HIV”. The data were analyzed through the prototypical processing (table of four houses) for each matrix, for which the intermediate frequencies (± 9.96 matrix 1 and ± 9.95 matrix 2) and the Average Evocation Orders (matrix 1: ± 3.06; matrix 2: ± 2.95), and similarity analysis, in order to verify the compositions and co-occurrence of words. The analytical processing took place using the Interface R software for Multidimensional Analyzes by Textes et de Questionnaires (IRAMUTEQ), version 7 alpha 2. The research was approved by the Research Ethics Committee of the Federal University of Rio Grande do Norte, 30794020.6.0000.5537. The 51 participants were mostly female (82.35%), with a predominant age range from 41 to 54 years old (45.10%). Regarding the level of education, 78.43% had higher education and 21.57% technical education, and 41.18% reported having specialization or specific training to work in the service. Of the total participants, 11.76% were coordinators of the responsible services and programs, and 88.24% were professionals from the multiprofessional care team, of which 33.33% were doctors, 24.44% were nursing technicians, 15.55% were pharmacists, 13.33% nurses, 6.66% social worker and 6.66% psychologists. As for the training time of professionals, there was a variation of 1 year and 10 months to 46 years of profession, as well as the time of service in the SAE was in the range of 9 days to 37 years. In the prototypical analysis, it was found that the term inducer referring to matrix 1, resulted in 255 evocations and 93 distinct words, while matrix 2 (substitution and decontextualization zone) had 255 evocations and 156 distinct words. After the stemming and categorization processes, the number of different evocations was 47 for matrix 1 and 51 for matrix 2. After excluding evocations with a frequency less than three, 48.93 evocations were used. % (23) and 43.13% (22), respectively. In matrix 1, the possible representation of people living in serodifference for HIV, crystallized semantically in the central nucleus, by the terms: partnership (f = 31; OME: 2.9), love (f = 19; OME: 2.6), fear (f = 17; OME: 2.8). In matrix 2, the representations were objectified by the terms prejudice (f = 24; OME: 2.5), ignorance (f = 21; OME: 2.1), fear (f = 16; OME: 2.8), madness (f = 10; OME : 2.4) and love (f: 10; OME: 2.5). In the similitude analysis of matrix 1, the terms that showed strong co-occurrence and connectedness were: “partnership”, “love” and “fear”; and matrix II, with the terms: "prejudice", "ignorance",

"fear", "madness" and "love". The Social Representations held by health professionals about serodifference were concentrated on the meanings attributed structurally to love, fear, partnership, assistance, prejudice, ignorance and madness, which denotes the valorization of normative meanings that bring possibilities for reflections on health practices.

Keywords: HIV. Sexual partners. Health services. Patient Assistance Team. Social Psychology. Nursing.

LISTA DE SIGLAS E ABREVIATURAS

APS – Atenção Primária à Saúde

CAAE - Certificado de Apresentação para Apreciação Ética

CEP – Comitê de Ética em Pesquisa

CNS - Conselho Nacional de Saúde

DAS - Departamento de Atenção à Saúde do Servidor

HGT – Hospital Giselda Trigueiro

HIV – Vírus da Imunodeficiência Humana

IST – Infecção Sexualmente Transmissível

MS – Ministério da Saúde

OME – Ordem Média de Evocações

PEP – Profilaxia Pós-Exposição

PN-DST – Programa Nacional de Doenças Sexualmente Transmissíveis

PREP – Profilaxia Pré-Exposição

RN – Rio Grande do Norte

RS – Representações Sociais

SAE – Serviço de Assistência Especializada

SESAP - Secretaria Estadual de Saúde Pública

SIDA – Síndrome da Imunodeficiência Adquirida

SMS – Secretaria Municipal de Saúde

TALP - Teste de Associação Livre de Palavras

TARV – Terapia Antirretroviral

TCLE – Termo de Consentimento Livre e Esclarecido

TCP – Tratamento Como Prevenção

TNC – Teoria do Núcleo Central

TRS – Teoria das Representações Sociais

UFRN – Universidade Federal do Rio Grande do Norte

UNAIDS - Programa Conjunto das Nações Unidas sobre HIV/Aids

ZM – Zona Muda

LISTA DE FIGURAS

Figura 1 - Análise de similitude das evocações (TALP) 64

Figura 2 - Análise de similitude das evocações (TALP ZM) 65

LISTA DE TABELAS

Tabela 1 - Caracterização dos participantes. Natal/RN, 2021. 56

LISTA DE QUADROS

Quadro 1 - Matriz I: Lematização das evocações TALP elaboradas pelos profissionais de saúde 46

Quadro 2 - Matriz II: Lematização das evocações TALP ZM elaboradas pelos profissionais de saúde..46

Quadro 3 - Matriz I: Categorização das evocações TALP elaboradas pelos profissionais de saúde..46

Quadro 4 - Matriz II: Categorização das evocações TALP ZM elaboradas pelos profissionais de saúde..48

Quadro 5 - Quadro de quatro casas 52

Quadro 6 - Quadro de quatro casas – Matriz I, resultantes do termo indutor: "pessoas que vivem em sorodiferença para o HIV" 58

Quadro 7 - Quadro de quatro casas – Matriz II, resultantes do termo indutor: "pessoas que vivem em sorodiferença para o HIV" 60

SUMÁRIO

1 INTRODUÇÃO 17

2 OBJETIVOS 24

2.1 OBJETIVO GERAL 24

2.2 OBJETIVOS ESPECÍFICOS 24

3 REVISÃO DE LITERATURA 25

3.1 O CONCEITO DA SORODIFERENÇA E AS DIFICULDADES ENFRENTADAS PELOS PARCEIROS 25

3.2 OS SERVIÇOS DE ASSISTÊNCIA ESPECIALIZADA E O ATENDIMENTO AOS USUÁRIOS SORODIFERENTES 27

3.3 A ENFERMAGEM NO ATENDIMENTO AOS USUÁRIOS SORODIFERENTES E OS DESAFIOS VIVENCIADOS NO CONTEXTO DOS PROFISSIONAIS DE SAÚDE 30

4 REFERENCIAL TEÓRICO 35

4.1 O UNIVERSO DA TEORIA DAS REPRESENTAÇÕES SOCIAIS (TRS) 35

4.2 JEAN CLAUDE ABRIC E A TEORIA DO NÚCLEO DO CENTRAL (TNC) 37

5 PERCURSO METODOLÓGICO 40

5.1 TIPO DE ESTUDO 40

5.2 CENÁRIO DO ESTUDO 40

5.3 PARTICIPANTES DA PESQUISA 41

5.4 COLETA DOS DADOS 42

5.5 ANÁLISE DOS DADOS 45

5.5.1 Análise Prototípica - Quadro de Quatro Casas 51

5.5.2 Análise de Similitude 52

5.6 ASPECTOS ÉTICOS E LEGAIS DA PESQUISA 53

6 APRESENTAÇÃO E ANÁLISE DOS RESULTADOS 55

6.1 CARACTERIZAÇÃO DOS PARTICIPANTES 55

6.2 ENTREVISTA SEMIDIRIGIDA 57

6.2.1 Processamento Prototípico – Quadro de quatro casas (Matriz I: Técnica de Associação Livre de Palavras) 57

6.2.2 Processamento Prototípico – Quadro de quatro casas (Matriz II: Modelo de substituição e descontextualização – Zona Muda) 60

6.2.3 Análise de similitude – Matriz I Técnica de Associação Livre de Palavras 63

6.2.4 Análise de similitude – Matriz II Modelo de substituição e descontextualização (Zona Muda) 64

7 DISCUSSÃO 66

8 CONSIDERAÇÕES FINAIS 75

REFERENCIAS 77
APÊNDICES.. 84
ANEXOS 95

1 INTRODUÇÃO

A transmissão do Vírus da Imunodeficiência Humana (HIV) causador da Síndrome da Imunodeficiência Adquirida (SIDA) configura-se como uma realidade significativa diante da dimensão da sua problemática, e do contexto das políticas de saúde pública que o envolvem (GUIMARÃES *et al.*, 2017; COUTINHO *et al.*, 2018).

Segundo o Programa Conjunto das Nações Unidas sobre HIV/Aids, a estatística global do ano de 2019, foi de 38 milhões de pessoas vivendo com HIV (UNAIDS, 2020), e pode-se dizer, uma cifra alarmante se considerarmos as características da doença e sua alta virulência e transmissibilidade, que implica em tratamento especializado e de alto custo, em contextos de saúde dos mais variados, exigindo prioridade e empenho das instituições e políticas públicas de saúde, no mundo.

No ano de 2019, o número de notificações de novos casos de infecção pelo HIV foi de 41.909 casos, e de maneira mais abrangente, o Ministério da Saúde (MS), apresentou um panorama referente ao período entre 2007 até junho de 2020, indicando um número de 342.459 casos de infecção pelo HIV, desse total, 152.029 (44,4%) concentra-se na região Sudeste, 68.385 (20,0%) na região Sul, 65.106 (19,0%) na região Nordeste, 30.943 (9,0%) na região Norte e 25.966 (7,6%) na região Centro-Oeste. Verifica-se o maior número de casos nas regiões Sudeste e Sul, mas com crescimento considerável nas regiões Nordeste e Norte se comparado aos anos anteriores (BRASIL, 2020).

Ainda com referência aos dados publicados pelo MS, no mesmo período citado, com referência à idade, observa-se uma maioria nos casos de infecção pelo HIV, na faixa etária entre 20 e 34 anos e um percentual de 52,7% dos casos, o que denota a configuração de uma população jovem. E, quando relacionado ao sexo, os números revelaram um total de 237.551 (69,4%) casos em homens e 104.824 (30,6%) casos em mulheres (BRASIL, 2020), indicando índices elevados com relação a mulher.

Apesar desses indicadores demonstrarem a transmissão elevada entre pessoas do sexo masculino, o aumento desses índices em mulheres vivendo com HIV, durante as duas últimas décadas tomou espaço e destacou-se nos aspectos epidemiológicos, bem como entre a população heterossexual (BRASIL, 2020), podendo-se destacar entre os principais fatores determinantes, um aumento na adoção de práticas sexuais desprotegidas (DUARTE; ROHDEN, 2019). Tratam-se de

variáveis cujo perfil, revela uma problemática que direciona o foco de atenção para além dos sujeitos considerados população-chave, como o público heterossexual e a população jovem.

Referente aos contextos regional e local, num período mais posterior, entre 2008 e 2018, o Rio Grande do Norte (RN), Estado brasileiro situado na região nordeste do país, apresentou aumento significativo do número de casos detectados com HIV num total de (81,7%). Em 2018, para a média nacional de casos detectados, registrou-se um número de 17,8% de casos a cada 100 mil pessoas, e nesse caso, o Estado do RN chegou a 20,9%, situando-se na décima posição, em relação aos estados de maior índice de detecção do vírus, e que ressalta-se, apresentou uma média maior que a do índice nacional (BRASIL, 2019).

Nessa direção, entende-se ser de muita relevância a análise desses dados, levantados e divulgados pelas Instituições de saúde, resultados de pesquisas científicas, bem como, das práticas assistenciais e/ou intervenções realizadas pelos profissionais, por tratar-se de uma infecção crônica, que gera implicações direta nos hábitos de vida dos sujeitos acometidos.

Com a evolução e desenvolvimento das políticas assistenciais destinadas aos usuários infectados pelo HIV, assim como, pela aids, tem sido possível obter ações efetivas que permitem melhorar a condição de vida da pessoa vivendo com HIV/aids. E, como resultantes, programas e protocolos foram instituídos como foi o caso do Programa Nacional de Doenças Sexualmente Transmissíveis e Aids (PN-DST/Aids), e também, da introdução da terapia antirretroviral (TARV), com acesso gratuito e universal (BRASIL, 1999; GUIMARÃES *et al.*, 2017; BRASIL, 2018a). Atualmente, reconhece-se que o TARV, desfruta de uma potente atuação na diminuição da carga viral, e aumento do número das células de defesa do organismo, vislumbrando atingir a carga viral indetectável (BRASIL, 2018a).

Na busca pelo aprimoramento das ações de saúde, além de buscar a diminuição da morbimortalidade da infecção, foi possível visualizar o aumento da adesão dos indivíduos a terapêutica medicamentosa, o que corroborou significativamente com a diminuição da transmissão do vírus (HALLAL *et al*., 2015; SILVEIRA; LAZZAROTTO, 2018).

No contexto de atenção à saúde da pessoa vivendo com HIV, insere-se os Serviços de Assistência Especializada (SAE) em Infecções Sexualmente Transmissíveis IST/HIV/Aids. Tais serviços, juntamente com a Atenção Primária à

Saúde (APS), são os responsáveis por promover a contínua assistência à saúde dessa população (BRASIL, 2018). No entanto, se observa que os SAE constituem-se como principal modalidade assistencial responsável pelo acompanhamento dos usuários acometidos por essa condição crônica e transmissível de saúde (BRASIL, 2018a). Suas principais características, são o foco na resolubilidade, no diagnóstico e terapêutica em nível ambulatorial e, favorecendo a organização da atenção à saúde das pessoas vivendo com HIV (MAGNABOSCO *et al.*, 2018).

Diante dos avanços científicos, evolução do tratamento e o empenho dos serviços de saúde em operacionalizar as medidas de prevenção e controle, é evidente o aumento na sobrevida das pessoas vivendo com HIV, o que implicou na mudança de alguns conceitos e caracterização de alguns aspectos que envolvem a temática do HIV (TEIXEIRA *et al.*, 2018).

O vírus-HIV, antes encarado como uma infecção mortal, permite hoje aos sujeitos conviverem com essa infecção de caráter crônico, em circunstância mais otimista e equilibrada no processo saúde-doença. É fato que, muito dessas pessoas, acometidas pelo HIV sentem-se seguras junto a sua condição crônica de saúde, com possibilidades de viver experiências, antes impossível, em que se quer mencionava-se falar a respeito, nos serviços de saúde (SOUZA NETO *et al*., 2016), sobre vivências de relacionamentos afetivos entre parceiros com o mesmo status sorológico que o seu, ou distintos (OLIVEIRA *et al*., 2020).

Dentre algumas das estratégias estabelecidas pela atual política de atenção a pessoa portadora do vírus-HIV, encontra-se a diretriz TARV, que tornou possível tais vivências, e teve avanços significativos como estratégia preventiva, à qual alcançou de maneira global, o conceito de o indetectável, ser igual a intransmissível, isto é, (I=I), relacionada à pessoa que vive com HIV em TARV no tempo mínimo de seis meses (BRASIL, 2019; SILVA, DUARTE, LIMA, 2020).

Os SAE têm acolhido constantemente usuários envolvidos afetivamente e sexualmente com parceiros que possuem sorologia diferente da sua, seja por meio da pessoa que vive com HIV ou dos indivíduos que buscam a Profilaxia Pré-Exposição (PREP) ou a Profilaxia Pós-Exposição (PEP) (FERNANDES *et al.*, 2017). Tal condição, que envolve, tanto o tipo de relação entre parceiros homossexuais quanto heterossexuais, constitui-se no que se denomina de sorodiferença, sorologia mista, sorodivergência ou ainda sorodiscordância para o HIV (FLEISCHER, 2013).

Apesar do termo sorodiscordância ser utilizado amplamente em estudos sobre

esse fenômeno, e o termo ainda constar no guia de terminologia do Programa Conjunto das Nações Unidas sobre HIV/Aids (UNAIDS), estudos recentes relatam a preferência pelo uso do termo “sorodiferença”, em virtude do primeiro termo remeter a ideia conflituosa de “discordância” entre os parceiros. Ideia esta, que ao visualizar a ressignificação do contexto de saúde e doença do HIV, possa gerar discussões conflituosas no avanço e ultrapassagem de algumas barreiras sociais (FLEISCHER, 2013).

Com a descoberta dessa condição de parcerias sexuais em sorodiferença, os parceiros se veem diante de uma realidade que requer mudanças em seu comportamento, sobretudo no que se refere aos hábitos das práticas sexuais (NGURE *et al*., 2016), e ao uso do preservativo que consiste em uma das mais importantes ferramentas no combate a transmissão do HIV. Trata-se de uma temática evidenciada pelos estudos que refletem sobre as dificuldades enfrentadas, por se constituir de uma prática, na maioria das vezes, ainda não adotada pelos parceiros antes do diagnóstico (TAYLOR, 2016; OJIKUTU *et al.*, 2016).

Embora, a infecção pelo HIV seja um tema relevante e já bastante difundido, ocupando amplo espectro de ação nos serviços de saúde pública, os estudos que envolvem essa IST, em geral, evidenciam uma preocupação mais focalizada na prevenção da transmissibilidade do vírus, em detrimento dos temas relacionados à soropositividade e a sexualidade em sua totalidade (MAKSUD, 2012; GREENER *et al.*, 2018).

Nos relacionamentos sexuais com parceiros sorodiferentes para o HIV, os envolvidos se veem diante de novos desafios, como o receio em transmitir o vírus ao parceiro soronegativo, ou o medo da não aceitação da sua condição sorológica (PATEL *et al.*, 2018). São sentimentos e/ou sensações, que se afloram, quando acompanhados por um desequilíbrio psicológico e emocional, por compreender que a sexualidade não direciona seu conceito apenas ao ato sexual, mas abrange as características humanas que tem a natureza de se ligar às pessoas, ao prazer, aos desejos, às necessidades, à vida (FERNANDES *et al.*, 2017; REIS; GIR, 2010). Este contexto é partilhado cotidianamente pelos sujeitos para os profissionais de saúde que os assistem, envolvendo-os de forma direta quanto aos seus anseios e expectativas.

A literatura atual evidencia uma reflexão acerca da assistência a esses casais, que continuamente estão presentes no serviço, mas que na maioria das vezes, não são percebidos e/ou legitimados pelos profissionais de saúde, o que torna crescente

o apagamento simbólico retratado quando se trata da sorologia mista entre casais (LAGO; MAKSUD; GONÇALVES, 2013).

Tal distanciamento entre essas circunstâncias apresentadas e o posicionamento a respeito, por parte dos profissionais, pode ser gerado muitas vezes em decorrência da lacuna de conhecimento advinda da formação desses profissionais, que acabam tornando limitada a atenção à saúde dada a esse público. Além disso, há também, por parte dos profissionais, questões pessoais, culturais e sociais de que a sexualidade constitui algo sensível e particular dos parceiros, e que pode comprometer os desdobramentos de um cuidado efetivo (SANTOS *et al*., 2019; COLACO *et al*., 2019).

Algumas realidades mostraram as dificuldades inerentes à fuga do olhar biopsicossocial, fator este que resulta em abordagens profissionais cada vez mais tecnicistas em que os sujeitos são vistos apenas como objetos de intervenção, e que exclui constantemente suas singularidades (FERNANDES *et al.*, 2017).

A realidade do preconceito, a presença ainda enraizada dos estigmas, e a falta de conhecimento que constantemente cercam esse assunto, torna a compreensão e aceitação do fenômeno da sorodiferença, bem distantes daquilo do que se considera normal e aceitável para a sociedade, favorecendo a conduta do sigilo adotado frequentemente pelos parceiros quanto a sorologia mista, e em consequência, a diminuição do vínculo do parceiro soronegativo com os serviços de saúde (SAID; SEIDL, 2015; ALBUQUERQUE; BATISTA; SALDANHA, 2018).

É válido mencionar, a resistência de grande parte dos profissionais de saúde em reconhecer a inserção do parceiro soronegativo como usuário do serviço, assim como o parceiro HIV positivo, o que configura-se num contraste diante das recomendações propostas pela política de assistência, quando muitas vezes, visualizam-se as pessoas que vivem com HIV, apenas como sujeitos que precisam de cuidados seja no contexto físico, social ou psicológico (LAGO; MAKSUD; GONÇALVES, 2013; DANTAS *et al*., 2015).

Embora a literatura aponte as fragilidades apresentadas quanto a atuação dos profissionais de saúde no contexto ora descrito, diante das dúvidas, vulnerabilidades e medo, os profissionais de saúde desempenham um papel decisivo e substancial na construção de vínculos, informações, cuidados e acolhimento aos parceiros. Pois, através de suas funções e responsabilidades, eles podem ter grande participação na disseminação do conhecimento relacionado e favorecer a desconstrução de preconceitos enraizados que envolvem as relações sorodiferentes (MAJOR *et al.*,

2017).

Nessa perspectiva, destaca-se a necessidade em acessar a percepção dos profissionais de saúde acerca dos aspectos que envolvem o contexto da sorodiferença, uma vez que são os responsáveis pela oferta do cuidado em sua dimensão mais ampla, perpassando o seguimento afetivo, emocional, familiar e social, que tem como princípio a integralidade e não apenas a execução de procedimentos para promover o conforto ou atender à necessidade dos pacientes (FERNANDES *et al.*, 2017; SILVA; GUEDES, 2017).

Quero aqui destacar, que o meu interesse e a aproximação com o fenômeno da sorodiferença para o HIV, iniciou-se em meu curso de graduação e a partir das vivências em uma pesquisa de campo, junto as pessoas com HIV vivendo em parceria sexual sorodiferente. Por meio dessa experiência, pude acessar conteúdos que expressaram dentre outros resultados a insatisfação no que tange a alguns aspectos inerentes a assistência prestada pelos serviços de saúde, como a falta de interesse dos profissionais de saúde em questionar a presença da sorodiferença e/ou como esta é vivenciada pelos parceiros (SILVA *et al.*, 2018).

Assim, a partir da problemática descrita, e das inquietações provadas pela leitura e experiencias acadêmicas e profissionais, percebeu-se a necessidade de analisar o significado atribuído as pessoas em sorodiferença para o HIV, entre os profissionais de saúde responsáveis por todo o acompanhamento terapêutico dos sujeitos envolvidos.

Para tanto, a referida pesquisa ancora-se nos pressupostos teóricos e metodológicos da Teoria das Representações Sociais (TRS) segundo a vertente de Jean Claude Abric, com o propósito de analisar a subjetividade acerca da seguinte questão: Quais as representações sociais das pessoas em sorodiferença para o HIV elaboradas por profissionais dos Serviços de Assistência Especializada IST/HIV/Aids?

O estudo de Representações Sociais (RS) tem contribuição direta com a geração de debates em torno de um fenômeno, e a escolha em tornar evidente a temática da sorodiferença sob o olhar do profissional de saúde de serviços especializados, consiste na tentativa de tornar as representações sociais obtidas, objeto estruturante na contribuição do processo de desmistificação de preconceitos e estigmas enraizados pela sociedade e presentes no contexto dos serviços de saúde. Além de também contribuir para uma fundamentação teórica e reflexiva, de maneira que, possa auxiliar nas ações voltadas para o público que vive a sorodiferença e no

aperfeiçoamento profissional (LAGO; MAKSUD; GONÇALVES, 2013; MACHADO *et al*., 2016).

2 OBJETIVOS

2.1 OBJETIVO GERAL

Analisar as Representações Sociais na vertente estrutural das pessoas em sorodiferença para o HIV, elaboradas por profissionais dos Serviços de Assistência Especializada em IST/HIV/Aids.

2.2 OBJETIVOS ESPECÍFICOS

- Identificar a estrutura das Representações Sociais na normalidade e zona muda das pessoas em sorodiferença para o HIV, elaboradas pelos profissionais do SAE IST/HIV/Aids;
- Compreender a estrutura e os significados representacionais da sorodiferença para profissionais do SAE IST/HIV/Aids.

3 REVISÃO DE LITERATURA

Este capítulo apresenta os conceitos e/ou pré-conceitos, bem como, algumas representações sociais relacionadas aos comportamentos e dificuldades presentes em torno do tema sorodiferença, e sua inserção no contexto e vivência de pessoas que convivem com o HIV. Constituiu-se num levantamento da literatura sobre o assunto, além dos programas, protocolos e manuais do Ministério da Saúde, que normatizam e orientam o atendimento aos usuários em sorodiferença, quando da assistência prestada pelos profissionais de saúde nesses serviços.

Assim, o capítulo foi organizado em tópicos, que representam os principais aspectos que envolvem, os indivíduos que vivenciam a sorodiferença e a atenção dada pelos profissionais dos serviços de assistência especializada, no Sistema Único de Saúde, Quais sejam: 3.1 - O conceito da sorodiferença para o HIV e as dificuldades enfrentadas pelos parceiros; 3.2 - Os Serviços de Assistência Especializada e o atendimento aos usuários sorodiferentes; 3.3 - A atuação da Enfermagem no atendimento aos usuários sorodiferentes.

3.1 O CONCEITO DA SORODIFERENÇA E AS DIFICULDADES ENFRENTADAS PELOS PARCEIROS

Por ser uma infecção de caráter crônico e tratamento longo de alto custo, a atenção e o acolhimento as parcerias em sorodiferença para o HIV são vistas cada vez mais com uma importância fundamental pois, viabiliza possibilidades para a melhoria da qualidade de vida ao considerar as relações afetivas e sexuais dessa natureza (SOUZA NETO *et al*., 2016).

Tais parcerias passaram a chamar atenção da comunidade científica e dos serviços de saúde apenas por volta dos anos 90 e 2000, já que anteriormente, o foco e a construção de sentidos da pessoa com HIV na sociedade eram inteiramente relacionadas a ideia de uma peste incurável, a qual sentenciava os infectados a morte (LAGO; MAKSUD; GONÇALVES, 2013; FERNANDES *et al*., 2017).

Assim, uma pessoa considerada saudável relacionar-se com outra, acometida pelo HIV gerava estranheza, medo, tensão, estigmas e preconceitos, ao atribuir como inaceitáveis e fora do padrão da sociedade essas relações, talvez pelo fato de ainda desconhecerem as possibilidades preventivas e assistenciais que poderiam favorecer a relação sorodiferente (MAKSUD, 2012).

Além disso, a associação do HIV a determinados grupos da sociedade como

homossexuais, profissionais do sexo e usuários de drogas injetáveis, colaborou para o enraizamento de estigmas dessa infecção às pessoas com comportamentos ditos promíscuos (COLACO *et al*., 2019).

As parcerias sorodiferentes estão cada vez mais presentes nos serviços de saúde, na busca por um cuidado contínuo e pelas estratégias que possam ir ao encontro de suas necessidades. É importante dizer, tratar-se de um contexto movido por crenças religiosas que acreditam em uma cura futura, ou mesmo numa evolução da ciência que dentre os grande alcances, já proporcionou a redução da carga viral, a disposição de métodos preventivos eficazes, com a melhoria da qualidade de vida da pessoa que vive com HIV através da adesão ao TARV, (AMORIM; SZAPIRO, 2008).

Diferentes circunstancias envolvem a sorodiferença para o HIV, uma delas atribui-se as dificuldades que os parceiros vivenciam para lidar com essa diferença sorológica (AMORIM, SZAPIRO, 2008; SILVA *et al*., 2018). Por se tratar de uma infecção que tem a via sexual como protagonista para a contaminação, a manutenção do sexo seguro torna-se um desafio para que a infecção não passe a ser uma realidade biológica para ambos (REIS; GIR, 2005; SILVA *et al*., 2018).

O medo da transmissão do vírus ao parceiro soronegativo, é consenso entre ambos os parceiros, daí a importância da revelação precoce do diagnóstico, uma vez que, diante desses contextos, pessoas soronegativas são mais propensas a fazer uso do preservativo de forma consciente (KENYON *et al*., 2015).

Mesmo que os parceiros considerem importante revelar o diagnóstico para seus companheiros, essa ação pode ser encarada como um momento dotado de ansiedade e receios e que em muitos casos é retardado, diante da possibilidade da não aceitação do parceiro soronegativo, e pela exposição de sua intimidade, a qual carrega muitos estigmas se tratando do HIV (SILVA *et al*., 2018; OLIVEIRA *et al*., 2020).

Além disso, em se tratando de casais que mantém uma união estável, a revelação do diagnóstico pode trazer à tona sentimentos ainda mais negativos, uma vez que, além do parceiro soronegativo lidar com a infecção do vírus, também terá que lidar com a infidelidade do parceiro, o que torna ainda mais difícil essa revelação (SÁ; SANTOS, 2018)

O sigilo ainda é percebido diante da família e o círculo social dos parceiros, onde encontram por meio dessa estratégia uma maneira de driblar a reprovação e críticas que surgem no cotidiano (AMORIM; SZAPIRO, 2008; MAKSUD, 2012).

Nesse âmbito, o sexo seguro torna-se um desafio diante das dificuldades na negociação para mudanças de comportamentos e/ou, adesão de métodos preventivos, como é o uso do preservativo (SILVA *et al*., 2018). Apesar do uso regular do preservativo ser considerado como medida preventiva com alta relevancia no controle da contaminação, nem sempre é encarado com facilidade, ante as barreiras culturais, educacionais, comportamentais e emocionais que, na maioria das vezes, o associam a uma condição limitante do prazer e da intimidade (GUIRA *et al*., 2013; SAID; SEIDL, 2015; SILVA *et al*., 2018; REIS *et al*., 2019).

Além disso, a não adesão ao uso do preservativo pode ser apontado como estratégia diante do desejo de ter filhos (GUIRA *et al*., 2013). Em contrapartida, outro fator apontado pela literatura, atrela-se ao fato de também existir a adoção dos métodos permanentes de esterelização, e nesses casos as parcerias dispensam o uso do perservativo, por relacioná-lo apenas ao uso do método contraceptivo e não como uma prevenção às IST (MARFATIA *et al*., 2013).

3.2 OS SERVIÇOS DE ASSISTÊNCIA ESPECIALIZADA E O ATENDIMENTO AOS USUÁRIOS SORODIFERENTES

Em geral, as parcerias sorodiferentes são usuários dos Serviços de Saúde que demandam um acompanhamento a longo prazo, diante do efeito crônico que a infecção pelo HIV se constitui (SAHANA; BETKERUR, 2019). O estabelecimento do vínculo e um diálogo acolhedor, são ações cruciais para favorecerem o processo de inserção do parceiro soronegativo nos serviços que muitas vezes não se reconhece como alguém que precisa ser contemplado no processo terapêutico do parceiro (LUZ; MIRANDA; TEIXEIRA, 2010).

Razão pela qual, essas pessoas buscam nos serviços de saúde a melhor forma de conviver com as nuances que podem apresentar como riscos para ambos, sejam nos aspectos físicos, psicológicos ou sociais (SAID; SEIDL, 2015).

Os SAE juntamente com as Unidades Básica de Saúde da APS são os serviços responsáveis por acolherem e acompanharem es pessoas que vivem com HIV, e por consequência as parcerias sorodiferentes. Nesse sentido, a APS compartilha com os SAE, a oferta de cuidados e se configura como porta de entrada no sistema de saúde, para acolher pessoas que vivem com HIV e suas parcerias, fazer testagem rápida e aconselhamento para o parceiro soronegativo, bem como, assistí-los em suas necessidades nas consultas, atividades educativas, e no referenciamento para o

serviço especializado (BRASIL, 2006; BRASIL, 2017).

No entanto, os serviços especializados atuam de forma predominante, visto que a realidade das unidades básicas de saúde, no nível de Atenção Primária, no contexto brasileiro, ainda não firmou oficialmente um fluxo de atendimento e acompanhamento a essas pessoas de forma efetiva (COLACO *et al*., 2019).

Os SAE são os responsáveis por receber, acolher e prestar a necessária atenção à saúde dessas parcerias. A oferta da PREP, distribuição do TARV, as consultas periódicas, o encaminhamento para atendimentos especializados, aconselhamento, testagem, distribuição de insumos preventivos, são ações que contemplam as parcerias sorodiferentes nesse serviço (BRASIL, 2018a; MAGNABOSCO *et al.*, 2018).

No que compete a oferta de cuidados, um dos pilares da atenção a saúde das parcerias sorodiferentes consiste na prevenção, tida como principal estratégia de manutenção do status sorológico misto, afim de proporcionar proteção às práticas sexuais que deve ser incentivada pelos serviços de saúde (MARFATIA *et al*., 2013; BRASIL 2018a; SILVA *et al*., 2018).

O Minitério da Saúde em 2017, estabeleceu entre as diretrizes da política de atenção ao tratamento e controle do vírus HIV, a prevenção combinada, que adota tecnologias preventivas em três níveis diferentes de abordagens (biomédica, estrutural e comportamental), como estratégia de cuidado a ser incorporada nos serviços de saúde, aos quais destinam-se a tentativa de compreensão dos sujeitos em suas singularidades e especificidades (SILVA; CAMARGO JUNIOR, 2011; BRASIL, 2017).

A abordagem biomédica, está centrada no estímulo a busca das estratégias que evitam o contato com material biológico contaminado (métodos de barreira) e o tratamento como prevenção (TCP) por meio do uso dos antirretrovirais. Já a abordagem na dimensão estrutural, contempla o cuidado promovido por meio das transformações socioculturais no intuito de minimizar e combater as vulnerabilidades, a exemplo do preconceito.

Em relação a abordagem comportamental, há uma intervenção no contexto da disseminação de informações e ampliação do acesso, que envolve as abordagens preventivas individuais e coletivas, como a testagem, o aconselhamento, bem como as estratégias educativas por pares (BRASIL, 2017; BRASIL, 2018a).

A abordagem da prevenção combinada em suas três dimensões possibilita que

o profissional de saúde consiga direcionar o cuidado preventivo aos parceiros sorodiferentes levando em consideração variadas estratégias, prezando fundamentalmente as individualidades inerentes a cada contexto (KING *et al*., 2012; BRASIL, 2018a).

A sua implementação, configura-se nos aspectos biopsicossociais dirigidas as práticas sexuais seguras, envolvendo os parceiros por meio das tecnologias preventivas, como preservativos, PREP, PEP, adesão ao TARV, testagem regular, a promoção da educação em saúde dos parceiros e o apoio a desmistificação de conceitos pré-estabelecidos, incentivo ao vínculo com os serviços, e a contribuição com a educação do meio social, no qual se inserem os parceiros com vistas ao enfrentamento dos estigmas e a descriminação (SILVA; GUEDES, 2017; BRASIL, 2018a; JUGA *et al*., 2020).

Identifica-se que a adesão ao TARV é um grande sucesso, e atualmente contribui para que o novo conceito, de I = I, (Indetectável = Intransmissível) seja cada vez mais difundido nos serviços de saúde e entre os usuários, a qual, estabelece que pessoas vivendo com HIV em terapia antirretroviral e carga viral indetectável, pelo menos há seis meses, não transmite o vírus HIV por via sexual (BRASIL, 2019).

Trata-se de uma estratégia preventiva já reconhecida como consenso nacional e internacional, que se mostra como avanço positivo para as relações sorodiferentes, por ampliar as possibilidades preventivas que o tratamento proporciona (BRASIL, 2019; CALABRESE, MAYER, 2020; SILVA, DUARTE, LIMA, 2020).

Nessa perspectiva, o aconselhamento como estratégia preventiva na sorodiferença, revela possibilidades proveitosas para a orientação adequada quanto a adoção dessas estratégias preventivas, e escuta qualificada (BRASIL, 2018a). E, na realização da testagem do parceiro soronegativo, o aconselhamento pré e pós testagem é fundamental para que seja compreendido os riscos e as chances de contaminação (KENYON *et al*., 2015).

Além disso, no aconselhamento os parceiros têm a oportunidade de receber orientações quanto ao desejo de concepção ou anticoncepção, educação sexual em saúde, além das questões específicas à sua situação de saúde (BRASIL, 2018a).

A PREP, profilaxia que permite o uso de antirretrovirais por pessoas soronegativas para reduzir o risco de infecção pelo HIV, se direciona a alguns grupos específicos, sendo apontados como melhores candidatos para seu uso pessoas que vivem relacionamentos sorodiferentes para o HIV. A PREP é vista com esperança e

fortalece a segurança para se viver a sorodiferença, sobretudo, quando integrada a outras formas preventivas como a adesão do TARV pelo parceiro HIV positivo (WARE *et al*., 2012; TELLALIAN *et al*., 2013; HALLAL *et al*., 2015; MORTON *et al.*, 2017; BRASIL, 2018b).

A PEP consiste em outra abordagem biomédica, na qual busca a prevenção de parceiros soronegativos através do uso de antirretrovirais após a exposição a um risco de contaminação (BRASIL, 2018c)

Mesmo diante das dificuldades apontadas quanto ao uso do preservativo como método de prevenção, a adoção dessa estratégia é uma das mais utilizadas pelos parceiros nas práticas sexuais, diante do predomínio prescritivo dos profissionais de saúde em recomendar o seu uso (SILVA *et al*., 2018).

É importante que o processo para a escolha dos métodos preventivos proteja o sexo e, sendo algo consensual entre os parceiros, levando em consideração aspectos de gênero, conjugabilidade e, sobretudo, o processo de entendimento do que seja a infecção pelo HIV entre os parceiros (MAKSUD, 2009).

3.3 A ENFERMAGEM NO ATENDIMENTO AOS USUÁRIOS SORODIFERENTES E OS DESAFIOS VIVENCIADOS NO CONTEXTO DOS PROFISSIONAIS DE SAÚDE

Dentre as áreas profissionais atuantes junto as pessoas que vivem em sorodiferença para o HIV, a enfermagem apresenta funções relevantes. Dentre as principais atividades cita-se: a realização de atividades e ações relacionadas ao planejamento familiar, a consulta de enfermagem voltada para a pessoa que vive com HIV, bem como, para os usuários da PREP, além de atuar diretamente no acolhimento e aconselhamento dessas parcerias, nas consultas, além das atividades educativas sobre temas relacionados a sexualidade (LANGENDORF *et al*., 2017; REIS *et al*., 2016).

Um outro papel relevante da enfermagem, nessa área específica de atenção em saúde apontado pela literatura, é a sua colaboração na desconstrução de mitos e tabus que estigmatizam e legitimam o preconceito que é visto perante as pessoas que vivem a sorodiferença para o HIV (LANGENDORF *et al*., 2017).

É bem verdade, que há necessidade de melhorias quanto a atuação dos profissionais de saúde, e mais serviços com ações e projetos específicos para parcerias junto a usuários em sorodiferença. Observa-se que esse fato, já é um

dilema consensuado na literatura e em contextos diversos, não apenas o brasileiro, ao evidenciar haver um déficit de conhecimento aprofundado dos mecanismos e fatores que circundam a temática da sorodiferença (REIS; GIR, 2010; SILVA; CAMARGO JUNIOR, 2011; GREENER *et al*., 2018).

Percebe-se, ainda haver uma visão limitada por parte dos profissionais, em que o cuidado a pessoa que vive com HIV é centralizado em intervenções baseadas no modelo de cuidado biomédico, no qual a busca pelo bem estar biológico supera a dimensão dos demais cuidados, indo de encontro as demandas psicossociais que em muitos casos se apresentam como prioritárias nos relacionamentos dessa natureza (REIS *et al*., 2019).

Nesse âmbito, se apresentam questões a respeito da integralidade do cuidado, cuja inserção como eixo central numa assistência à saúde contempla as pessoas que vivem a sorodiferença. Visto que, ressaltam as possibilidades do olhar profissional que transcenda a esfera biológica, e leve em consideração aspectos psicoafetivos e sociais que implicam na relação entre sorodiferentes, tais quais: a educação sexual, a escuta sensível ante os medos e dúvidas, e a identificação de suas vulnerabilidades (LAGO; MAKSUD; GONÇALVES, 2013; SOUZA NETO; SILVA; RODRIGUES, 2016; SILVA *et al*., 2018; BOA *et al*., 2018).

Além disso, existem as dificuldades vivenciadas por parte desses profissionais, no que se refere a abordagem dos aspectos psicoemocionais dessa assistência, à exemplo, quando da testagem e comunicação de resultados de diagnósticos positivos para o vírus HIV, sobretudo quando envolve aspectos sensíveis à sexualidade (DANTAS *et al*., 2015; ZAKABI, 2017).

A não inserção do parceiro soronegativo como um usuário que também demanda cuidados e o direcionamento de projetos e ações que lhes sejam inerentes, é outra fragilidade apontada pela literatura, desligitimando a sorodiferença na amplitude que evolve a assistência a pessoa que vive com HIV (LAGO; MAKSUD; GONÇALVES, 2013).

Nesse sentido, aponta-se como fragilidade o tratamento dessas questões de forma tímida ou ainda inexistente no aconselhamento feito pelos profissionais de saúde, uma vez que a falta de proximidade com o conhecimento dessa natureza possa ser um fator a ser considerado (WEST *et al*., 2016; GUTIN *et al*., 2020).

Outro aspecto destacado pela literatura e revelado como justificativa pelos profissionais de saúde, quanto a questão, se importar pouco sobre questões como, a

concepção e fertilidade no acompanhamento dessas parcerias, está relacionado a percepção sobre do pouco conhecimento sobre esse tipo de informação, por parte dos parceiros, pois observa-se que as questões psicológicas, intelectuais e financeiras podem interferir, numa forma consciente de receber essas informações (WEST *et al*., 2016).

Assim, entende-se a necessidade busca pela capacitação profissional, a fim de proporcionar um atendimento embasado em um conhecimento específico e ampliado, de maneira que contribua na melhoria do atendimento as parcerias em sorodiferença. Só assim, ações específicas e diretas a esse público poderão viabilizar estratégias que possam contribuir para relações sorodiferentes mais conscientes. (REIS; GIR, 2010; SILVA; CAMARGO JUNIOR, 2011; CRANKSHAW *et al*., 2014).

Daí, ser importante haver incentivos na busca pelo conhecimento novo e atualizado, como forma de capacitação para a prestação de um atendimento eficaz, qualificado e combativo na luta contra o preconceito, infelizmente, ainda presente nos serviços de saúde, e até mesmo nos centros especializados (LAGO; MAKSUD; GONÇALVES, 2013). Os profissionais de saúde não estão isentos de colocarem sentimentos e valores acima do compromisso assumido em atender integralmente usuários independente de suas escolhas, doenças ou comportamentos (ZAKABI, 2017; LAGO; MAKSUD; GONÇALVES, 2013).

O acolhimento e o vínculo construído com os profissionais, o cuidado humanizado e uma assistência sem descriminação, são aspectos considerados importantes para os parceiros sorodiferentes, que buscam ajuda nos serviços de saúde, onde a ausência do preconceito é crucial para se vencer os estigmas persistentes em grande parte dos serviços de saúde. (SAID; SEIDL, 2015).

A educação permanente consiste em uma estratégia otimista que deve ser incentivada nos serviços de saúde, afim de combater algumas fragilidades programáticas ou institucional, encaradas como o tipo de vulnerabilidade na qual, a atuação do serviço de saúde pode impor barreiras na adesão de práticas que favoreçam melhorias no processo de saúde-doença dos usuários (REIS; NEVES; GIR, 2013; LAGO; MAKSUD; GONÇALVES, 2013; SOUZA NETO; SILVA; RODRIGUES, 2016; FERNANDES *et al*., 2017).

Nesse sentido, o desenvolvimento de ações planejadas e direcionadas para esse público, podem ser acolhidas conforme as necessidades e especificidades, indo de encontro às fragilidades mencionadas, fortalecendo a adesão e vínculo dessas

parcerias com os serviços de saúde (SILVA; CAMARGO JUNIOR, 2011; SOUZA NETO; SILVA; RODRIGUES, 2016).

A infecção pelo HIV durante muito tempo, carregou consigo diversos conceitos e significados no contexto social atrelados a sentimentos devastadores, associados a morte e sofrimento, ocupando o lugar de doença mais estigmatizando da sociedade, sobretudo nos anos iniciais ao seu descobrimento em 1981 (BARBARÁ; SACHETTI; CREPALDI, 2005). Nesse sentido, a Teoria das Representações Sociais se tornou uma importante estratégia para acessar questões importantes relacionados a prevenção, os mecanismos de transmissão, diante da complexa problemática em que se apresentava o HIV e a aids (BARBARÁ; SACHETTI; CREPALDI, 2005).

Atualmente, o HIV continua sendo utilizado amplamente como objeto de estudo das representações sociais em diversos contextos, como por exemplo, qualidade de vida vista por pessoas que vivem com HIV, representações de transexuais, profissionais do sexo, idosos, adolescentes, acerca do HIV/aids, bem como relacionado aos aspectos da espiritualidade vivida pelos sujeitos que vivem com o vírus, dentro outros públicos e contextos (GOIS *et al*., 2020; ABREU *et al*., 2020; BRANDÃO *et al*., 2019; CASTRO *et al*., 2019; FRANÇA *et al*., 2020).

Um cenário particular ainda é visto ao pensar nas RS e o campo do HIV/aids, quando esta temática é observada sob o olhar dos profissionais de saúde, em que se é possível alcançar as percepções e/ou conhecimentos construídos, além de possibilitar a identificação de necessidades e dificuldades que possam ser desveladas e assim, contribuir com o redirecionamento das práticas de saúde e solidificação de políticas de enfrentamento (SANTOS *et al.*, 2019).

As RS retratadas por essa população acerca de um determinado objeto configura-se pela forma como constroem, percebem e lidam com o fenômeno em questão, estando relacionada à sua visão de mundo, em grande parte influenciada por atitudes, crenças e valores construídos socialmente, promovida pelo envolvimento de informações inerentes ao senso comum, bem como, ao pensamento científico (MOSCOVICI, 2007). A partir da interação de ambos, se permite acessar quais representações permeiam e motivam suas percepções e atividades desenvolvidas no ambiente de saúde (SANTOS *et al*., 2017; MACHADO *et al*., 2016).

A literatura ressalta a importância da reflexão sobre os significados dados pelos profissionais de saúde sobre diferentes contextos relacionados ao HIV/aids, a exemplo, das práticas de saúde e o cuidado às pessoas que vivem com o vírus

(ANGELIM *et al*., 2019; SUTO *et al*., 2017), os testes rápidos para HIV na atenção básica (ALMEIDA JUNIOR *et al*., 2018) e aos aspectos relacionados à conjugabilidade no contexto do HIV diante da sorodiferença (DANTAS *et al*., 2015; LAGO; MAKSUD; GONÇALVES, 2013).

Nessa perspectiva da sorodiferença para o HIV, seja na relação entre os parceiros, no ambiente familiar, nos grupos sociais, ou no ambiente de saúde, em todos estes, ela se comporta como objeto de reflexão para o melhor entendimento das relações sociais que a configuram (LAGO; MAKSUD; GONÇALVES, 2013; MMEJE *et al*., 2016).

4 REFERENCIAL TEÓRICO

Este capítulo se constitui por alguns princípios teóricos das Representações Sociais, e de alguns fundamentos da Teoria do Núcleo Central (TNC) de Jean Claude Abric (ABRIC, 2003) em complementariedade, que configuram as representações sociais a partir de uma estrutura que agrega significados, interpretados sob a perspectiva cognitivo-estrutural denominada de Teoria do Núcleo Central (ABRIC, 2003).

4.1 O UNIVERSO DA TEORIA DAS REPRESENTAÇÕES SOCIAIS (TRS)

As Representações Sociais (RS), segundo o conceito do psicólogo romeno naturalizado francês, Serge Moscovici, deve ser pensada a partir de um conjunto de conhecimentos práticos do cotidiano, construído coletivamente através de conceitos, explicações, crenças e ideias resultantes das experiências vivenciadas por indivíduos acerca de um determinado fenômeno (MOSCOVICI, 2007). Diversos autores contribuíram para o seu desenvolvimento e o crescente uso em pesquisas como aporte teórico, que se inicia nas bases sociológicas impulsionado por Emily Durkheim, e logo dissemina-se por diferentes áreas de investigação (SANTOS; DIAS, 2015).

Considera-se o teórico Serge Moscovici, como o autor de referência para a gênese da Teoria das Representações Sociais (TRS), à qual se destina a explicar e compreender uma realidade social, tornando familiar o que se encontra desconhecido ou distante. Outrossim, destaca seu importante papel ao pensar nos processos de formação de condutas e de orientação das comunicações sociais, e considerar o ser social, em um contexto individual e coletivo (MOSCOVICI, 2007).

Nesse sentido, a constituição de uma RS emerge de dois processos cognitivos-sociais, a ancoragem e a objetivação. Como mencionado, o processo de tornar familiar um objeto até então desconhecido, é denominado de ancoragem, ou seja, a partir das experiencias vividas por um grupo social é capaz de gerar uma rede de significados a um dado objeto. A objetivação carrega consigo a referência em materializar o que é abstrato, transformando o sentido em algo concreto (MOSCOVICI, 2007).

Esses dois processos se complementam e evidenciam a relação de três elementos cruciais no campo consensual das representações sociais que

continuamente são construídas e reconstruídas no cotidiano, por meio das interações sociais - o conteúdo, o objeto e o sujeito (MOSCOVICI, 2007).

No Brasil, os estudos com representações sociais se disseminam a partir da década de 1970, período em que os brasileiros tiveram a oportunidade de cursar módulos disciplinares e desenvolver pesquisas com grandes referenciais como Serge Moscovici e Denise Jodelet, e assim contribuir com a difusão da TRS e sua trajetória no território brasileiro. A exemplo de importantes eventos científicos que contribuíram para este fim, consistiu na Jornada Internacional sobre Representações Sociais: Teoria e Campos de Aplicação, realizada na Universidade Federal do Rio Grande do Norte em Natal no ano de 1998, onde apresentou-se nesse evento, um resumo das publicações científicas referentes aos últimos dez anos no campo das RS (SÁ; ARRUDA, 2000).

A base conceitual de sua teoria fundamentada na Psicologia Social, procura relacionar interações sociais, processos simbólicos (valores, cultura, modelos) e condutas, a partir do senso comum emergido pelas experiências de vida compartilhadas, bem como, busca a natureza social do conhecimento científico (MOSCOVICI, 2007; TRIANI; BIZERRA; NOVIKOFF, 2017).

As RS se apresentam como uma maneira de interpretar e pensar a realidade cotidiana, uma forma de conhecimento da atividade mental desenvolvida pelos indivíduos e pelos grupos para fixar suas posições em relação a situações, eventos, objetos e comunicações que lhes concernem (MOSCOVICI, 2007).

Dentre os desdobramentos das bases conceituais de sua teoria, Moscovici e outros autores**,** tais como, Jodelet (2009), Doise (2002), Abric (2002), destinaram seu olhar para o estudo do universo das representações sociais, em que favoreceu a possibilidade de refleti-las por meio de diferentes abordagens. Uma delas, na concepção de Denise Jodelet, caracteriza-se pela vertente antropológica-cultural, ao visualizar as RS de forma semelhante à Moscovici, mas, com um aprofundamento na reflexão sobre os elementos que apresentam aspectos sociais e organizacionais sob uma ótica igualitária a grupos, pessoas ou classes (JODELET, 2009).

Outra abordagem na RS, é a societal de Willem Doise (2002), que destaca os aspectos das representações sociais que valorizam o sujeito em sua individualidade e ao mesmo tempo, no cenário coletivo, perpassando pela interação do campo psicológico e sociológico (DOISE, 2002; ALMEIDA, 2009).

As RS ainda são vistas sob a perspectiva de Mary Jane Spink (1995), no qual as visualiza como uma abordagem de múltiplas vertentes, e conceitualiza-as como formas de conhecimento prático, que se estreita a corrente do conhecimento do senso comum.

Além dos autores mencionados, Jean Claude Abric em sua vertente contribui com o arcabouço da grande Teoria das Representações Sociais, ao visualizar as RS, como um conjunto de sentidos que se estruturam e se mostram mais saliente com elevada importância em detrimento de outros, o que deu fundamento a Teoria do Núcleo Central (TNC) (ABRIC, 2002).

4.2 JEAN CLAUDE ABRIC E A TEORIA DO NÚCLEO DO CENTRAL (TNC)

A vertente estrutural das RS proposta por Jean Abric, acredita ser o sujeito quem constitui a RS de um objeto, a reconstrói em seu sistema cognitivo e adéqua-a aos valores ideológicos que carrega consigo. Esse processo de apreensão se integra aos aspectos normativos do objeto, as experiências anteriormente vivenciadas dos sujeitos e as normas e valores de uma população (ABRIC, 2005).

A abordagem estrutural, centrada nos processos cognitivos das representações sociais, visa estudar a influência de fatores sociais nos processos de pensamento por meio da identificação e caracterização de estruturas de relações existentes no fenômeno a ser estudado, onde essas representações possuem uma organização e que apresentam duas características que se dizem contraditórias. Assim, as representações podem ser ao mesmo tempo estáveis e móveis, rígidas e flexíveis, bem como, consensuais, no entanto, marcadas por fortes diferenças individuais (ABRIC, 2005).

Nesse sentido, por mais que se constitua de uma entidade única, a organização das representações sociais é composta por dois sistemas onde apresentam funções específicas, mas complementar um ao outro e que apresentam características aparentemente contraditórias, pois para Abric, elas se mostram ao mesmo tempo estáveis e móveis, rígidas e flexíveis, bem como, consensuais, mas também marcadas por suas individualidades (ABRIC, 2005; SÁ, 2015).

Essa organização estrutural deu fundamento para a teoria de Abric, conhecida como Teoria do Núcleo Central (TNC). A TNC postula que a ideia essencial das representações sociais se dá de maneira estruturada, em que estas se organizam em

torno de um núcleo central, que determina ao mesmo tempo sua significação e organização. Esse núcleo concentra os aspectos que revelam significados básicos da representação marcado pela memória coletiva, são extremamente estáveis, determinam a homogeneidade de um grupo social, e apenas se modificam diante de uma alteração de grande porte no contexto social do grupo (ABRIC, 2005).

Também se constitui da presença de um sistema periférico que por sua vez se caracteriza como flexíveis e concretos, embora, apresentem aspecto móvel e evolutivo diante da resistência que os elementos componentes do núcleo central se apresentam. Esses elementos que irão constituir o núcleo central e o sistema periférico, se relacionam entre si, formando uma totalidade que dará sentido a um objeto do cotidiano. Estes elementos distribuídos entre essas duas zonas constituirão o quadro de quatro casas que a TNC propõe (ABRIC, 2005; SÁ, 2015).

No sistema periférico, a primeira periferia localizada no quadrante superior direito, frequentemente em função de sua importância, contempla os elementos que reforçam os elementos centrais. Os elementos que compõem a segunda periferia − quadrante inferior direito − é constituída por palavras menos prontamente evocadas e de menor frequência, apresentam menor significado ou importância para o grupo estudado. No conjunto de palavras que compõe a zona de contraste – quadrante inferior esquerdo –, encontram-se os elementos que obtiveram baixa frequência e baixa ordem média de evocação, mas que são considerados importantes para o grupo investigado (ABRIC, 2003).

O sistema periférico se vincula à realidade cotidiana, abarca os elementos de transição, e é responsável pela atualização do núcleo central. Essa dinamicidade promove a transformação da realidade social, além de contribuir para a modificação de comportamentos, condutas e ações em diversos contextos diferentes, como na área da saúde (ABRIC, 2003). É possível identificar elementos de caráter normativo, aqueles nos quais traduzem julgamentos, atitudes quanto ao fenômeno, e funcional, aqueles que se referem às práticas exercidas sobre o objeto (ABRIC, 2003).

Partindo da dúvida levantada por Abric (2003) ao refletir se os sujeitos questionados acerca de um determinado fenômeno dizem de fato o que pensam, emerge a assertiva de que nem sempre as representações serão acessadas de modo explícito ou expressadas verbalmente, o que Abric chamou de Zona Muda (ZM) das representações. Esse processo se dá pela presença de um contraste que pode haver entre o que as pessoas dizem e o que elas pensam e entre o que elas dizem e o que

realmente fazem na prática, o que coloca em risco a veracidade das representações apreendidas diante do contexto social.

A hipótese das RS da ZM se reflete em significados impregnados por valores sociais reconhecidos e comum entre os atores sociais e que colocam em xeque valores morais ou normas valorizados pela coletividade. A ZM portanto, concentra os elementos que tem caráter contra-normativo, que normalmente não são expressados em condições normais de produção e se encontram mascarados ou escondidos nessa zona (ABRIC, 2005; MENIN, 2006).

Uma reflexão é levantada ao pensar nas representações sociais e se esta corrobora com as práticas desenvolvidas pelos atores sociais (MAZZOTTI, 2002). Abric retrata, que o campo das representações sociais "deve ser encarado como condição para as práticas e as práticas como um agente de transformação das representações" (ABRIC, 2003).

Espera-se assim, que a vertente estrutural das RS, permita a visualização e análise dos aspectos considerados mais importantes levantados pelos profissionais de saúde diante do fenômeno em questão. E a partir da percepção dos mesmos, das vivências do cotidiano, identificar os fatores que possibilitarão a construção e reconstrução do significado. Essa análise se faz necessária diante da grande dificuldade que as pessoas em sorodiferença para o HIV, enfrentam para se expressarem em sua totalidade.

5 PERCURSO METODOLÓGICO

Este capítulo descreve o tipo de estudo e as técnicas e procedimentos de coleta de informações e de análise, que contemplam as etapas necessárias ao desenvolvimento desse estudo, assim como, os instrumentos necessários à sua realização.

5.1 TIPO DE ESTUDO

Trata-se de um estudo de campo do tipo descritivo, de abordagem qualitativa, fundamentado nos princípios da Teoria das Representações Sociais (TRS) na vertente estrutural com a Teoria do Núcleo Central (TNC) e Zona Muda (ZM) (ABRIC, 2003).

Numa investigação, a pesquisa qualitativa tem um caráter de aprofundamento de modo subjetivo, por meio da linguística, no intuito de melhor compreender um fenômeno em questão e responder a questionamentos particulares, em um cenário de significados, crenças, valores e atitudes dos sujeitos inseridos em um contexto (MINAYO, 2013; STAKE, 2011).

A sua parte descritiva, investiga os aspectos e características acerca da população, do fenômeno, e seus processos em determinados contextos, além do levantamento de opiniões, atitudes e crenças de indivíduos.

A TRS como referencial metodológico proposto por Abric (2003) configurada na TNC, busca, por meio da estrutura do quadro de quatro casas, apreender os elementos cognitivos compartilhados por uma população, normativos ou contra-normativos (zuna muda), que irão dá sentido ao objeto de estudo, e organizá-los em uma zona constituída pelo núcleo central e a zona periférica (ABRIC, 2003).

5.2 CENÁRIO DO ESTUDO

O estudo foi desenvolvido em três Unidades de Serviços de Assistência Especializada (SAE) em IST/HIV/aids, nos quais atuam no atendimento e acompanhamento de pessoas acometidas pelo HIV, e realizam atividades referentes ao Programa Nacional IST/Aids no Estado do Rio Grande do Norte.

Dos três serviços, dois deles estão localizados no município de Natal, um deles, gerido pela Secretaria Municipal de Saúde (SMS), responsável por atender a

demanda da capital do estado (SAE Natal); e o outro, gerido pela Secretaria Estadual de Saúde Pública (SESAP), localizado no Hospital Giselda Trigueiro em Natal (SAE HGT), destinado ao atendimento da população dos municípios do estado, destituídos desse tipo de serviço em seus municípios de origem. O terceiro SAE, está localizado no município de Parnamirim (SAE Parnamirim), região adjacente à capital do estado, sob gestão da SMS do referido município.

O estado do Rio Grande do Norte hoje, possui 14 unidades de SAEs, onde até dezembro de 2020 são responsáveis pelo atendimento médio de 7.406 pacientes em tratamento ativo para HIV, de acordo com a coordenação estadual do Programa IST HIV/aids do Estado. Dois desses serviços, são de responsabilidade da SESAP-RN, localizados nos dois maiores municípios do Estado, Natal e Mossoró, respectivamente, e os demais, localizados em outros municípios em desenvolvimento, situados geograficamente na região metropolitana da capital entre eles, Parnamirim, Macaíba, São Gonçalo do Amarante e São José de Mipibu, sob responsabilidade da esfera municipal, além de outros grandes municípios do sertão norte-riograndense como, Santa Cruz, São Paulo do Potengi, Caicó, e Pau-dos-Ferros.

As três unidades de SAE IST HIV/aids definidas como cenário do presente estudo, são responsáveis por atender o maior quantitativo de usuários da região metropolitana do Estado do Rio Grande do Norte. O SAE Natal, conta em média com um número em torno de 2.266 cadastros no programa de pessoas com diagnóstico de HIV em tratamento ativo, o SAE HGT possui outros aproximados 2.342 cadastros, e o SAE de Parnamirim um total de 503 cadastros, conforme a coordenação do programa de IST/HIV/aids do Estado.

Cada SAE conta com uma equipe multiprofissional mínima que atua diretamente juntos aos usuários, distribuídas entre médicos, assistente social, psicólogo, farmacêuticos, enfermeiros e técnicos de enfermagem. Atualmente, a equipe multiprofissional básica do SAE HGT possui 31 profissionais, do SAE Natal 13, e do SAE Parnamirim 9. Além disso, os três serviços contam com um coordenador local, e um coordenador responsável pelo programa a nível municipal e estadual.

5.3 PARTICIPANTES DA PESQUISA

Considerando o total de (59) profissionais distribuídos nos três SAE IST/HIV/aids, bem como, uma intencionalidade, foram incluídos 51 desses

profissionais das equipes multiprofissionais dos três SAE, selecionados de acordo com alguns critérios de inclusão, definidos como:

- Ser profissional da equipe de saúde multiprofissional básica dos SAE, composta por médico (infectologista ou clínico geral), assistentes sociais, psicólogos, farmacêuticos e equipe de enfermagem (enfermeiros e técnicos de enfermagem);
- Profissionais médicos ginecologistas da equipe especializada;
- Profissionais responsáveis pela gestão e coordenação local do SAE e do programa DST/HIV/Aids dos municípios de Natal e Parnamirim e da esfera estadual.

Como critérios de exclusão:

- Profissionais ausentes do serviço em período de férias, com licença ou atestado médico, e aposentados;
- Profissionais que não atenderem a três tentativas de contato;
- Profissionais que trabalham em mais de uma das unidades dos SAE selecionadas no estudo, e que já foram contemplados na coleta de dados.

Foram excluídos dois profissionais que estavam ausentes durante a realização do estudo, devido às férias e licença médica; e ainda um outro profissional, que não atendeu as três tentativas de contato; além de dois deles que trabalham, em mais de uma das unidades dos SAE selecionadas pelo estudo, já tendo sido contabilizado na coleta de dados por um dos SAE; Ao final, um profissional não quis participar do estudo, e outros 2 se aposentaram no período de realização das entrevistas, totalizando no total 8 profissionais que foram excluídos pelo estudo.

5.4 COLETA DOS DADOS

O procedimento de coleta dos dados ocorreu no período de outubro a dezembro de 2020, iniciou-se após o atendimento aos preceitos éticos de pesquisa, com a aprovação do Comitê de Ética em Pesquisa da Universidade Federal do Rio Grande do Norte (CEP-UFRN). À princípio, fez-se uma visita técnica aos SAE, pré-agendada junto a coordenação local dos serviços, para a apresentação formal do projeto a ser investigado pelo pesquisador, junto aos profissionais da equipe, de maneira que facilite a inserção do pesquisador junto ao serviço, e já fazer um consenso sobre o melhor dia e horário para realização da entrevistas.

A seguir, realizou-se um primeiro contato com os participantes, agendando as entrevistas, e o início da coleta de dados propriamente dita, através dos contatos com

os participantes, conforme os critérios de inclusão e exclusão previamente definidos, a fim de verificar na escala quais dias e horários o profissional estaria presente no serviço.

Após essa identificação junto aos profissionais, segue-se a segunda etapa da coleta, que consistiu no contato individual, com o intuito de verificar o interesse e aceite em participar da pesquisa.

Aos que sinalizaram e responderam positivamente, foram dadas orientações e esclarecimentos sobre a importância do acesso e conhecimento, ao Termo de Consentimento Livre e Esclarecido (TCLE), para leitura pausada e descritiva da pesquisa a ser realizada; ao aceitar os termos propostos, essa confirmação de participação deve ser selada por meio de assinatura no próprio documento. Àqueles sem desejo de participar, e/ou com indisponibilidade a entrevista nesse primeiro contato, fez-se um agendamento para a entrevista em uma data posterior combinada entre ambos (pesquisador x pesquisado).

Após a leitura do TCLE, e posterior assinatura, segue-se a etapa da entrevista, do tipo semiestruturada, norteada por um instrumento do tipo formulário, com um roteiro de questões, contendo em sua primeira parte informações pessoais e profissionais como, sexo, idade, tempo de atuação no serviço, capacitações e/ou pós-graduação na área de IST/HIV/aids. Além do roteiro de questões abertas, sobre informações relacionadas diretamente ao fenômeno em questão, que constituíram a estrutura das representações sociais, através da aplicação do Teste de Associação Livre de Palavras ou expressões (TALP) e a técnica de substituição e descontextualização - ZM.

O instrumento passou pelo processo de pré-testagem, com a finalidade de avaliar sua compreensão e entendimento quanto ao seu conteúdo. Foi testado com três profissionais do setor especializado em IST/HIV (uma médica, uma enfermeira e uma técnica de enfermagem), do Departamento de Atenção à Saúde do Servidor (DAS) da Universidade Federal do Rio Grande do Norte em Natal, o qual tem aproximação com o atendimento a usuários que vivem com HIV.

Vale ressaltar que o processo de testagem que antecedeu a coleta de dados, ocorreu no formato virtual por meio do envio do instrumento na plataforma digital *Word* aos participantes convidados, devido ao impacto gerado pela pandemia da COVID-19. Razão essa pela qual, a coordenação do setor tornou possível esse tipo de atendimento apenas neste formato. Foi enviado o instrumento completo e no final

deste, foi disponibilizado um espaço para registro de dúvidas/sugestões caso houvesse. Não houve sugestões de alterações no instrumento nessa fase.

A segunda parte do instrumento conteve as perguntas referentes a TALP, (APÊNDICE-A) que consiste em uma técnica que compreende aspectos que elicia a evocação de palavras a partir de um ou mais estímulos indutores (ABRIC, 2005; TRIGUEIRO *et al.*, 2016). A técnica se constitui de um termo indutor, padronizado da seguinte forma, "Se eu lhe digo "X", o que o senhor(a) pensa? Dê-me "X" palavras, que para você, caracterizam X" (COUTINHO; BÚ, 2017).

A TALP, compreende uma técnica amplamente utilizada em estudos com representações sociais, que compreende aspectos que elicia a evocação de palavras a partir de um ou mais estímulos indutores. Essa técnica oferece a possibilidade de apreender, de forma espontânea, as projeções mentais e os conteúdos implícitos ou latentes que podem ser ocultados nos conteúdos discursivos e reificados (ABRIC, 2005; TRIGUEIRO *et al.*, 2016).

Para a aplicação da técnica, foi solicitado que o participante dissesse as cinco primeiras palavras ou expressões que lhe vem à mente a partir do termo indutor *"pessoas em sorodiferença para o HIV*", e posteriormente foi solicitado que justificassem ou definissem com orações curtas cada termo escolhido.

Optou-se por considerar a ordem natural das evocações e não solicitar que os participantes hierarquizem os termos quanto sua importância, na intenção de evitar a interferência na avaliação da fluência natural das produções mentais e em consequência na organização dos elementos centrais e periféricos.

Logo após, solicitou-se ao participante que dissesse as cinco primeiras palavras que lhes vêm à mente através do seguinte termo indutor *"Se eu lhe digo, pessoas em sorodiferença para o HIV, o que o senhor (a) acha que os profissionais de saúde pensam?".*

Entende-se, que o uso dessa técnica de descontextualização em contextos, se mostra oportuna diante do arcabouço simbólico das pessoas em sorodiferença que carregam consigo, na maioria das vezes, um semblante marcado por estigma e preconceito gerados pela temática do HIV (OLIVEIRA; COSTA, 2007). A seguir, solicitou-se novamente ao participante que justificasse os termos.

As justificativas são utilizadas para fundamentar os termos que compõem o quadro de quatro casas, auxiliando na compreensão dos sentidos atribuídos aos

termos evocados. As evocações mencionadas e as definições/justificativas dadas pelos participantes, foram registradas pelo pesquisador no instrumento de coleta.

As entrevistas foram realizadas individualmente, em uma sala privada para o participante, livre de ruídos e de trânsito de pessoas de maneira que favorecesse o processo reflexivo que a técnica exige. É válido mencionar, que todas as entrevistas foram feitas no serviço de atuação do profissional, normalmente nas salas de atendimento do profissional, ou quando não, em uma sala disponibilizada pelo serviço e em acordo com a coordenação local.

O tempo de duração média das entrevistas, ficou entre 14 a 58 minutos e para a garantia do anonimato dos participantes, cada um foi nominado por um código representado pela letra “P”, de profissional, seguido de numeração ascendente, de acordo com a sequência das entrevistas, a exemplo, “P1”, “P2”, “P3”.

É importante mencionar, que devido ao impacto gerado pela pandemia da COVID-19, durante a etapa de coleta de dados, foram adotadas rigorosamente as normas de segurança, sobretudo durante as entrevistas, tais como, o distanciamento mínimo de um metro e meio entre o pesquisador e o participante, salas sem uso de ar condicionado, com portas e janelas abertas, sob o uso contínuo de máscara de proteção individual, afim de garantir a proteção do pesquisador em campo como dos participantes. Este protocolo de segurança já vinha sendo implementado na rotina dos serviços dos SAE.

5.5 ANÁLISE DOS DADOS

Os dados coletados foram inicialmente registrados na plataforma digital *Microsoft Office Word*, e para fins de organização, foram separados em três bancos diferentes. Um para contemplar o registro da caraterização dos participantes inerentes a primeira parte do instrumento, o segundo para as evocações oriundas da TALP e TALP ZM, e o terceiro com o dicionário semântico que compreende as definições das evocações pelos participantes.

Após a construção desses bancos, se deu a preparação das matrizes I e II (matriz I – TALP; matriz II: TALP ZM) para o processamento da análise prototípica - construção do quadro de quatro casas e análise de similitude. Como parte do processo de análise dos dados, as evocações foram submetidas ao processo de lematização e categorização para cada matriz, conforme demonstrado nos quadros a seguir.

A lematização (Quadro 1 e Quadro 2), consiste na análise do vocabulário, no qual, são verificados aspectos quanto ao número, gênero, tempo e redução para o alcance de uma única palavra, com intenção de agrupar os termos na origem e no radical da palavra (WACHELKE; WOLTER, 2011). Trata-se de um procedimento necessário, afim de evitar ambiguidades e divergências na fase de categorização.

Quadro 1 - Matriz I: Lematização das evocações TALP elaboradas pelos profissionais de saúde

Palavras de representação	Palavras evocadas
Sentimento	Sentimento<br>Sentimentos
Dificuldades	Dificuldade<br>Dificuldades
Positivo	Positiva<br>Positivo
Negativo	Negativa<br>Negativo
Convivência	Convívio<br>Convivência

Quadro 2 - Matriz II: Lematização das evocações TALP ZM elaboradas pelos profissionais de saúde

Palavras de representação	Palavras evocadas
Coragem	Corajoso<br>Coragem
Dificuldade	Dificuldade<br>Dificuldades
Estigma	Estigma<br>Estigmatização<br>Desestigmatização
Dúvidas	Dúvida<br>Dúvidas
Humanização	Humanidade<br>Humanização<br>Humano
Loucura	Loucura<br>Louco<br>Louca
Egoísmo	Egoísmo<br>Egocentrismo

A fase posterior à lematização se encontra a categorização das evocações (Quadro 3 e Quadro 4). Este processo se limita a organizar as palavras que constituem o mesmo sentido e significado, bem como demonstram haver relação em seus significados e conteúdos (WACHELKE; WOLTER, 2011; CAMARGO; JUSTO, 2013).

Quadro 3 - Matriz I: Categorização das evocações TALP elaboradas pelos profissionais de saúde

Palavras de representação	Palavras evocadas

Desconhecimento	Desinformação<br>Falta de informação<br>Desconhecimento<br>Conhecimento<br>Orientação<br>Informação
Comportamento sexual	Comportamento sexual<br>Sexualidade<br>Sexo desprotegido<br>Sexo<br>Relação sexual
Preconceito	Quebrar preconceito<br>Preconceito<br>Sem preconceito<br>Rejeição<br>Frustração
Família	Planejamento familiar<br>Família
Tratamento	Adesão ao tratamento<br>Tratamento<br>Remédio<br>Controle da doença<br>Esperança<br>Positivismo<br>Indetectável<br>Carga viral indetectável
Negativo	Negativo<br>Parceira negativa<br>Testagem regular<br>Acompanhamento<br>Vínculo
Medo	Retraídos<br>Medo<br>Ansiedade<br>Insegurança<br>Nervosismo
Dificuldades	Dificuldades<br>Obstáculos<br>Desafio<br>Conflitos
Prevenção	Proteção<br>Combinada<br>Prevenção<br>Segurança<br>PEP<br>PREP<br>Camisinha<br>Preservativo
Dúvidas	Dúvidas<br>Curiosidade<br>Até quando<br>Incógnita
Amor	Amor<br>Afeto<br>Carinho<br>Sentimento<br>Paixão
Ética	Sigilo<br>Ética
Risco	Risco

	Exposição<br>Irresponsabilidade<br>Vulnerabilidade<br>Transmissão
Aceitação	Em comum<br>Consenso<br>Comum acordo<br>Concordância<br>Aceitação
Parceria	Companheirismo<br>Cumplicidade<br>Parceria<br>União<br>Harmonia<br>Amizade<br>Abertura<br>Liberdade<br>Casal<br>Convivência<br>Homem e mulher<br>Relacionamento<br>Confiança
Lealdade	Sinceridade<br>Verdade<br>Lealdade
Compreensão	Entendimento<br>Compreensão
Acolhimento	Comunicação<br>Diálogo<br>Saber ouvir<br>Escuta<br>Acolhimento
Cuidado	Atenção<br>Cuidado<br>Humanização<br>Aconselhar
Vida	Vida<br>Qualidade<br>Saúde
Responsabilidade	Responsabilidade<br>Compromisso
Coragem	Admiração<br>Coragem

Quadro 4 - Matriz II: Categorização das evocações TALP ZM elaboradas pelos profissionais de saúde

**Palavras de representação**	**Palavras evocadas**
Trabalho em equipe	Equipe<br>Trabalho em equipe
Vida normal	Normalidade<br>Vida normal<br>Possibilidade de vida normal<br>Possibilidade<br>Família<br>Pessoas normais
Naturalização	Mais prevalente<br>Naturalização

Desconhecimento	Desinformação<br>Desconhecimento<br>Falta de conhecimento<br>Ignorância<br>Despreparo<br>Preparo<br>Indiferença<br>Conhecimento<br>Conhecimento técnico<br>Nível intelectual<br>Informação<br>Orientação<br>Entendimento
Preconceito	Preconceito<br>Sem preconceito<br>Pré-julgamento<br>Julgamento<br>Estigma<br>Repudia<br>Desconfiança<br>Tabu<br>Rejeição social<br>Exclusão social<br>Descriminação
Exames	Exames<br>Exames de rotina<br>Teste rápido para HIV<br>Repetir teste<br>Indetectável
Assistência	Palestra<br>Assistência<br>Atenção<br>Cuidado<br>Atendimento<br>Projeto<br>Conduta<br>Resolutividade<br>Acompanhamento<br>Disposição<br>Aceitação<br>Compromisso<br>Diálogo<br>Humanização<br>Dignidade
Tratamento	Tratamento<br>Adesão<br>Persistência<br>Qualidade de vida<br>Dedicação<br>Resultado
Amor	Amor<br>Se gostar
Parceria	União<br>Parceria<br>Solidez<br>Apoio<br>Comunhão<br>Respeito<br>Confiança<br>Cumplicidade

Verdade	Verdade<br>Sinceridade
Promiscuidade	Traição<br>Infidelidade<br>Promiscuidade
Opiniões diversas	Opiniões diversas<br>Discordam
Impossibilidade	Impossibilidade<br>Não se relacionaria<br>Não existe
Loucura	Loucura<br>Absurdo<br>Inexplicável<br>Sem noção<br>Desiquilibrado<br>Susto
Risco	Perigo<br>Risco<br>Vulnerabilidade<br>Exposição<br>Irresponsabilidade
Dificuldade	Dificuldade<br>Desafio<br>Limitações<br>Conflitos<br>Obstáculos
Coragem	Admirável<br>Coragem
Dúvidas	Curiosidade<br>Dúvidas
Submisso	Submisso<br>Dependência financeira
Medo	Medo<br>Contaminar-se<br>Insegurança
Ética	Ética<br>Sigilo
Capacitação	Capacitação<br>Segurança
Acolhimento	Acolhimento<br>Saber ouvir<br>Empatia<br>Deixá-los à vontade<br>Solidariedade<br>Amizade
Prevenção	PREP<br>Prevenção<br>Preservativo
Egoísmo	Egoísmo<br>Falta de empatia
Pena	Fardo<br>Pena<br>Sentença

Após a lematização e categorização, as evocações foram transcritas para uma planilha contendo informações na plataforma digital *LibreOffice* para posteriormente serem processados com o auxílio do software *Interface de R pourlesAnalyses*

Multidimensionelles de Textes et de Questionaires (IRAMUTEQ), versão 7 alpha 2. Este, consiste em um software com acesso aberto e gratuito desenvolvido por Pierre Ratinau que viabiliza diversos processos analíticos direcionados para pesquisas qualitativas em que sua operacionalização permite diferentes formas de sistematizar os dados a partir de palavras/frases, utilizando o cruzamento de parâmetros como cálculo de frequência de palavras e da ordem média que essas palavras aparecem (CAMARGO; JUSTO, 2013; SOUZA *et al*., 2018).

É bastante utilizado em pesquisas de representações sociais, e oferece a possibilidade de compreender a lexicografia básica e as multivariadas, por meio da classificação hierárquica descendente, análise prototípica e análise de similitude (CAMARGO; JUSTO, 2013; SOUZA *et al*., 2018). Nesse estudo, foram utilizadas as análises prototípica e de similitude para contribuir com a identificação e entendimento da estrutura das RS atribuídas pelos profissionais de saúde.

A análise dos dados relacionados aos aspectos de caracterização dos participantes, do tipo descritiva a partir do levantamento e organização das informações obtidas, está apresentada por meio de frequências relativa e absoluta, com auxílio do software *Microsoft Office Excel® v.2016*.

5.5.1 Análise Prototípica - Quadro de Quatro Casas

A análise prototípica baseia-se na avaliação das saliências dos elementos representacionais por meio da quantidade de vezes que o termo é citado e a ordem que o mesmo aparece, organizado em um quadro de quatro casas (WACHELKE; WOLTER, 2011). Seu intuito consiste em apresentar um padrão de organização baseado nas evocações sinalizadas (WACHELKE; WOLTER, 2011). É utilizado com frequência, em estudos que apresentam conteúdos sob forma de palavras ou expressões emitidas por participantes ao lerem ou ouvirem um determinado termo indutor (FLAMENT; ROUQUETTE, 2003).

Após a etapa do tratamento dos dados (lematização e categorização), segue-se a etapa de processamento das informações, por meio do cálculo da frequência da ocorrência das palavras e a Ordem Média de Evocações (OME). Esta consiste na posição média em que a palavra apareceu na classificação de ordem das evocações, onde o valor 1 foi atribuído a primeira resposta, 2 a segunda resposta e assim sucessivamente, mostrando quais termos foi proferido com maior rapidez

(WACHELKE; WOLTER, 2011). Dessa forma, os pontos de cortes são estabelecidos para a composição dos quadrantes, a partir do emprego da mediana nas palavras evocadas.

Nas cinco palavras evocadas das duas matrizes, o ponto de corte foi três, de acordo com a OME. E para a definição do ponto de corte para o eixo frequência, foi seguidas as orientações propostas por Verges (1991), onde foi feito o cálculo da frequência média dentre os termos pelo próprio software, após a exclusão das evocações com baixas frequências (definidas as evocações com frequências menores que três).

Assim, as palavras com OME ≤ 2 foram classificadas como baixa OME e ≥ 3 incluídas como alta, as quais foram selecionadas para presente análise (WACHELKE; WOLTER, 2011). A constituição dos valores para composição do quadro e respectivos componentes ‐ núcleo central e de elementos periféricos ‐ foram calculadas automaticamente pelo software IRAMUTEQ: frequência intermediária: 9.96; e OME: 3.06 para a matriz I e, frequência intermediária: 9.95; e OME: 2.95 para a matriz II (CAMARGO; JUSTO, 2013).

A posteriori, por meio do cruzamento entre as duas coordenadas (frequência e OME) e de acordo com seus valores, foram gerados quatro quadrantes constituintes do quadro de quatro casas, o núcleo central, a primeira periferia, a segunda periferia e a zona de contraste, cada um apresenta características específicas, conforme descrito no Quadro 5 (WACHELKE; WOLTER, 2011).

Quadro 5 - Quadro de quatro casas da TNC de Abric (2003)

**NÚCLEO CENTRAL (quadrante superior esquerdo)**	**PRIMEIRA PERIFERIA (quadrante superior direito)**
Concentra os termos que possuírem alta frequência e baixa OME, ou seja, as respostas evocadas prontamente e por um grande número de participantes.	Remete a zona que contempla as respostas com alta frequência e alta OME, o que evidencia termos que se destacaram, mas que são elementos secundários.
**ZONA DE CONTRASTE (quadrante inferior esquerdo)**	**SEGUNDA PERIFERIA (quadrante inferior direito)**
Constitui elementos claramente periféricos que irão possuir baixa frequência, e baixa OME. São as evocações consideradas menos importantes para os sujeitos.	Compete os termos que serão menos frequentes, o que mostrará aspectos mais particulares da representação social e possuem alta OME.

5.5.2 Análise de Similitude

A análise de similitude objetiva identificar as correlações entre os termos obtidos no questionário da TALP e TALP ZM, por meio de um coeficiente de semelhança. Sua dinâmica permite a visualização da conexidade das palavras e suas relações dentro de cada quadrante, bem como entre os quadrantes variados do quadro de quatro casas (MENDES *et al.*, 2016; SOUZA *et al*., 2018).

As etapas de análise de similitude, resultam das evocações e define-se inicialmente o conjunto de categorias semânticas pelas quais se distribuem as palavras, em seguida esse conteúdo passa por uma análise de co-ocorrências, na qual são quantificadas as frequências de co-ocorrencia de cada categoria em relação a todas as outras (MENDES *et al.*, 2016).

Como resultado, obtém-se a construção de uma matriz de similitude para cada matriz (I e II), visualizada por meio de um gráfico, a “árvore máxima”, do qual emergem os índices de semelhança para cada par de categorias, indicando assim a força das ligações estabelecidas entre às mesmas. O intuito dessa análise, é de interpretar a relação de vizinhança entre os termos e a conotação assumida por cada termo, em função dos outros termos que a ele se ligam. Esse processo foi importante para confirmar ou questionar a hipótese da centralidade resultante do quadro de quatro casos.

5.6 ASPECTOS ÉTICOS E LEGAIS DA PESQUISA

O presente estudo respeitou as normas e diretrizes éticas configuradas pela Resolução de nº466 de 2012, do Conselho Nacional de Saúde (CNS), exigidas ao tratar de pesquisas envolvendo seres humanos, na qual preconiza que a pesquisa científica deve assegurar os direitos e deveres inerentes aos participantes da pesquisa e pesquisadores.

O estudo foi aprovado pelo Comitê de Ética em Pesquisa da Universidade Federal do Rio Grande do Norte (CEP-UFRN), conforme parecer número 4.005.590 e Certificado de Apresentação para Apreciação Ética (CAAE) nº: 30794020.6.0000.5537.

Os participantes receberam os esclarecimentos necessários e tiveram sua autonomia e identidade preservadas durante sua participação integral, garantida por meio do TCLE. Além disso, os riscos inerentes à pesquisa foram mínimos, uma vez

que não houve reclamações, desistências, ou notificações negativas por parte da coordenação dos serviços ao pesquisador.

6 APRESENTAÇÃO E ANÁLISE DOS RESULTADOS

Os resultados serão apresentados em duas seções, a saber: 1) A primeira seção descreve a caracterização dos participantes do estudo a partir da análise estatística das variáveis contidas no instrumento. 2) A segunda apresenta os resultados inerentes à entrevista semidirigida com aplicação da TALP, onde pode-se acessar a estrutura das representações sociais tidas pelos profissionais por meio do quadro de quatro casas oriundo da análise prototípica e no campo semântico através da definição dos termos evocados, e a árvore máxima por meio da análise de similitude.

6.1 CARACTERIZAÇÃO DOS PARTICIPANTES

Dos 51 participantes do estudo, 54,9% (n=28) são profissionais do Serviço de Assistência Especializada do Hospital Giselda Trigueiro (SAE/HGT), outros 25,4% (n=13) são profissionais do Serviço de Assistência Especializada do município de Natal (SAE/Natal) e, 19,6% (n=10) que são profissionais do Serviço de Assistência Especializada do município de Parnamirim (SAE/Parnamirim).

Os 51 participantes apresentam-se em sua maioria, do sexo feminino (n=42; 82,35%), com faixa etária predominante dos 41 anos aos 54 anos (n=23; 45,10%). Quanto ao nível de escolaridade, 78,43% (n=40) possuíam ensino superior e 21,57% (n=11) ensino técnico, e 41,18% (n=31) relataram possuir especialização ou capacitação específica para atuar no serviço. Do total de participantes, 11,76% (n=6) eram coordenadores dos serviços e programas responsáveis, e 88,24% (n=45) profissionais da equipe assistencial multiprofissional, destes, sendo 33,33% (n=15) médicos, 24,44% (n=11) técnicos de enfermagem, 15,55% (n=7) farmacêuticos, 13,33% (n=6) enfermeiros, 6,66% (n=3) assistente social e 6,66% (n=3) psicólogos.

Quanto ao tempo de formação dos profissionais, houve variação de 1 ano e 10 meses a 46 anos de profissão, com predomínio de 16 a 30 anos (n=21; 41,17%), assim como o tempo de serviço no SAE esteve no intervalo de 9 dias a 37 anos onde em sua maioria esteve no intervalo de 9 dias – 3 anos (n=26; 50,98%).

Tabela 1 - Caracterização dos participantes. Natal/RN, 2021.

Variáveis	N	%
**Sexo**		
Feminino	42	82,35%
Masculino	9	17,65%
**Faixa etária**		
28 – 40 anos	19	37,25%
41 – 54 anos	23	45,10%
> 55 anos	9	17,65%
**Nível de escolaridade**		
Nível médio/técnico	11	21,57%
Nível superior	40	78,43%
Especialização ou capacitação específica para atuar no serviço	31	41,18%
**Atuação profissional**		
Coordenadores dos serviços e programas	6	11,76%
Profissionais da equipe assistencial multiprofissional	45	88,24%
**Profissionais da equipe assistencial multiprofissional**		
Médico	15	33,33%
Assistente social	3	6,66%
Psicólogo	3	6,66%
Farmacêutico	7	15,55%
Enfermeiro	6	13,33%
Técnico de enfermagem	11	24,44%
**Tempo de formação na área**		
≥ 5 anos	6	11,76
6 – 15 anos	18	35,29
16 – 30 anos	21	41, 17
> 30 anos	6	11,76
**Tempo de serviço no SAE IST/HIV/aids**		
9 dias – 3 anos	26	50,98
4 – 10 anos	16	31,37
11 – 20 anos	5	9,80
> 20 anos	4	7,84

6.2 ENTREVISTA SEMIDIRIGIDA

Os resultados obtidos através da entrevista semiestruturada com os participantes, deram origem ao quadro de quatro casas que compete a análise prototípica, e a árvore máxima referente a análise de similitude. Para ambas as análises, a entrevista seguiu as recomendações propostas pela TALP, na qual, teve como termo indutor “pessoas em sorodiferença para o HIV”, tanto utilizada em caráter de normalidade que deu origem a matriz I, quanto em sua versão de descontextualização e substituição (TALP ZM) que originou a matriz II. As representações sociais no campo semântico de cada termo por meio da definição dada pelos participantes, são destacadas conforme a distribuição das evocações no quadro de quatro casas.

6.2.1 Processamento Prototípico – Quadro de quatro casas (Matriz I: Técnica de Associação Livre de Palavras)

Na análise prototípica da matriz I, o termo indutor: “*pessoas que vivem em sorodiferença para o HIV*” resultou em 255 evocações e 93 evocações distintas. Após lematização e categorização, o número de palavras diferentes evocadas pelos profissionais foram 47.

Excluindo-se as evocações com frequência inferior a três, resultou-se em um aproveitamento de 48,93% (n=23). Abaixo, o Quadro 6 evidencia o resultado da análise prototípica da matriz I identificada no quadro de quatro casas com sua provável composição.

Quadro 6 - Quadro de quatro casas – Matriz I, resultantes do termo indutor: “pessoas que vivem em sorodiferença para o HIV”

	**OME ≤ 3.06**			**OME > 3.06**		
		f	**OME**		***f***	**OME**
***f* ≥ 9.96**	Parceria	31	2.9	Prevenção	27	3.4
	Amor	19	2.6	Tratamento	12	4.3
	Medo	17	2.8	Desconhecimento	11	3.4
				Cuidado	11	3.3
		f	**OME**		***f***	**OME**
***f* < 9.96**	Aceitação	9	2.7	Dificuldades	8	3.4
	Acolhimento	8	2.4	Responsabilidade	7	3.4
	Risco	8	1.9	Compreensão	7	3.4
	Comportamento	7	2.6			
	sexual	7	2.9			
	Preconceito	6	2.7			
	Negativo	6	2.7	Vida	6	3.8
	Respeito	5	2.8	Coragem	6	3.2
	Dúvidas	3	2.7	Sigilo	5	3.8
	Família			Lealdade	3	3.3

Legenda: f: frequência; OME: Ordem Média de Evocações.
Fonte: Dados da pesquisa.

A partir do cruzamento das coordenadas frequência e OME, o quadrante superior esquerdo identificou como representações do núcleo central tidas por um elevado número de participantes e evocadas mais prontamente, com provável composição dos elementos semânticos “parceria”, “amor” e “medo”.

> Amor é um sentimento que não impõe condições. Você ama o outro da forma que ele é. Quando a pessoa ama, ela não impõe condição da doença. O amor surge naturalmente. (P47)
>
> Um tem que que ser parceiro do outro, confiar no outro, tem que ser muito companheiro, parceiro, amigo, pra aceitar essa doença. (P8)
>
> Existe o medo de perder a pessoa ao seu lado, medo de ver a pessoa sofrer, medo de adquirir o HIV. (P12)
>
> Vou poder viver feliz? Ter filhos? Tem remédios? Essas inseguranças levam ao medo por ser algo muito estigmatizado. (P40)

O quadrante superior direito teve sua provável composição centralizada nas evocações, “prevenção”, “tratamento”, “desconhecimento” e “cuidado”. Estes termos, portanto, representam a primeira periferia do quadro e se configuram como prováveis elementos secundários ao núcleo central por apresentarem alta frequência e alta OME.

> Quando seu parceiro tem uma condição de saúde comprometida eles tem esse cuidado. Um traz o cuidado pra ambos e percebemos esse envolvimento do parceiro negativo com o parceiro que é positivo nos serviços. (P36)
>
> Descontruir o medo e estigma, tranquilizar que o tratamento hoje tem controle de carga viral em três meses. Não vão deixar de viver por isso. Não é criar oba oba, mas não impede de construir seus sonhos e conviver com o HIV. (P40)
>
> Poder conhecer o que é, e como é transmitido, as dificuldades as formas de proteção como a PREP. Não ficar só no que me disseram, e não ficar refém disso e sim buscar conhecimento. Tem casais aqui que são sorodiscordantes, tem filho negativo, vejo que eles buscaram através do conhecimento esse “final” feliz. (P29)

No terceiro quadrante (quadrante inferior direito), considerado periferia próxima ou segunda periferia, os termos prováveis consistiram em “dificuldades”, “responsabilidade”, “compreensão”, “vida”, “coragem”, “sigilo” e “lealdade”. Esses termos representam aspectos mais particulares das construções cognitivas dos profissionais por terem sido mencionados por uma pequena quantidade de profissionais.

> A pessoa que é sorodiscordante ela própria não aceita, ela esconde, elas as vezes não é sincera pra contar seu estado sorológico para o parceiro. Vai se relacionar com o outro então tem que ser sincero. (P25)
>
> A compreensão é saber dentro das suas possibilidades educacionais, sociais e culturais os seus riscos diante da escolha de estar junto. (P38)
>
> Responsabilidade mútua, tanto do paciente portador para fazer o tratamento e chegar à carga viral indetectável, como para o soronegativo, de buscar por exemplo a PREP, para assim manter uma relação segura. (P47)
>
> A limitação na relação sexual. Quer queira ou não o uso do preservativo é visto como uma limitação. (P39)

No tocante ao último quadrante (quadrante inferior esquerdo), denominado zona de contraste, se encontram os elementos claramente periféricos que possuíram baixa frequência, mas que foram considerados importantes por serem evocações ditas prontamente. A provável composição deu-se com os termos “aceitação”, “acolhimento”, “risco”, “comportamento sexual”, “preconceito”, “negativo”, “respeito”, “dúvidas” e “família”.

> Atendemos muitos casais assim, fazemos a escuta sensível e qualificada, eles chegam bastante sensíveis em relação a preconceitos e discriminações. Importante se colocar pra acolher em suas angustias, dúvidas e inseguranças. (P5)
>
> As dúvidas deles tem relação em saber quais os riscos de transmissão quando eles estão nesse tipo de relação. (P35)
>
> Quando penso em sexo eu imagino que as pessoas sorodiscordantes elas fazem e buscam no serviço a melhor forma de fazer o sexo. (P13)
>
> Acho que é possível um casal que é sorodiscordante constituir uma família, como casal, com filhos, independente. (P16)

6.2.2 Processamento Prototípico – Quadro de quatro casas (Matriz II: Modelo de substituição e descontextualização – Zona Muda)

Para a análise prototípica da matriz II, sob o formato da TALP ZM a partir do termo indutor: “pessoas que vivem em sorodiferença para o HIV”, resultou-se em 255 evocações e 156 evocações distintas. Após lematização e categorização, o número de palavras diferentes evocadas pelos profissionais foram 51. Excluindo-se as evocações com frequência inferior a três, resultou-se em aproveitamento de 43,13% (n=22). Abaixo, o Quadro 7 evidencia a análise prototípica por meio do quadro de quatro casas e sua provável composição.

Quadro 7 - Quadro de quatro casas – Matriz II, resultantes do termo indutor: “pessoas que vivem em sorodiferença para o HIV”

	OME ≤ 2.95			OME > 2.95		
		f	**OME**		***f***	**OME**
$f \geq 9.95$	Preconceito<br>Desconhecimento<br>Medo<br>Loucura<br>Amor	24<br>21<br>16<br>10<br>10	2.5<br>2.1<br>2.8<br>2.4<br>2.5	Assistência<br>Acolhimento<br>Parceria	28<br>13<br>13	3.5<br>3.2<br>3.5
		f	**OME**		***f***	**OME**
$f < 9.95$	Coragem<br>Dificuldade<br>Dúvidas<br>Vida_normal<br>Egoísmo<br>Impossibilidade	9<br>9<br>6<br>6<br>4<br>3	2.6<br>2.6<br>2.2<br>2.8<br>2.2<br>2.0	Prevenção<br>Risco<br>Promiscuidade<br>Pena<br>Tratamento<br>Exames<br>Ética<br>Capacitação	9<br>8<br>6<br>6<br>6<br>5<br>5<br>4	3.8<br>3.0<br>3.5<br>3.7<br>4.2<br>4.0<br>3.0<br>3.2

Legenda: f: frequência; OME: Ordem Média de Evocações.
Fonte: Dados da pesquisa

A provável composição do núcleo central da matriz II foi constituída pelos termos “preconceito” (termo mais evocado), seguido de “desconhecimento” (termo mais prontamente evocado), “medo”, “loucura” e “amor”.

> Tem que amar demais para poder ficar. (P34)
>
> O preconceito é o produto do perfil dos profissionais conservadores que na minha visão os profissionais tem. A maioria dos casais são homoafetivos, e mesmo nos heterossexuais, eles não veem com naturalidade essa pessoa que é saudável ser digna, se relacionar com alguém doente. (P30)
>
> Nesse caso com profissionais da saúde o preconceito é mais dolorido, principalmente do SAE, criam situações constrangedoras quando se trata de casais sorodiscordante. (P21)
>
> Os profissionais têm medo de se deparar com essa situação, principalmente diante do diagnóstico e não saber lidar com o casal. (P16)
>
> Existe o medo por parte dos profissionais de pegar (o HIV) a partir do paciente. Se hoje a gente vive a estigmatização com a covid-19, com o HIV foi muito pior e perdura até hoje. (P40)
>
> Há necessidade de mais qualificações, compreensão da rede, da interiorização. Há muita dificuldade e necessidade de trabalhar o conhecimento, principalmente relacionado a sexualidade. (P29)

No segundo quadrante, com a composição provável dos termos “assistência”, “acolhimento” e “parceria”, termos com frequências e ordem média de evocação elevadas.

> Precisamos cada vez mais melhorar o serviço no sentido de capacitação, estrutura física, medicamentos, para que a assistência seja a melhor possível. (P26)
>
> Vejo que os profissionais precisam buscar uma especialidade na questão de acompanhar esse tipo de problema, pra que eles sejam acompanhados por alguém que realmente entenda. (P14)
>
> Percebo que quando se trata de casal a aproximação tem q ser maior, o cuidado redobrado. A pessoa que não tem o vírus as vezes quer até se contaminar. E aí a gente precisa ver se esse casal conhece os métodos de prevenção e profilaxia. (P13)
>
> Vejo os profissionais comprometidos na forma de acolher e ajudar. (P10)

No que compete ao terceiro quadrante, ou seja, os elementos que apresentaram baixa frequência e alta OME, consistiram em “prevenção”, “risco”, “promiscuidade”, “pena”, “tratamento”, “exames”, “ética” e “capacitação”.

> A primeira coisa que se faz diante de uma sorodiscordância é a prevenção, não tem como não perpassar por ela, tanto para o parceiro positivo como para o parceiro negativo. (P17)
>
> Quando fazem o tratamento direitinho a carga viral zera. Hoje é outra realidade. Esses casais vivem como se tivessem uma vida normal. Hoje se sabe quando zera a carga viral, ela praticamente não transmite. (P24)
>
> Vejo nos profissionais um compromisso de agir sempre corretamente. O trabalho em equipe funciona, graças a ética que existe. (P10)
>
> Quando se fala se fala que está com um paciente soropositivo, alguns profissionais já pensam em vários parceiros, anda com bandidos, aquela coisa do início da epidemia que ainda persiste. Tenho paciente que contraiu HIV do único parceiro que teve na vida. (P19)
>
> Pena é um sentimento terrível, eles não os veem como pessoas que querem lutar, acham que estão fazendo caridade. Não olham como seres humanos, olham com pena, com piedade. (P12)
>
> A capacitação é fundamental para saber conduzir a situação desses casais. (P7)

No tocante ao quarto quadrante, a zona muda revelou como elementos de contraste os termos, “coragem”, “dificuldade”, “dúvidas”, “vida normal”, “egoísmo” e “impossibilidade”, o que apesar de serem considerados termos menos importantes evocados pelos profissionais, foram elementos prontamente evocados.

> Coragem é um sentimento que os profissionais imaginam, um reflexo do que eles pensam, quando estão diante dessas pessoas que vivem em sorodiscordância, principalmente do parceiro negativo. (P36)
>
> Os profissionais realmente veem essas pessoas com muitas dúvidas, as formas de contágios, como conviver com isso. Questão de engravidar... (P51)
>
> Muitos profissionais já tem o conhecimento que isso é uma possibilidade, diante do avanço do tratamento e outras formas de prevenção. (P16)
>
> Eles (os profissionais) se concentram neles mesmo porque pensam “eu não vim pra atender esse tipo de paciente, eu não preciso atendê-los”. (P12)

6.2.3 Análise de similitude – Matriz I Técnica de Associação Livre de Palavras

Após os procedimentos de lematização e categorização das evocações, a análise de similitude revelou as coocorrências entre as palavras e o seu resultado, onde evidenciou as indicações de conexidade entre as evocações. As arestas representam a força da conexão entre os valores da associação das palavras e os vértices (círculos), a formação de núcleos semânticos de significados, sendo proporcional à frequência das palavras evocadas. As cores representadas, ilustram a formação de comunidades, nas quais representam a aproximação dos termos que possuem relação maior entre si.

Através dessa análise, foi possível perceber como os profissionais de saúde relacionam as várias palavras para descreverem as suas RS. Foram incluídas para a análise de similitude da matriz I e II assim como para a análise prototípica, as evocações do banco com frequência a partir de três.

Conforme a Figura 1 para a análise de similitude da matriz I, verificou-se com base na sua estrutura, que os maiores eixos organizadores das representações tidas, são revelados pelos núcleos semânticos “parceria”, “prevenção”, “amor” e “medo”, o que confirmou a centralidade da zona central do quadro de quatro casas da matriz I.

Em sua comunidade, o núcleo parceria, se relaciona com os elementos periféricos “amor” (9), “aceitação” (3), “comportamento sexual” (2) e “lealdade” (2). O núcleo “amor” (9), representado em sua comunidade pela relação com os elementos periféricos “medo” (5), “dificuldades” (5), compreensão (4), responsabilidade (3), coragem (3), vida (2). O medo forma uma comunidade com os termos dúvidas (2), preconceito (2) e tratamento (4).

E por último, uma outra aresta que se destacou, foi a relação do termo tratamento com o núcleo semântico “prevenção” (6), na qual como elementos periféricos, destacam-se os termos, negativo (4), risco (2), família (2) e cuidado (5).

Nessa mesma comunidade, o elemento cuidado em seu núcleo, apresentou relação com as evocações desconhecimento (4), respeito (3) e acolhimento (3).

Figura 1 - Análise de similitude das evocações (TALP)

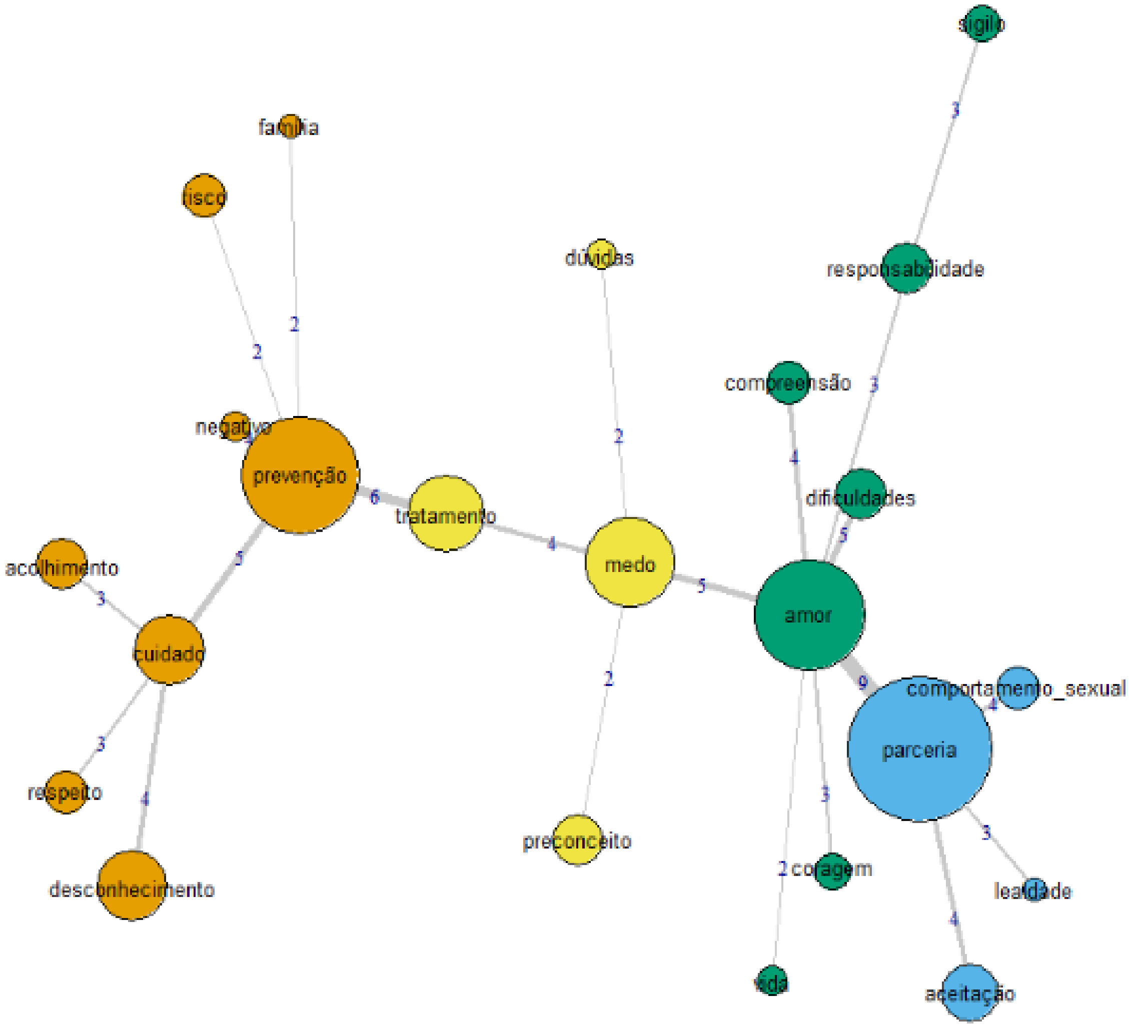

6.2.4 Análise de similitude – Matriz II Modelo de substituição e descontextualização (Zona Muda)

A Figura 2 contempla a estrutura da análise de similitude da matriz II. Verifica-se que os dois maiores eixos organizadores das representações tidas conforme o pensamento "dos outros profissionais de saúde", são os termos "assistência" e "preconceito", nos quais foram evocações com maior destaque pelos profissionais quanto ao termo indutor da TALP ZM e que apresentaram maior coocorrencia.

O núcleo semântico "assistência" em sua comunidade representacional apresenta em sua ramificação forte relação com os termos "acolhimento" (5),

“desconhecimento” (8), “ética” (5), “dificuldade” (4), “capacitação” (4) e “tratamento” (2).

O núcleo representacional do elemento “desconhecimento” possui relação com os dois maiores polos da árvore, assistência como já mencionado, e preconceito (8). O elemento preconceito por sua vez, traz em sua comunidade a relação com os termos “medo” (7), “promiscuidade” (4), “pena” (4), “egoísmo” (3), “parceria” (3), “risco” (3) e “impossibilidade” (1).

Parceria em seu núcleo semântico, ao mesmo tempo que apresenta relação com o termo preconceito, evidencia forte relação com “amor” (6), que este traz consigo as caracterizações da “loucura” (3) e “coragem” (3).

Figura 2 - Análise de similitude das evocações (TALP ZM)

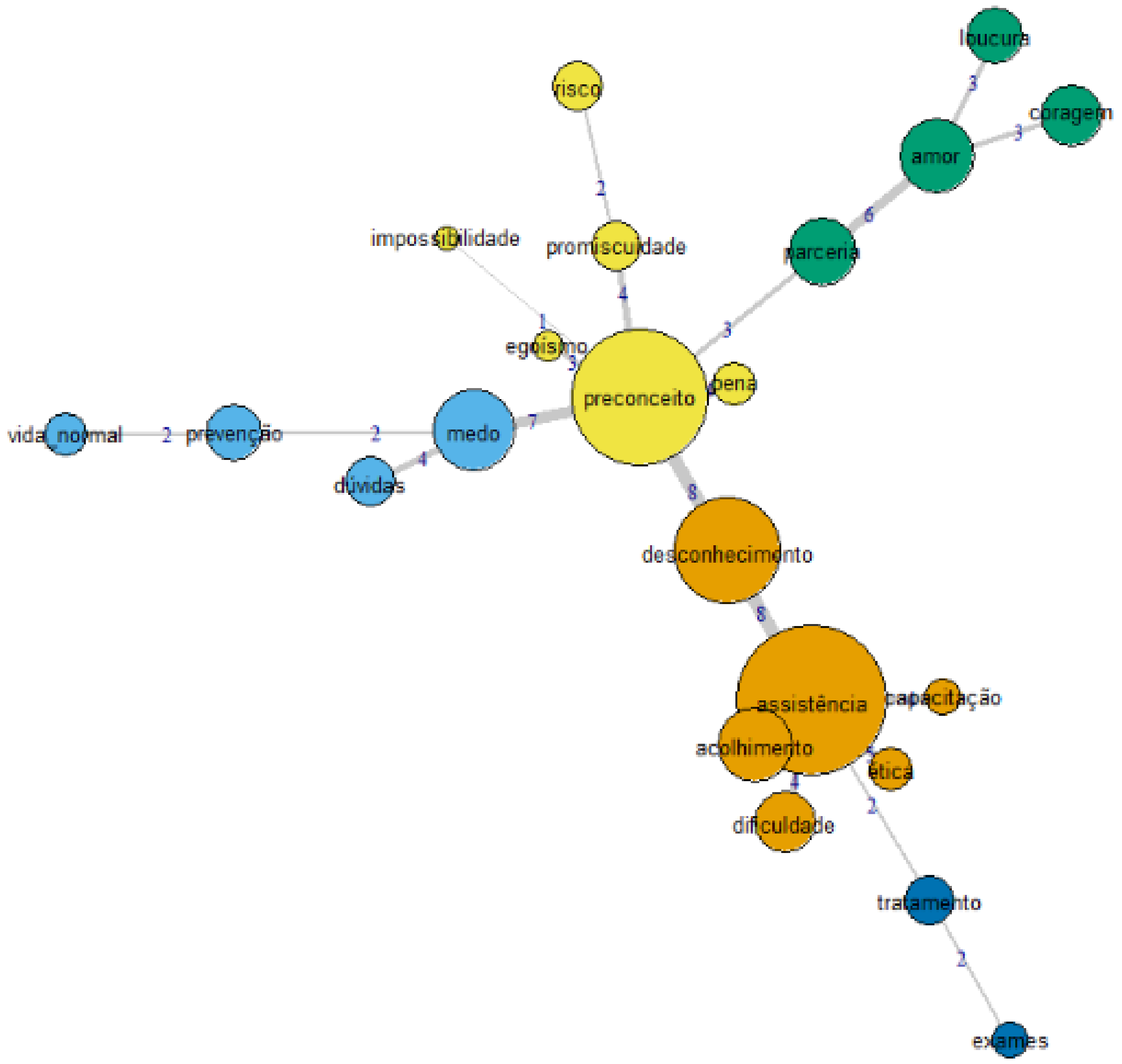

7 DISCUSSÃO

A matriz I traz em seu núcleo central o que Abric (2003), revela ser a identidade e constância do grupo segundo a memória coletiva e o caráter consensual dos sujeitos acerca de um objeto no contexto das representações sociais. Ao que compete a matriz I vê-se que, para os profissionais, a hipótese do núcleo central é vista como a determinação de uma parceria (termo mais evocado) entre as pessoas sorodiferentes para o HIV. Num sentido, de uma união regada pela dimensão sentimental do amor, visto como algo que supera tudo, tornando-se o ponto de partida para as possibilidades dessa relação.

A literatura, corrobora com esses sentidos e revela que os aspectos sentimentais se configuram como um elo importante em parcerias dessa natureza, e que essa dimensão subjetiva precisa ser valorizada pelos profissionais de saúde durante o acompanhamento desses parceiros, diante das interferências que os sentimentos e desejos possam gerar no processo saúde-doença desses sujeitos (LAGO; MAKSUD; GONÇALVES, 2013). E a valorização dessa dimensão subjetiva, pôde ser confirmada na análise de similitude desse estudo, onde ambas as matrizes trazem parceria e amor com uma forte relação entre si.

Essa representação exterioriza significados positivos diante de evidencias trazidas por parceiros com HIV em relações sorodiferentes, onde apontam fragilidades na atenção dos profissionais, por eles ainda não reconhecerem os sentimentos de cunho afetivo/sexual nas parcerias sorodiferentes, tornando-se talvez, um empecilho para a compreensão numa dimensão psicológica, emocional ou afetiva desses usuários (SILVA *et al*., 2018).

Observa-se, que a centralidade dessa representação da dimensão subjetiva, reforça perspectivas as quais indicam, que o envolvimento com o sexo, apesar de sua relevância, cede ao protagonismo de sentimentos, como o amor, o cuidado, e mesmo a convivência com o parceiro (MAKSUD, 2012).

Outra provável representação do núcleo central desse estudo e, um dos grandes desafios vivenciados pelos parceiros sorodiferentes para o HIV, e elencada pelos profissionais de saúde, é o medo. Viu-se que mesmo diante do sentido do amor como algo que tudo supera, essas pessoas não estão isentas de sentirem medo da possível contaminação do parceiro, ou, de não serem aceitas, e mesmo, não poderem

ter filhos. Estudos evidenciam de fato a presença do medo nesse contexto, sobretudo, se vivenciado pelo parceiro soronegativo (KENYON *et al*., 2015; FELIX *et al*., 2019).

Em contrapartida, a literatura também revela que o medo e muitas vezes o preconceito são realidades que estão sendo ultrapassados, pois ter um parceiro sorodiferente para o HIV se associa com indicadores positivos, como a adesão ao tratamento, melhor suporte social e melhor qualidade de vida (ODOYO *et al.*, 2019).

Na sequência, a análise do segundo quadrante da matriz I, apresenta significados que reforçam e dão sustentabilidade às crenças encontradas no núcleo central, através de termos de alta frequência que se inclinam para o núcleo central (ABRIC, 2003), através dos elementos prevenção (termo mais evocado), tratamento, cuidado e desconhecimento.

A prevenção é vista pelos profissionais como uma adoção dos métodos cujo objetivo, é o de proteger o parceiro soronegativo da contaminação do vírus, e constituir-se como principal estratégia de cuidado de ambos nessa parceria. Em geral, é feita através do uso do preservativo, das profilaxias PREP e PEP, e da adesão ao TARV do parceiro que vive com HIV.

Em outras palavras, entende-se que essa provável representação dos profissionais, é baseada no amor e quem ama, cuida do parceiro e busca na constância do dia-a-dia, a prevenção, afim de minimizar os medos e viver uma relação afetiva e sexual mais segura e com qualidade de vida, hipótese essa, confirmada na centralidade e ligação desses elementos na análise de similitude da matriz I (figura 1) (SAID; SEIDL, 2015).

Outra representação acessada desse segundo quadrante da matriz I, e que é provável trazer sentido ao elemento medo do núcleo central, é o desconhecimento, uma vez que para os profissionais, em se tratando do HIV desconhecem em sua totalidade as formas de transmissão e prevenção, e que portanto, leva a uma convivência cotidiana de medo.

Essa possível representação é reforçada pela análise de similitude da matriz I (figura 1), ao ver a relação de conexidade de duas comunidades representadas pelo núcleo central prevenção e tratamento, e em suas periferias ligações com os termos negativo, cuidado, desconhecimento, e com o elemento medo, respectivamente.

O terceiro quadrante da matriz I, revela prováveis representações consideradas menos importantes para os profissionais de saúde, haja vista, encontrar-se diante da baixa frequência dos elementos, e a não prontidão em evocá-los. Mesmo sendo

consideradas pelos profissionais como pessoas de coragem por viver uma relação sorodiferente, as representações também se revelam nas dificuldades imbricadas da sexualidade, vivenciadas pelos parceiros, vistas como sentimentos de culpabilização, ao desconhecimento e a resistência no uso dos métodos preventivos como o preservativo, requerendo dos parceiros mais envolvimento, conhecimento e consciência quanto aos riscos de uma relação sexual desprotegida.

Estudo de Fonte Boa *et al.* (2018), sobre as representações sociais de mulheres que vivem com HIV, reforçam a dificuldade para vivenciar a sexualidade entre os parceiros, mais precisamente o ato sexual, atribuindo esta circunstância, como regrada e dotada de prescrições normativas impostas pelos profissionais de saúde, passando a vê a relação sexual de forma limitada e pouco prazerosa.

Uma abordagem ampliada acerca dos métodos de prevenção para o HIV, é apontada como estratégia fundamental pelos profissionais de saúde. Nela, o uso do preservativo não deve se configurar como único método apresentado aos parceiros, dentro da diversidade que a prevenção combinada possibilita, pois cada parceria deve ser acolhida e avaliada conforme suas singularidades e dificuldades (REIS *et al*., 2019).

Nesse ínterim, outro sentido que emergiu foi a responsabilidade para com a saúde do(a) companheiro(a), bem como, a lealdade considerada indispensável nessa relação para que não haja sigilo, sobretudo quanto a sorologia de positividade para o HIV. Esses sentidos se configuram relevantes para que a vivência da sorodiferença proporcione qualidade de vida para ambos os parceiros.

A confirmação dessa provável representação se dá na análise de similitude onde os termos, dificuldades, responsabilidade, compreensão, sigilo, coragem e vida se relacionam com o núcleo central amor, atribuindo a ideia de completude a centralidade desse sentimento.

Quanto ao quadrante da zona de contraste, os elementos presentes demonstram uma relação de complementariedade junto à primeira periferia (segundo quadrante), conforme Abric (2003) revela em seus postulados, acerca das possibilidades de relação deste quadrante, ao trazer sentidos que remetem a convivência entre os parceiros, como a aceitação do parceiro soronegativo e o respeito necessário que a relação a dois requer.

Por sua vez, o elemento risco, mesmo apresentando uma frequência baixa, foi o termo mais prontamente evocado de todo o quadro, o que revela sua importância

nas representações dos profissionais de saúde, ao encarar o relacionamento entre pessoas sorodiferentes para o HIV, como uma relação de risco, não apenas pela possibilidade de haver a contaminação do parceiro soronegativo, mas de ser uma relação com riscos de sofrimento pela possibilidade do término da relação ou até mesmo de perda do parceiro.

Outro sentido a ser considerado, é o dilema do preconceito presente em cenários como família, trabalho, círculo de amigos e até mesmo entre os parceiros, talvez devido, a falta de conhecimento inerente ao contexto do HIV, ao associá-lo com o termo medo, conforme pode ser visto na análise de similitude, uma vez que, os parceiros podem sentir medo da exclusão social onde o preconceito é visto duplamente (ALBUQUERQUE; BATISTA; SALDANHA, 2018).

Por fim, ainda neste quadrante, os profissionais elencaram representações acerca das dúvidas presentes no cotidiano das pessoas que convivem com a sorodiferença à respeito dos métodos preventivos e da possibilidade de concepção do casal, representado pelo termo família, e mencionam a importância do acolhimento nos serviços dos parceiros com destaque para o parceiro soronegativo, representando um usuário do serviço que também precisa de acompanhamento e cuidados.

A necessidade de haver uma articulação de políticas de planejamento reprodutivo, fundamentais para os casais sorodiferentes vem sendo apontada na literatura como forma de suprir essa demanda de informações que parece ainda distante desses usuários (OLIVEIRA; ARAÚJO; ALVES, 2020; LANGENDORF; PADOIN; SUZA, 2020).

Além disso, existe a barreira do receio e da vergonha pela busca no serviço, com o objetivo de sanar as dúvidas sobre o desejo de ter filhos, é uma realidade dos parceiros, em virtude do medo de receber julgamentos e serem taxados como não merecedores da concepção (OLIVEIRA; ARAÚJO; ALVES, 2020).

Em estudo com profissionais de saúde, cujas representações sociais levantadas sobre o acolhimento de pessoas que vivem com HIV, mostrou que, o acolhimento entre parceiros sorodiferentes é uma estratégia a ser sempre priorizada, por compreender a necessidade de haver consenso e entendimento conceituais sobre o HIV, sobretudo do parceiro soronegativo, por normalmente referir mais dúvidas sobre os riscos de contaminação (SANTOS *et al*., 2019).

Conforme a Figura 1, a apresentação gráfica da árvore de similitude, indica que os termos da zona de contraste do quadro de quatro casas, aparecem na periferia da

árvore e se relacionam aos núcleos centrais, prevenção, parceria, medo e cuidado, os quais, comportam características que dão sentido aos elementos que representam a zona central.

Ao que se refere a zona muda que tem a função de validar os significados encontrados na normalidade ou revelar conteúdos latentes na descontextualização de papéis conforme revela Abric (2003), consiste em uma estratégia fundamental para o alcance das representações sociais dos profissionais, pois possibilitou acessar conteúdos encapsulados, os quais nesse estudo, não foram possíveis acessá-los na normalidade de papéis (ABRIC, 2003).

O contexto contra-normativo (matriz II) apresentado, reforça a representação da díade parceria e amor acessada na normalidade por meio da cristalização dos elementos amor presente também no núcleo central, e o elemento parceria presente na primeira periferia que também se caracteriza como um quadrante importante e que complementa os sentidos atribuídos ao núcleo central (ABRIC, 2003).

A análise de similitude com a função de revelar as prováveis relações que os termos estabelecem um com o outro, mostra a presença de ligação desses dois termos nas árvores das duas matrizes, o que reforça a possível cristalização dessa representação (MENDES *et al.*, 2016; SOUZA *et al*., 2018).

Portanto, a centralidade da zona central representada por esses dois elementos vem ao encontro da função de validação da zona muda, a qual reforça a transparência das representações tidas nos dois contextos (MENIN, 2006).

Convém notar, é que no núcleo central da zona muda foi possível verificar a presença de elementos também presentes no contexto de normalidade, no entanto, as representações sociais foram distintas entre as duas matrizes, o que pode acontecer conforme revela Abric (2005) ao evidenciar que um mesmo elemento pode ter representações sociais diferentes de acordo com o significado elaborado acerca do objeto.

Nesse sentido, o elemento preconceito, (termo mais evocado pelos profissionais) revelou representações sociais do elemento, no contexto dos serviços de saúde legitimado por sentidos controversos dos profissionais; observou-se ainda, a presença marcante do julgamento baseado em crenças e generalizações ao atribuir referências das pessoas que vivem em sorodiferença para o HIV, a determinados grupos da população como os homossexuais, e pessoas com comportamentos promíscuos.

O desconhecimento e o medo, prováveis elementos centrais com representações particulares na zona muda marcados na memória coletiva dos participantes, apresentaram forte relação com o preconceito (confirmada na análise de similitude).

A análise possibilitou se pensar a respeito dos demais profissionais de saúde, sobre o déficit de conhecimento quanto aos aspectos específicos da infecção do HIV e, de forma mais peculiar, dos aspectos biopsicossociais das relações sorodiferentes, e que por isso, constantemente alimentam (pré) conceitos estabelecidos historicamente e socialmente, fortalecendo o medo para lidar com esses usuários nos serviços.

O cenário global atualmente sinaliza para a ampliação do conhecimento ao que compete às questões preventivas, onde inclui de forma mais recente o conceito de I=I proporcionada à pessoa que vive com HIV em TARV no tempo mínimo de seis meses, necessária a se compreender pelos profissionais de saúde, uma vez que, muitos se limitam ao uso do preservativo como forma de prevenção entre o casal (SILVA, DUARTE, LIMA, 2020; CALABRESE,MAYER, 2020).

A relação do conjunto representacional amor, loucura e preconceito foi visualizada na figura 2 da análise de similitude. Apesar da cristalização da representação social do amor como elemento central também da zona muda, o termo loucura surge invocando sentidos que reforçam a centralidade do preconceito, uma vez que, mesmo enxergando a nobreza de um sentimento que justifique essa união, depara-se com a controvérsia de encararem a sorodiferença para o HIV com uma certa estranheza e uma relação pouco aceitável.

Na literatura, essas prováveis representações do preconceito e medo foram acessadas entre profissionais de saúde, quando questionados sobre o que pensam em relação ao HIV/aids. Tais elementos ocuparam o núcleo central, fortalecendo assim, a hipótese de uma sociedade de modo geral regada por esses dois sentidos, ancorados na ideia de peste e morte que a aids por muito tempo foi marcada (MACHADO *et al*., 2016; SANTOS *et al*., 2019).

Pôde se visualizar nesse estudo, que esses dilemas cercam também os ambientes de saúde, sinalizando necessidades quanto a mudança de sentidos normativos evidenciados pelo preconceito e o desconhecimento.

O incentivo a busca pelo aprimoramento constante dos profissionais de saúde é visto como estratégia fundamental para driblar o desconhecimento e tornar o

profissional preparado para atender essas parcerias em suas necessidades (SOUZA NETO *et al*., 2016).

Além disso, o preconceito associado ao desconhecimento também apresentou relação forte na análise de similitude, bem como na literatura, ao apontar a educação permanente como estratégia para superar o estigma presente nesses cenários quanto a sorodiferença para o HIV (LAGO; MAKSUD; GONÇALVES, 2013), o que fortalece a hipótese dessa representação.

Segundo Abric (2003), ao levantar a hipótese conceitual da zona muda, infere que as representações acessadas em contexto contra-normativo serem explicadas como projeções de representações verdadeiras dos participantes na voz de outro sujeito (MENIN, 2006). O que implica dizer, que as representações sociais centrais na zona muda se ancoram nos elementos preconceito, desconhecimento, medo, amor e loucura.

Na intenção de fortalecer e complementar os prováveis significados acessados no núcleo central, o segundo quadrante da matriz II, revela através dos termos assistência (termo mais evocado), acolhimento e parceria, conteúdos funcionais que relacionam as práticas de saúde no cotidiano como estratégias que precisam ir ao encontro das demandas dessas parcerias sejam nas consultas, na sala de espera do serviço, ou por meio das atividades educativas.

A relação estabelecida na análise de similitude da matriz II, apresenta forte relação dos termos acolhimento e assistência com o núcleo central desconhecimento, e a relação entre parceria e preconceito, que possivelmente denota que o déficit de conhecimento, bem como o preconceito que os profissionais sentem diante dessas parcerias, trazem implicações diretas para as práticas assistenciais de saúde e o acolhimento, visto como algo necessário e indispensável para assegurar o vínculo dos parceiros nos serviços (SANTOS *et al*., 2019).

O terceiro quadrante, apesar de conter elementos por obterem frequência baixa e ordem de evocação alta, interferem com menos força que a das outras zonas representacionais, remete as representações sociais que implicam na prática dos profissionais, (confirmada na análise de similitude) e apresentam representações que se cristalizaram com a matriz I, nas representações das cognições prevenção e risco.

Outra provável representação deste quadrante que reforçou os sentidos do núcleo central, foram os sentidos relacionados a pena por estarem em uma relação com a ideia de doença e morte, e promiscuidade por atribuir a essas pessoas a adoção

de comportamentos como traição, homossexualidade, considerados reprovados para a sociedade, e portanto, uma condição estigmatizante. Ambas reforçam a ideia do preconceito fragilizando a construção do vínculo dessas parcerias com o serviço (SAID; SEIDL, 2015).

Por fim, neste quadrante os profissionais apontaram a necessidade de capacitação profissional, visando a fortalecer e atualizar, o conhecimento específico para a atenção as pessoas que vivem em sorodiferença para o HIV. E, de forma ampliada considerando fatores para além da esfera biológica, e referem também a necessidade de reforço quanto aos preceitos éticos, considerados fundamentais por se tratar da intimidade dos parceiros, configurado como algo já sinalizado pela literatura (REIS *et al*., 2015).

A zona de contraste revelou provavelmente representações que apesar de não ser um consenso significativo entre os participantes, apresentam o menor índice de evocação, revelando, portanto, sua importância e assumindo o caráter heterogêneo das representações juntamente com as periferias (ABRIC, 2003).

Ao ter os elementos coragem, dificuldade e dúvidas, se percebe cognições que caracterizam o termo parceria (segundo quadrante), pelos profissionais acreditarem ser um ato de coragem dessas pessoas se relacionarem, diante das dificuldades existentes, e que por isso, a presença das dúvidas é algo comum na vivência da sorodiferença, representação esta cristalizada nas duas matrizes.

Em relação ao elemento vida normal carrega consigo significados positivos de que os profissionais já estão mais próximos do contexto da sorodiferença, pois não encaram essas relações com estranheza. Entretanto, as cognições com prováveis sentidos negativos como, egoísmo e impossibilidade, representam barreiras ao colocarem preconceitos e estigmas a frente do seu dever profissional em atender e compreender as possibilidades que tornam viável uma relação sorodiferente para o HIV (LAGO; MAKSUD; GONÇALVES, 2013).

A partir da vertente estrutural que toda representação se organiza em torno do núcleo central, e que tem a função de gerar os outros elementos do quadro dando um sentido e valor, e ao mesmo tempo, organiza e estabiliza os elos existentes entre os elementos (ABRIC, 2003). Verifica-se que a representação dos profissionais de saúde na matriz I teve significados centralizados em sentimentos e aspectos inerentes as pessoas que vivem em sorodiferença para o HIV, e na sua periferia abrigou sentidos

que confirmaram e deram sentido aos elementos normativos das representações centrais acessadas.

Sobre a zona muda, Abric (2003) relata, que, ao se encontrarem no contexto em que possam relatar as representações sem estar sob a censura das normas e protocolos dos serviços os colocam, no protagonismo de conteúdos considerados também normativos, reconhecendo por meio de suas representações sociais, as potentes fragilidades advindas do preconceito e da falta de conhecimento que podem estar presentes nos serviços de assistência especializada e também, nos diferentes serviços da rede de atenção à saúde das pessoas que vivem com HIV que realizam o acompanhamento complementar.

Essa inferência pode ser ainda reforçada pela forte relação estabelecida na análise de similitude dos termos preconceito, desconhecimento e assistência, além de comporem os eixos centrais e organizadores dos demais elementos, potencializando, portanto, a Teoria do Núcleo Central no presente contexto.

8 CONSIDERAÇÕES FINAIS

Ao finalizar este estudo, a respeito das representações sociais na vertente estrutural das pessoas que vivem em sorodiferença para o HIV, foi possível uma maior aproximação dessa realidade, que envolve além dos casais sorodiferentes, os profissionais de saúde dos serviços de assistência especializada do Sistema Único de Saúde.

Observou-se ser um contexto no qual, os profissionais cotidianamente se deparam, tendo que enfrentar dificuldades relacionadas muitas vezes, a falta de normas específicas, como também, da pouca divulgação sobre o conhecimento dessa nova variante envolvendo os casais sorodiferentes para o HIV.

Pode-se afirmar, ser um dilema devido a invasão da intimidade sexual das pessoas acometidas, além de outros comportamentos geradores de medo, preconceito (e autopreconceito), rejeição e insegurança, vivenciado de forma conjunta por profissionais e usuários sorodiferentes. Circunstância essa que pode influenciar na adesão e continuidade do tratamento, impondo aos profissionais de saúde, novos desafios que implicam numa necessidade urgente de atendimento mais direcionado e efetivo da população acometida pelo HIV.

Nessa perspectiva, foi possível identificar-se algumas representações acessadas, que mostraram a diversidade de sentidos e significados que permeiam o fenômeno da sorodiferença para o HIV acessados no contexto de normalidade e contra-normativo.

Vê-se que a centralidade dessas representações na normalidade, foi construída com os prováveis elementos parceria, amor e medo que revelaram significados sensíveis as pessoas que vivem a sorodiferença, por enxergá-los como usuários que sentem e possuem demandas para além do contexto biológico que o processo do adoecimento impõe na relação. No entanto, é possível verificar que os profissionais em suas representações na substituição de contexto, produziram mais associações negativas que em situação normal, reveladas pela centralidade dos elementos preconceito, desconhecimento, medo e loucura.

Verificou-se também, que a centralidade da representação é mantida tanto na análise do quadro de quatro casas quanto na análise da árvore de similitude das matrizes.

Acredita-se que esses resultados possam contribuir para entender como os profissionais pensam sobre esse fenômeno, e sinaliza quanto a necessidade de aperfeiçoamento dos profissionais quanto a superação de estigmas e desinformação, nas diferentes instâncias da rede de serviços promotoras da atenção a saúde das pessoas que vivem com HIV, afim de combater o preconceito instituído e desmistificar conceitos ultrapassados.

Como limitações desse estudo, aponta-se a dificuldade do acesso aos profissionais de saúde diante das ações preventivas e protetivas do isolamento e distanciamento social ocasionado pela pandemia da COVID-19. Além do acesso aos serviços, que se mantiveram de portas fechadas para fins acadêmicos, durante o primeiro período previsto para a etapa da coleta de dados no cronograma desta pesquisa.

É importante afirmar, a necessidade de novos estudos no sentido de confirmar a centralidade dos elementos da representação social, e ampliar a população do estudo para além dos profissionais dos serviços especializados, uma vez que entende-se a importância de se verificar os sentidos e significados do fenômeno em questão para cenários que também lidam com essas demandas, como é o caso dos profissionais da Atenção Primária à Saúde.

REFERENCIAS

ABREU, P.D.; *et al*. Representações sociais de mulheres transexuais vivendo com HIV/Aids. **Rev Bras Enferm**, Brasília, v. 73, n. 3, 2020.

ABRIC, J. C. A zona muda das representações sociais. In: OLIVEIRA, D.C.; CAMPOS, P.H.F. **Representações sociais:** Uma teoria sem fronteiras. Rio de Janeiro (RJ): Museu da República, 2005.

ABRIC, J.C. A abordagem estrutural das representações sociais. In: MOREIRA, A.S.P.; OLIVEIRA, D.C. **Estudos interdisciplinares de representação social**. Goiânia (GO): Cultura e Qualidade, 2002. p. 27-38.

ALBUQUERQUE, J.R.; BATISTA, A.T.B.; SALDANHA, A.A.W. O fenômeno do preconceito nos relacionamentos sorodiferentes para o HIV/aids. **Psicologia, saúde & doenças**, v. 19, n. 2, p. 405-421, 2018.

ALMEIDA JUNIOR, J.A.; *et al*. Teste rápido para HIV: representações sociais de profissionais da atenção básica. **Rev Baiana Enferm**, v. 32, 2018.

ALMEIDA, A.M.O. Abordagem societal das representações sociais. **Sociedade e Estado**, v. 24, n. 3, p. 713-737, 2009.

AMORIM, C.M.; SZAPIRO, A.M. Analisando a problemática do risco em casais que vivem em situação de sorodiscordância. **Ciência & Saúde Coletiva**, v. 13, n. 6, p.1859-1868, 2008.

ANGELIM, R.C.M.; *et al*. Representações e práticas de cuidado de profissionais de saúde às pessoas com HIV. **Rev Esc Enferm USP**, São Paulo, v. 53, 2019.

BARBARÁ, A.; *et al*. Contribuições das representações sociais ao estudo da aids. **Interação em Psicologia**, v. 9, n. 2, p. 331-339, 2005.

BOA, M.F.; *et al*. Relacionamentos sorodiscordantes ao HIV/AIDS: representações sociais femininas e práticas de cuidados. **Revista Interamericana de Psicologia**, v. 52, n. 3, p. 370-378, 2018.

BRANDAO, B.M.G.M.; *et al*. Representações sociais de idosos soropositivos acerca do hiv/aids. **Rev Bras Enferm**, Brasília, v. 72, n. 5, p. 1349-1355, 2019.

BRASIL. Ministério da Saúde. **Política Nacional de DST/aids:** princípios e diretrizes. Brasília (DF): Ministério da Saúde, 1999.

BRASIL. Ministério da Saúde. Departamento de Atenção Básica. **HIV/Aids, hepatites e outras DST**. Brasília (DF): Ministério da Saúde, 2006.

BRASIL. Ministério da Saúde. Secretaria de Vigilância em Saúde. Departamento de Vigilância, Prevenção e Controle das Infecções Sexualmente Transmissíveis, do HIV/Aids e das Hepatites Virais. **Cuidado integral às pessoas que vivem com HIV pela Atenção Básica:** manual para a equipe multiprofissional. Brasília (DF): Ministério da Saúde, 2017.

BRASIL. Ministério da Saúde. **Protocolo Clínico e Diretrizes Terapêuticas para Manejo da Infecção pelo HIV em Adultos**. Brasília (DF): Ministério da Saúde, 2018a.

BRASIL. Ministério da Saúde. Secretaria de Vigilância em Saúde. Departamento de Vigilância, Prevenção e Controle das Infecções Sexualmente Transmissíveis, do HIV/Aids e das Hepatites Virais. **Protocolo Clínico e Diretrizes Terapêuticas para Profilaxia Pré-Exposição (PrEP) de Risco à Infecção pelo HIV**. Brasília (DF): Ministério da Saúde, 2018b.

BRASIL. Ministério da Saúde. Secretaria de Vigilância em Saúde. Departamento de Vigilância, Prevenção e Controle das Infecções Sexualmente Transmissíveis, do HIV/Aids e das Hepatites Virais. **Protocolo Clínico e Diretrizes Terapêuticas para Profilaxia Pós-Exposição (PEP) de risco à infecção pelo HIV, IST e Hepatites virais**. Brasília (DF): Ministério da Saúde, 2018c.

BRASIL. Ministério da Saúde. **Boletim Epidemiológico HIV/Aids 2019**. Brasília (DF): Ministério da Saúde, 2019.

BRASIL. Ministério da Saúde. Secretaria de Vigilância em Saúde. Departamento de Vigilância, Prevenção e Controle das Infecções Sexualmente Transmissíveis, do HIV/Aids e das Hepatites Virais. **NOTA INFORMATIVA Nº 5/2019. DIAHV/SVS/MS**. Brasília (DF): Ministério da Saúde, 2019.

BRASIL. Ministério da Saúde. **Boletim Epidemiológico HIV/Aids**. Brasília (DF): Ministério da Saúde, 2020.

CALABRESE, S.K.; MAYER, K.H. Stigma impedes HIV prevention by stifling patient–provider communication about U= U. **Journal of the International AIDS Society**, v. 23, n. 7, 2020.

CAMARGO, B.V.; JUSTO, A.M. IRAMUTEQ: Um Software Gratuito para Análise de Dados Textuais. **Temas em Psicologia**, v. 21, n. 2, p. 513-518, 2013.

CASTRO, J.L.C.; *et al*. Representações sociais do VIH/SIDA para adolescentes: Uma abordagem estrutural. **Aná Psicológica**, v. 37, n. 1, p. 15-27, mar. 2019.

COLACO, A.D.; *et al*. O cuidado à pessoa que vive com HIV/AIDS na atenção primária à saúde. **Texto contexto – enferm,** v. 28, 2019.

COUTINHO, M.F.C.; *et al*. Tratamento antirretroviral: adesão e a influência da depressão em usuários com HIV/Aids atendidos na atenção primária. **Saúde debate,** v. 42, n. 116, 2018.

COUTINHO, M.P.L.; BÚ, E. A técnica de associação livre de palavras sobre o prisma do software tri-deux-mots (version 5.2). **Revista Campo do Saber**, v.3, n.1, 2017.

CRANKSHAW, T.L.; *et al*. Challenges with couples, serodiscordance and HIV disclosure: healthcare provider perspectives on delivering safer conception services for HIV-affected couples. **J Int AIDS Soc**, v. 17, n. 1, 2014.

DANTAS, M.S.; *et al*. Conjugality and a multi-professional health team's social representations of HIV/AIDS. **Revista Enfermagem UERJ,** v. 23, n. 6, 2015.

DOISE, W. **Droits de l'homme et forces des idées**. Paris: PUF, 2001.

DUARTE, L.C.; ROHDEN, F. As histórias que podem ser contadas: a feminização da epidemia HIV/AIDS e a produção de narrativas científicas. **Em construção,** n. 5, p. 22-36, 2019.

FELIX, J.F.B.; *et al*. Análise dos parceiros sorodiferentes no serviço de referência para HIV. **Rev enferm UFPE,** v. 13, 2019.

FERNANDES, N.M.; *et al*. Vulnerabilidade à infecção do HIV entre casais sorodiscordantes no Rio de Janeiro. **Cad Saúde Pública,** v. 33, n. 4, p. 1-9, 2017.

FLAMENT, C.; ROUQUETTE, M.L. **Anatomie des idées ordinaires***.* Paris: Armand Colin, 2003.

FLEISCHER, S. Novas abordagens para casais sorodiferentes. **Horiz Antropol.**, Porto Alegre, v. 19, n. 40, p. 471-474, 2013.

FRANÇA, L. C. M.; *et al*. Spirituality for people living with HIV/AIDS: an analysis of the procedural approach of social representations. **Research, Society and Development**, v. 9, n. 8, 2020.

GOIS, A.R.S.; *et al*. Representações sociais de profissionais do sexo homossexuais, travestis e mulheres transexuais sobre a síndrome da imunodeficiência adquirida. **Enfermería Actual de Costa Rica**, San José, n. 38, p. 121-135, 2020.

GREENER, R.; *et al*. Healthcare providers' understanding of HIV serodiscordance in South Africa and Uganda: implications for HIV prevention in sub-Saharan Africa. **Afr J AIDS Res,** v. 17, n. 2, p. 137-144, 2018.

GUIMARÃES, M.D.C.; *et al*. Mortalidade por HIV/Aids no Brasil, 2000- 2015: motivos para preocupação? **Revista Brasileira de Epidemiologia**, v. 20, p. 182-190, 2017.

GUIRA, O.; *et al*. Sexualité et risque de transmission sexuelle du virus de l'immunodéficience humaine chez les couples sérodiscordants à Ouagadougou (Burkina Faso). **Sexologies**, v. 22, n. 3, p. 138-141, 2013.

GUTIN, A.S.; *et al*. I did not know about all these: Perceptions regarding safer conception methods by women living with HIV in Gaborone, Botswana. **PLoS One**, v. 15, n. 12, 2020.

HALLAL, R.C.; *et al*. Strategies to prevent HIV transmission to serodiscordant couples. **Rev Bras Epidemiol**., v. 18, n. 1, p. 169-82, 2015.

JODELET, D. O Movimento de Retorno ao Sujeito e a Abordagem das Representações Sociais. **Sociedade e Estado**, Brasília, v. 24, n. 3, p. 679-712, 2009.

JUGA, A.J.C.; *et al*. Factors associated with HIV serodiscordance among couples in Mozambique: Comparison of the 2009 INSIDA and 2015 IMASIDA surveys. PLoS ONE, v. 15, n. 6, 2020.

KENYON, C.R.; *et al*. Who Knows Their Partner's HIV Status? Results From a Nationally Representative Survey in Uganda. **Journal of Acquired Immune Deficiency Syndromes**, v. 69, n. 1, p. 92-97, 2015.

KING, R.; *et al*. “Maybe his blood is still strong”: a qualitative study among HIV-sero-discordant couples on ART in rural Uganda. **BMC Public Health,** v. 12, n. 801, 2012.

LAGO, E.L.M.; MAKSUD, I.; GONCALVES, R.S. A "sorodiscordância" para profissionais de saúde: estudo qualitativo da assistência em ambulatório de HIV/AIDS em município do Estado do Rio de Janeiro. **Temas psicol,** v. 21, n. 3, p. 973-988, 2013.

LANGENDORF, T.F.; PADOIN, S.M.M.; SOUZA, I.E.O. Men’s sexual and reproductive health in the situation of serodiscordance. **Rev Bras Enferm**. v. 73, n. 6, 2020.

LUZ, P.M.; MIRANDA, K.C.L.; TEIXEIRA, J.M.C. As condutas realizadas por profissionais de saúde em relação à busca de parceiros sexuais de pacientes soropositivos para o HIV/aids e seus diagnósticos sorológicos. **Ciência & Saúde Coletiva**, v. 15, n. supl. 1, p.1191-1200, 2010.

MACHADO, Y.Y.; *et al*. Representações sociais de profissionais de saúde sobre HIV/AIDS: uma análise estrutural. **Rev enferm UERJ**, v. 24, n. 1, 2016.

MAGNABOSCO, G.T.; *et al*. Assistência ao HIV/aids: análise da integração de ações e serviços de saúde. **Esc Anna Nery**, v. 22, n. 4, 2018.

MAJOR, B.; *et al*. Stigma and its implication for health: introduction and overview. In: MAJOR, B.; DOVIDIO, J.F.; LINK, B.G. **The Oxford handbook of stigma, discrimination, and health**. Nova York: Oxford University Press, 2017. p. 3-28.

MAKSUD, I. O discurso da prevenção da Aids frente às lógicas sexuais de casais sorodiscordantes: sobre normas e práticas. **Physis Revista de Saúde Coletiva**, Rio de Janeiro, v. 19, n. 2, p. 349-369, 2009.

MAKSUD, L. Silêncios e segredos: aspectos (não falados) da conjugalidade face à sorodiscordância para o HIV/AIDS. **Caderno de Saúde Pública**, v. 28, n. 6, p. 1196-1204, 2012.

MARFATIA, Y.S.; *et al*. Profile of HIV seroconcordant/discordant couples a clinic based study at Vadodara, India. **Indian J Sex Transm Dis,** v. 34, p. 5-9, 2013.

MATTHEWS, L.T.; *et al*. Beyond HIV-serodiscordance: Partnership communication dynamics that affect engagement in safer conception care. **PLoS ONE,** v. 12, n. 9, 2017.

MENDES, F.R.P.; *et al*. Social Representations of nursing students about hospital assistance and primary health care. **Rev Bras Enferm**, v. 69, n. 2, p. 321-8, 2016.

MENIN, M.S.S. Representação social e estereótipo: a zona muda das representações sociais. **Psicologia: Teoria e Pesquisa**, v. 22, n. 1, p. 43-52, 2006.

MINAYO, M.C.S. **O desafio do conhecimento:** pesquisa qualitativa em saúde. São Paulo: Hucitec, 2013.

MORTON, J.F.; *et al*. Counseling framework for hiv-serodiscordant couples on the integrated use of antiretroviral therapy and pre-exposure prophylaxis for HIV Prevention. **Journal of Acquired Immune Deficiency Syndromes,** v. 74, n. 1, p. 15-22, 2017.

MOSCOVICI, S. **Representações Sociais**: Investigações em Psicologia Social. 5ª ed. Petrópolis (RJ): Vozes, 2007.

NGURE, K.; *et al*. "I never thought that it would happen … " Experiences of HIV seroconverters among HIV-discordant partnerships in a prospective HIV prevention study in Kenya. **AIDS Care**, v. 5, p. 1-4, jun. 2016.

ODOYO, J.B.; *et al*. Integrating Pr EP into HIV care clinics could improve partner testing services and reinforce mutual support among couples: provider views from a Pr EP implementation project in Kenya. **Journal of the International AIDS Society**, v. 22, p. e25303, 2019.

OJIKUTU, B.O.; *et al*. Community Cultural Norms, Stigma and Disclosure to Sexual Partners among Women Living with HIV in Thailand, Brazil and Zambia (HPTN 063). **PLoS One,** v. 11, n. 5, 2016.

OLIVEIRA, D.C.; COSTA, T.L. A zona muda das representações sociais sobre o portador de HIV/AIDS: elementos normativos e contranormativos do pensamento social. **Psicologia: Teoria e Prática**, v. 9, n. 2, p. 73-91, 2007.

OLIVEIRA, J.A.A.; *et al*. Estratégias ao casal em situação de sorodiscordância para o HIV: uma revisão da literatura. **Revista JRG de Estudos Acadêmicos**, v. 3, n. 7, 2020.

OLIVEIRA, L.B.; *et al*. Asociación sexual entre personas que viven con el VIH: manejo de las diferencias serológicas. **Enfermeria global**, v. 1, n. 58, p. 494-506, 2020.

PATEL, R.C.; *et al*. What motivates serodiscordant couples to prevent HIV transmission within their relationships: findings from a PrEP implementation study in Kenya. **Cult Health Sex.**, v. 20, n. 6, p. 625-39, 2018.

REIS, K. R.; GIR, E. Convivendo com a diferença: o impacto da sorodiscordância na vida afetivo-sexual de portadores do HIV/AIDS. **Rev Esc Enfermagem USP**, v. 44, n. 3, p. 759-65, 2010.

REIS, K.R.; GIR, E. Vulnerabilidade ao HIV/AIDS e a prevenção da transmissão sexual entre casais sorodiscordantes. **Rev Esc Enfermagem USP**, v. 43, n. 3, p. 662-69, 2009.

REIS, R.K.; *et al*. Inconsistent condom use between serodifferent sexual partnerships to the human immunodeficiency virus. **Rev Latino-Am Enfermagem**, v. 29, 2019.

REIS, R.K.; GIR, E. Dificuldades enfrentadas pelos parceiros sorodiscordantes ao HIV na manutençao do sexo seguro. **Rev Latino-Am Enfermagem**, v. 13, n. 1, p. 32-37, 2005.

REIS, R.K.; NEVES, L.A.S.; GIR, E. Desejo de ter filhos e planejamento familiar entre casais sorodiscordantes ao HIV**. Cienc Cuid Saude,** v. 12, n. 2, p. 210-218, 2013.

SA, A.A.M; SANTOS, C.V.M. A Vivência da Sexualidade de Pessoas que Vivem com HIV/Aids. **Psicol Cienc Prof.**, Brasília, v. 38, n. 4, p. 773-786, 2018.

SÁ, C.P.; ARRUDA, A. **O estudo das representações sociais no Brasil**. Florianópolis: EDUFSC, 2000.

SAHANA, S.; BETKERUR, J. Profile of HIV serodiscordant couples in a tertiary care center. **Indian J Dermatol Venereol Leprol,** v. 85, 2019.

SAID, A.P.; SEIDL, E.M.F. Serodiscordance and prevention of HIV: perceptions of individuals in stable and non-stable relationships. **Interface,** v. 19, n. 54, p. 467-78, 2015.

SANTOS, F.S.; *et al*. Acolhimento à pessoa com o vírus da imunodeficiência humana: representações sociais de profissionais de saúde. **Rev Baiana Enferm**., v. 33, 2019.

SILVA, A.M.; CAMARGO JUNIOR, K.R. A invisibilidade da sorodiscordância na atenção às pessoas com HIV/AIDS. **Ciênc Saúde Coletiva**, v. 16, n. 12, p. 4865-4874, 2011.

SILVA, F.M.V.; *et al*. O ser-com-o-outro na condição sorodiscordante: uma abordagem fenomenológica da vulnerabilidade individual ao HIV. **Rev Eletr Enf**, v. 20, 2018.

SILVA, F.M.V.; GUEDES, T.G. Vulnerabilidade individual ao HIV/Aids nas relações sorodiscordantes. **Enfermagem Brasil** v. 16, n. 6, 2017.

SILVA, L.A.V.; DUARTE, F.M.; LIMA, M. Modelo matemático pra uma coisa que não é matemática: narrativas de médicos/as infectologistas sobre carga viral indetectável e intransmissibilidade do HIV. **Physis: Revista de Saúde Coletiva**, Rio de Janeiro, v. 30, n. 1, e300105, 2020.

SILVA, V.G.F.; *et al*. The Influence of Serodiscordance on the Behavior and Motivation of Sexual Practices among People Living with HIV/AIDS: a Qualitative Study. **J Nurs Health Sci,** v. 4, n. 3, 2018.

SILVEIRA, R.C.W.; LAZZAROTTO, A.R.A. Qualidade de vida de pessoas vivendo com hiv/aids: estudo transversal com as organizações não governamentais. **SEFIC,** 2018.

SOUZA NETO, V.L.; *et al*. Sorodiscordância na atenção às pessoas com HIV/AIDS: implicações para o enfermeiro. **Rev Fund Care Online**, v. 8, n. 4, p. 5184-519, 2016.

SOUZA, M.A.R.; *et al*. O uso do software IRAMUTEQ na análise de dados em pesquisas qualitativas. **Rev Esc Enferm USP**, São Paulo, v. 52, 2018.

SPINK, M.J. **O conhecimento no cotidiano:** as representações sociais na perspectiva da psicologia social. São Paulo: Brasiliense, 1995.

SPINK, M.J.P. The Concept of Social Representations in Social Psychology. **Cad Saúde Públ**, Rio de Janeiro, v. 9, n. 3, p. 300-308, 1993.

STAKE, R. E. **Pesquisa qualitativa:** estudando como as coisas funcionam. Porto Alegre: Penso, 2011.

SUTO, C.S.S.; *et al*. Profissionais de saúde falam mais sobre cuidado e menos sobre síndrome da imunodeficiência adquirida. **Cogitare Enferm**, v. 22, n. 3, 2017.

TAYLOR, T.N. "The Pleasure Is Better as I've Gotten Older": Sexual Health, Sexuality, and Sexual Risk Behaviors Among Older Women Living With HIV. **Arch Sex Behav,** v. 46, n. 4, p. 1137-1150, 2017.

TEIXEIRA, M.G.; *et al*. Conquistas do SUS no enfrentamento das doenças transmissíveis. **Ciênc. saúde coletiva**, Rio de Janeiro, v. 23, n. 6, p. 1819-28, 2018.

TELLALIAN, D.; *et al*. Pre-Exposure Prophylaxis (PrEP) for HIV Infection: Results of a Survey of HIV Healthcare Providers Evaluating Their Knowledge, Attitudes, and Prescribing Practices**. AIDS Patient Care and STDs,** v. 27, n. 10, 2013.

TRIANI, F.S.; BIZERRA C.C.; NOVIKOFF, C. A influência da cultura sobre as representações sociais. **Revista Educação e Cultura Contemporânea,** v. 14, n. 36, 2017.

TRIGUEIRO, D.R.S.G.; *et al*. AIDS and jail: social representations of women in freedom deprivation situations. **Rev Esc Enferm USP**, São Paulo, v. 50, n. 4, p. 554-61, 2016.

UNAIDS. Programa Conjunto das Nações Unidas sobre HIV/Aids. **Estatística 2020**. Brasília (DF): UNAIDS, 2020.

WACHELKE, J.; WOLTER, R. Critérios de construção e relato da análise prototípica para representações sociais. **Psic Teor Pesq.**, Brasília, v. 27, n. 4, p. 521-26, 2011.

WARE, N.C.; *et al*. O que o amor tem a ver com isso? Explicando a adesão à profilaxia antirretroviral pré-exposição oral para casais sorodiscordantes de HIV. **Journal of Acquired Immune Deficiency Syndromes,** v. 59, n. 5, p. 463-468, 2012.

WEST, N.; *et al*. I don't know if this is right…but this is what I'm offering: healthcare provider knowledge, practice, and attitudes towards safer conception for HIV-affected couples in the context of Southern African guidelines. **AIDS Care**, v. 28, n. 3, p. 390-396, 2016.

WHO. World Health Organization. **Guidance on pre-exposure oral prophylaxis (PrEP) for serodiscordant couples, men and transgender women who have sex with men at high risk of HIV:** recommendations for use in the context of demonstration projects. Genebra: WHO, 2012.

ZAKABI, D. Testagem de HIV na atenção primária: limites e potencialidades em Fortaleza, CE, Brasil. **Essentia**, v. 18, n. 1, p.72-83, 2017.

APÊNDICES

APÊNDICE A - INSTRUMENTO PARA COLETA DE DADOS

UNIVERSIDADE FEDERAL DO RIO GRANDE DO NORTE
CENTRO DE CIÊNCIAS DA SAÚDE
DEPARTAMENTO DE ENFERMAGEM
PROGRAMA DE PÓS-GRADUAÇÃO EM ENFERMAGEM
MESTRADO EM ENFERMAGEM
Campus Universitário Sn – Lagoa Nova, Natal/RN CEP. 59072-970, Fone: 3215-3196

Ficha: ______ Data da coleta: ____/____/_______ Local: __________

Horário de início: _________ Horário do término: ____________

ENTREVISTA SEMIESTRUTURADA

**CARACTERIZAÇÃO DOS PARTICIPANTES**	
Sexo: F ( ) M ( )	Idade:
Nível de escolaridade: Ensino Superior ( ) Ensino Técnico ( )	
Profissão no serviço:	
Tempo de formação na área:	
Tempo de serviço no SAE IST/HIV/Aids:	
Possui especialização ou capacitação para atuar no SAE IST/HIV/Aids: Sim ( ) Não ( )<br>Se sim, especifique. ____	

UNIVERSIDADE FEDERAL DO RIO GRANDE DO NORTE
CENTRO DE CIÊNCIAS DA SAÚDE
DEPARTAMENTO DE ENFERMAGEM
PROGRAMA DE PÓS-GRADUAÇÃO EM ENFERMAGEM
MESTRADO EM ENFERMAGEM
Campus Universitário Sn – Lagoa Nova, Natal/RN CEP. 59072-970, Fone: 3215-3196

TESTE DE ASSOCIAÇÃO LIVRE DE PALAVRAS

TERMO INDUTOR: Se eu lhe digo **"PESSOAS EM SORODIFERENÇA PARA O HIV"**, quais as 5 primeiras palavras ou expressões que lhe vem à mente?

1.____

2.____

3.____

4.____

5.____

Diante das cinco palavras acima mencionadas, defina ou justifique o que cada uma delas representa para você.

1.____
____
____
____

2.____
____
____
____

3.____
____
____
____

4.____
____
____
____

5.____
____
____
____

UNIVERSIDADE FEDERAL DO RIO GRANDE DO NORTE
CENTRO DE CIÊNCIAS DA SAÚDE
DEPARTAMENTO DE ENFERMAGEM
PROGRAMA DE PÓS-GRADUAÇÃO EM ENFERMAGEM
MESTRADO EM ENFERMAGEM
Campus Universitário Sn – Lagoa Nova, Natal/RN CEP. 59072-970, Fone: 3215-3196

ZONA MUDA

Se eu lhe digo **"PESSOAS EM SORODIFERENÇA PARA O HIV"**, o que o você acha que os profissionais de saúde pensam?

1.____

2.____

3.____

4.____

5.____

Diante das cinco palavras acima mencionadas, defina ou justifique o que cada uma delas representam para os profissionais de saúde.

1.____
____
____
____

2.____
____
____
____

3.____
____
____
____

4.____
____
____
____

5.____
____
____
____

APÊNDICE B - TERMO DE CONSENTIMENTO LIVRE E ESCLARECIDO – TCLE

Esclarecimentos

Este convite é para você participar da pesquisa: **Representações Sociais das pessoas que vivem em sorodiferença para o HIV elaboradas por profissionais de saúde**. Tem como pesquisadora responsável, a Profa. Dra. Rejane Maria Paiva de Menezes e, como pesquisadora colaboradora, a Mestranda Valéria Gomes Fernandes da Silva, ambas do Programa de Pós-Graduação em Enfermagem da Universidade Federal do Rio Grande do Norte.

Seu objetivo é analisar como os profissionais dos Serviços de Assistência Especializada IST/HIV/Aids, pensam sobre a sorodiferença nesse âmbito de atendimento em saúde. O motivo que nos levou a realizá-lo, se reveste da importância do papel dos profissionais de saúde, no atendimento das pessoas em sorodiferença para o HIV, inseridas no cenário do cuidado e enfrentamento do HIV, em seu cotidiano profissional.

Caso decida participar, o(a) senhor(a) passará por uma entrevista onde responderá alguns questionamentos que estarão divididos em duas partes: a primeira, contendo informações de caracterização pessoal como idade, sexo, nível de escolaridade, e também profissional, como profissão, tempo de atuação no serviço, etc. Na segunda parte, o senhor (a) responderá algumas perguntas abertas, sobre o que pensa a respeito de pessoas em sorodiferença para o HIV. O tempo previsto para responder a 1ª parte é calculado em 5 minutos (total de 7 perguntas), e a 2ª parte podendo variar na média de 25 a 45 minutos (total de 4 perguntas), totalizando o tempo máximo de 50 minutos.

Ressalta-se, que a coleta dos dados será realizada em ambiente adequado e privado, de forma que as suas respostas estarão sob sigilo, e tanto sua privacidade quanto anonimato, serão garantidos durante todo o transcorrer do processo.

Durante a realização da pesquisa poderão ocorrer eventuais desconfortos e possíveis riscos como timidez ou inibição em responder algum dos questionamentos. Esses riscos poderão ser minimizados através da garantia do sigilo absoluto das informações recebidas e a garantia de que seu nome não será exposto. Para isso, a ficha com as informações correspondentes à sua participação, será identificada pela letra “P” seguida da letra que representa sua profissão (a exemplo, PE (Profissional Enfermeiro).

Como benefícios, espera-se através desse estudo divulgar os resultados entre os profissionais de saúde proporcionando-lhes acesso as informações obtidas relacionadas à sorodiferença, e assim contribuir para maior visibilidade diante dos serviços de saúde e do âmbito social.

Em caso de algum problema que você possa ter relacionado com a pesquisa, você terá direito à assistência gratuita que será prestada pelos pesquisadores responsáveis.

Durante todo o período da pesquisa você poderá tirar suas dúvidas ligando para a mestranda Valéria Gomes Fernandes da Silva, por meio do contato (84) 99927-2586 ou do e-mail valeriafernandes7@hotmail.com, e/ou da Profa. Rejane Maria Paiva de Menezes no contato fixo 3215-3615 (84) ou celular 991581245, no e-mail rejemene@gmail.com, e ambas no endereço: Campus Universitário – UFRN, Lago Nova, Departamento de Enfermagem.

Você tem o direito de se recusar a participar ou retirar seu consentimento, em qualquer fase da pesquisa, sem nenhum prejuízo para você.

As informações dadas por você em nenhum momento serão identificadas com seu nome, garantindo seu anonimato, e divulgadas apenas nos congressos científicos ou publicações científicas.

Os resultados desse estudo serão guardados pelo pesquisador responsável por essa pesquisa em local seguro e por um período de 5 anos.

Os gastos financeiros da sua participação na pesquisa, é de responsabilidade do pesquisador e, caso aconteça, será devolvido para você. Se você sofrer qualquer dano decorrente desta pesquisa, sendo ele imediato ou tardio, previsto ou não, você será indenizado.

Qualquer dúvida sobre a ética dessa pesquisa você deverá ligar para o Comitê de Ética em Pesquisa – instituição que avalia a ética das pesquisas antes que elas comecem e fornece proteção aos participantes das mesmas – da Universidade Federal do Rio Grande do Norte, nos telefones (84) 3215-3135 / (84) 9.9193.6266, através do e-mail cepufrn@reitoria.ufrn.br Você ainda pode ir pessoalmente à sede do CEP, de segunda a sexta, das 08:00h às 12:00h e das 14:00h às 18:00h, na Universidade Federal do Rio Grande do Norte, Av. Senador Salgado Filho, s/n. Campus Central, Lagoa Nova. Natal/RN.

Este documento foi impresso em duas cópias. Uma ficará com você e a outra com a pesquisadora colaboradora Valéria Gomes Fernandes da Silva.

Consentimento Livre e Esclarecido

Após ter sido explicado sobre os objetivos, importância e o modo como as informações serão coletadas nessa pesquisa, além de conhecer os riscos e benefícios que ela trará para mim, e ter entendido sobre todos os meus direitos, aceito participar da pesquisa **Representações Sociais das pessoas em sorodiferença para o HIV elaboradas por profissionais de saúde**, e autorizo a divulgação das informações por mim fornecidas em congressos e/ou publicações científicas desde que nenhum dado possa me identificar.

Natal (RN), / / .

____

Assinatura do participante da pesquisa

Impressão datiloscópica do participante

Declaração do pesquisador responsável

Como pesquisadora responsável pelo estudo: **Representações Sociais das pessoas em sorodiferença para HIV elaboradas por profissionais de saúde**, declaro que assumo a inteira responsabilidade de cumprir fielmente os procedimentos metodológicos e direitos que foram esclarecidos e assegurados ao participante desse estudo, assim como manter sigilo e confidencialidade sobre a identidade do mesmo.

Declaro ainda estar ciente que na inobservância do compromisso ora assumido estarei infringindo as normas e diretrizes propostas pela Resolução 466/12 do Conselho Nacional de Saúde – CNS, que regulamenta as pesquisas envolvendo o ser humano.

Natal (RN), / / .

____

Assinatura do pesquisador responsável

APÊNDICE C - NOTA PRÉVIA DO PROJETO DE PESQUISA

ISSN: 1676-4285

OBJN Online Brazilian Journal of Nursing
PORTUGUÊS
Universidade Federal Fluminense
ESCOLA DE ENFERMAGEM AURORA DE AFONSO COSTA
uff
Editorial

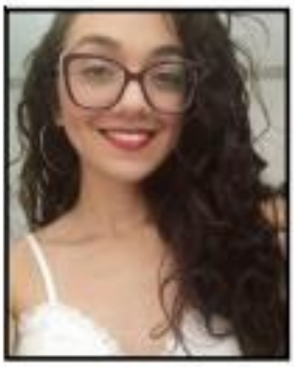

Representações sociais da sorodiferença ao HIV/aids por profissionais de saúde: estudo descritivo

Valéria Gomes Fernandes da Silva[1], Carlos Jordão de Assis Silva[1], Alexandra do Nascimento Cassiano[1], Bruna Ruselly Dantas Silveira[1], Eloisa Araújo de Carvalho[1], Rejane Maria Paiva de Menezes[1]

1 Universidade Federal do Rio Grande do Norte

RESUMO

Objetivo: analisar as representações sociais de pessoas em sorodiferença ao HIV elaboradas por profissionais de saúde. **Método:** estudo descritivo, ancorado na vertente estrutural das Representações Sociais. O local de realização do estudo serão três Unidades de Assistência Especializada às pessoas que vivem com HIV da região metropolitana de Natal-RN. Os participantes, profissionais da equipe multiprofissional de saúde, responsáveis pela assistência aos indivíduos que vivem em sorodiferença ao HIV. A coleta de dados ocorrerá através de entrevista semiestruturada, guiada por questões abertas sobre o fenômeno em questão e, análise dos resultados, sob a luz da Teoria das Representações Sociais e da literatura pertinente. **Resultados Esperados:** espera-se que os resultados alcançados, deem aproximação e visibilidade ao fenômeno e contribuam para o atendimento das necessidades de cuidado biopsicossocial, inerente ao processo saúde-doença da sorodiferença, no campo multiprofissional, sobretudo dos cuidados de enfermagem, com vistas a promoção da saúde dos sujeitos em questão.
Descritores: Aids; HIV; Promoção da Saúde; Serviços de Saúde.

Silva VGF, Silva CJA, Cassiano AN, Silveira BRD, Carvalho EA, Menezes RMP. Representações sociais da sorodiferença ao HIV/aids por profissionais de saúde: estudo descritivo. Online Braz J Nurs [Internet]. 2020 xx [cited xx xx xx];19(Suppl):xx-xx. Available from: https://doi.org/10.17665/1676-4285.20206427

INTRODUÇÃO

Os Serviços de Assistência Especializada (SAE) responsáveis por assistir sujeitos que vivem com o Vírus da Imunodeficiência Humana (HIV) têm acolhido usuários envolvidos afetivamente e sexualmente com parceiros que possuem sorologia diferente da sua, configurando, portanto, a relação de sorodiferença[1].

No cotidiano de pessoas que vivem em sorodiferença, é possível visualizar desafios a serem superados, como o receio em transmitir o vírus ao parceiro soronegativo, dificuldades de adaptação às mudanças de comportamento e medo da não aceitação da condição sorológica[2].

Essa problemática se aflora, quando vem acompanhada de um desequilíbrio psicológico/emocional, por compreender que a sexualidade não direciona seu conceito apenas ao ato sexual, mas abrange características humanas que tem a natureza de se ligar às pessoas, ao prazer, aos desejos, às necessidades, à vida[1]. Esses sujeitos compartilham cotidianamente tais situações com os profissionais que os assistem, envolvendo-os de forma direta aos seus anseios e expectativas[2].

Diante do contexto de dúvidas, vulnerabilidades e medo, os profissionais de saúde desempenham um papel decisivo e substancial na construção de vínculos, informações, cuidados e acolhimento aos parceiros. São eles os responsáveis por disseminar o conhecimento e favorecer a desconstrução de preconceitos enraizados que envolvem as relações sorodiferentes[2].

Destaca-se a necessidade de identificar os aspectos que envolvem o contexto da sorodiferença, no qual, o cuidado implica num olhar em sua dimensão mais ampla, perpassando o seguimento afetivo, emocional, familiar e social, que tem como princípio a integralidade e não apenas a execução de procedimentos para promover o conforto ou atender à necessidade dos pacientes[3].

Nesse sentido, percebe-se a necessidade de analisar o significado atribuído a pessoas em sorodiferença ao HIV entre os profissionais de saúde responsáveis pelo acompanhamento terapêutico desses sujeitos. Portanto, diante da importância dessa temática, considera-se pertinente responder à seguinte questão norteadora: Quais as representações sociais das pessoas em sorodiferença ao HIV, elaboradas por profissionais dos Serviços de Assistência Especializada HIV/aids?

Espera-se então que a questão posta, ao ser analisada pelos profissionais de saúde, possa alcançar além da identificação, também a percepção e/ou conhecimento construídos, sobre as representações sociais que perpassam nas relações que envolvem os casais sorodiferentes. E, a partir dos resultados, contribuir com o redirecionamento das práticas de saúde e solidificação de políticas de enfrentamento[3].

OBJETIVO

Analisar as representações sociais das pessoas em sorodiferença para o HIV, elaboradas pelos profissionais dos Serviços de Assistência Especializada em HIV/aids.

MÉTODO

Estudo descritivo exploratório, com abordagem qualitativa, o qual será desenvolvido em três unidades de Serviços de Assistência Especializada, localizados na região metropolitana do estado do Rio Grande do Norte.

A coleta de dados ocorrerá através de entrevista semiestruturada onde a população prevista a participar do estudo consta de um quantitativo de 52 profissionais de saúde de equipes multiprofissionais distribuídos nesses serviços. O arrolamento dos participantes obedecerá aos seguintes critérios de inclusão:ser profissional da equipe de saúde multiprofissional básica dos SAEs, composta por médico (infectologistas e/ou clínico geral), assistentes sociais, psicólogos, farmacêuticos, enfermeiros, técnicos de enfermagem, profissionais médicos ginecologistas da equipe especializada e profissionais responsáveis pela gestão e coordenação local do SAE e do programa DST/HIV/aids dos municípios citados e do estado.

O projeto em consideração foi submetido à Plataforma Brasil e conforme parecer do Comitê de Ética e Pesquisa (CEP) da Universidade Federal do Rio Grande do Norte, em conformidade com a resolução 466-/12, foi considerado aprovado, sob o parecer 4.005.590.

A coleta de dados será iniciada imediatamente após a autorização dos serviços de saúde na qualidade de cenários de pesquisas, tendo suas atividades de pesquisas paralisadas em virtude das consequências geradas pela pandemia do novo coronavírus. A análise dos dados será realizada em conformidade com a Teoria das Representações Sociais, sob a perspectiva da vertente estrutural, proposta por Jean Claude Abric, com o auxílio do software *Interface de R pour les Analyses Multidimensionelles de Textes et de Questionaires (IRAMUTEQ)*.

RESULTADOS ESPERADOS

O presente estudo tem contribuição direta com a geração de debates ao evidenciar a temática da sorodiferença sob o olhar do profissional de saúde. Pretende-se tornar as representações sociais obtidas, objeto estruturante na desconstrução de conceitos ultrapassados e estigmas prevalentes nos cenários sociais, incluindo os ambientes de saúde. Assim, espera-se que os resultados alcançados gerem uma aproximação e uma visibilidade efetiva quanto à necessidade do cuidado biopsicossocial inerente ao processo saúde-doença envolvendo a sorodiferença, redirecionando as práticas de saúde e favorecendo o processo reflexivo dos profissionais de saúde próximos ou distantes dessa temática.

REFERÊNCIAS

1. Fernandes NM, Hennington EA, Bernardes JS, Grinsztejn BG. Vulnerabilidade à infecção do HIV entre casais sorodiscordantes no Rio de Janeiro, Brasil. Cad Saúde Pública [internet]. 2017 [Cited 2020 Jul 22];33(4):e00053415. Available from: https://www.scielo.br/scielo.php?pid=S0102311X2017000405014&script=sci_abstract&tlng=pt. doi: https://doi.org/10.1590/0102-311X00053415.

2. Souza Neto VL, Silva BCO, Rodrigues IDCV, Costa CS, Mendonça AEO, Negreiros RV. Serodiscordance in care for people with HIV/AIDS: implications for nurses. Rev Fund Care Online [internet]. 2016 [Cited 2020 Jul 22];8(4):5184-92. Available from: http://www.seer.unirio.br/index.php/cuidadofundamental/article/view/3808/pdf_1. doi: http://dx.doi. org/10.9789/2175-5361.2016.v8i4.5184-5192.

3. Santos FS, Suto CSS, Freitas TOB, Piva SGN, Nascimento RCD, Souza GS. User-embracement for the person with the human immunodeficiency virus: social representations of health professionals. Rev baiana enferm [Internet]. 2019 [Cited 2020 Jul 22];33:e27769. Available from: https://portalseer.ufba.br/index.php/enfermagem/article/view/27769/19114. doi: http://dx.doi.org/10.18471/rbe.v33.27769.

Recebido: 03/09/2020
Revisado: 03/09/2020
Aprovado: 08/09/2020

Contribuição dos autores

Valéria Gomes Fernandes da Silva foi responsável pela elaboração do projeto de pesquisa. Rejane Maria Paiva de Menezes foi orientadora do projeto de pesquisa. Análise e aprovação final da versão a ser publicada: todos os autores.

ANEXOS

ANEXO A - CARTA DE ANUÊNCIA DA SECRETARIA MUNICIPAL DE SAÚDE DE NATAL

NATAL

SECRETARIA MUNICIPAL DE SAÚDE
Departamento de Gestão do Trabalho e Educação na Saúde – DGTES
Setor de Educação Permanente e Articulação Ensino Serviço - SEPAES

TERMO DE ANUÊNCIA

Nº 06

Declaro por meio deste que esta Secretaria Municipal de Saúde está de acordo com a pesquisa intitulada **"Representações Sociais Das Pessoas Em Sorodiferença HIV/Aids Elaboradas Por Profissonais De Saúde"**. Esta pesquisa será realizada pela discente Valeria Gomes Fernandes Da Silva , do Programa de Pós - Graduação em Enfermagem, da Universidade Federal do Rio Grande do Norte - UFRN, a coleta será realizada no Serviço De Assistência Especializada IST/HIV/ Aids do Municipio de Natal- RN, a ser realizada entre os meses de junho e agosto de 2020.

Consideramos que este projeto poderá contribuir para a produção de conhecimento acerca deste tema, em virtude disto autorizamos a presença do(a) discente nas dependências do Serviço De Assistência Especializada IST/HIV/ Aids do Municipio de Natal/RN bem como a abordagem dos profissionais da Rede Municipal de Saúde do Natal.

A autorização desta pesquisa está condicionada ao cumprimento das normas e diretrizes propostas pela Resolução 466/12 do Conselho Nacional de Saúde – CNS, que regulamenta as pesquisas envolvendo o ser humano.

A divulgação dos resultados obtidos em fóruns, revistas/Jornais científicos ficará autorizada, desde que seja mantido o sigilo sobre a identificação das unidades/usuários.

Solicitamos ainda que o resultado da pesquisa seja consolidado e entregue ao setor ao setor de educação Permanente e articulação Ensino – Serviço para o banco de dimensionamento de pesquisas da Secretaria Municipal de Saúde.

Natal, 09 de Março de 2020.

George de Antunes de Oliveira
Secretário Municipal de Saúde

ANEXO B - CARTA DE ANUÊNCIA DA SECRETARIA MUNICIPAL DE SAÚDE DE PARNAMIRIM

Estado do Rio Grande do Norte
Prefeitura Municipal de Parnamirim
Secretaria Municipal de Saúde

CARTA DE ANUÊNCIA

Por ter sido informado verbalmente e por escrito sobre os objetivos e metodologia da pesquisa intitulada Representações Sociais das pessoas em sorodiferença para o HIV elaboradas por profissionais de saúde, coordenada pelo (a) Prof (a) Rejane Maria Paiva de Menezes, concordo em autorizar a realização da (s) etapa (s) processo de coleta de dados nesta Instituição que represento.

Esta Instituição está ciente de suas corresponsabilidades como instituição coparticipante do presente projeto de pesquisa, e de seu compromisso no resguardo da segurança e bem-estar dos participantes de pesquisa nela recrutados, dispondo de infraestrutura necessária para a garantia de tal segurança e bem-estar.

Esta autorização está condicionada à aprovação prévia da pesquisa acima citada por um Comitê de Ética em Pesquisa e ao cumprimento das determinações éticas propostas na Resolução 466/12 do Conselho Nacional de Saúde – CNS e suas complementares.

O descumprimento desses condicionamentos assegura-me o direito de retirar minha anuência a qualquer momento da pesquisa.

Natal/RN 02 de abril de 2020.

Terezinha Guedes Rêgo
Secretária Municipal de Saúde

Terezinha G. Rêgo de Oliveira
Secretária Municipal de Saúde/SESAD
Parnamirim/RN

ANEXO C - CARTA DE ANUÊNCIA DA SECRETARIA DE ESTADO DE SAÚDE PÚBLICA DO RIO GRANDE DO NORTE

Governo do Estado do RN
Secretaria de Estado da Saúde Pública

Hospital Giselda Trigueiro
Direção Geral

CARTA DE ANUÊNCIA

Por ter sido informado verbalmente e por escrito sobre os objetivos e metodologia da pesquisa intitulada "**Representações Sociais das Pessoas em Sorodiferença para o HIV Elaboradas por Profissionais de Saúde**", referente à dissertação de mestrado em Enfermagem, sob orientação da Professora Dra. Rejane Maria de Paiva Menezes, autorizo a realização da pesquisa com os profissionais de saúde da equipe do Serviço de Assistência Especializada-SAE do Hospital Giselda Trigueiro.

Esta autorização está condicionada à aprovação prévia da pesquisa acima citada por um Comitê de Ética em Pesquisa e ao cumprimento das determinações éticas propostas na Resolução 466/12 do Conselho Nacional de Saúde – CNS.

O descumprimento desses condicionamentos assegura-me o direito de retirar minha anuência a qualquer momento da pesquisa.

Natal/RN, 09 de março de 2020.

André Luciano de Araújo Prudente
Diretor Geral

Rua Cônego Monte, 110 – Quintas – CEP 59037-170 - Natal/RN
Fone: (84) 3232-7915 - FAX (84) 3232-7965 - E-mail: [illegible] e [illegible]

ANEXO D - PARECER CONSUBSTANCIADO DO COMITÊ DE ÉTICA EM PESQUISA

UFRN - UNIVERSIDADE FEDERAL DO RIO GRANDE DO NORTE - LAGOA NOVA CAMPUS CENTRAL

Plataforma Brasil

PARECER CONSUBSTANCIADO DO CEP

DADOS DO PROJETO DE PESQUISA

Título da Pesquisa: Representações sociais das pessoas em sorodiferença para o HIV elaboradas por profissionais de saúde

Pesquisador: REJANE MARIA PAIVA DE MENEZES

Área Temática:

Versão: 1

CAAE: 30794020.6.0000.5537

Instituição Proponente: Pós-Graduação em Enfermagem

Patrocinador Principal: Financiamento Próprio

DADOS DO PARECER

Número do Parecer: 4.005.590

Apresentação do Projeto:

Projeto de Mestrado do Departamento de Enfermagem – Programa de Pós Graduação em Enfermagem.

Nº participantes da pesquisa: 52 (participação dos profissionais de saúde que compõem a equipe multiprofissional básica composta por médicos, assistentes sociais, psicólogos, farmacêuticos e equipe de enfermagem (enfermeiros e técnicos de enfermagem), bem como profissionais médicos ginecologistas da equipe especializada e profissionais responsáveis pela gestão e coordenação local dos SAEs e do programa IST/HIV/Aids dos municípios citados e do estado).

No contexto de assistência à saúde dos serviços especializados, constantemente os profissionais se deparam com a realidade das pessoas que convivem com a sorodiferença para o HIV. Percebe-se haver uma simbologia construída acerca da sorodiferença por meio do conhecimento e das experiências vivenciadas pelos profissionais, que torna evidente esse fenômeno no âmbito da saúde. Este estudo tem o objetivo de analisar as representações sociais sob a vertente estrutural das pessoas em sorodiferença para o HIV, elaboradas pelos profissionais de saúde do Serviço de Assistência Especializada em IST/HIV/Aids. Trata-se de um estudo descritivo, exploratório, com abordagem qualitativa, ancorado nos pressupostos da Teoria das Representações Socais na vertente estrutural, a ser realizado com os profissionais de saúde de três Serviços de Assistência Especializada em Infecções Sexualmente Transmissíveis HIV/Aids, localizados nos municípios de

Endereço: Av. Senador Salgado Filho, 3000
Bairro: Lagoa Nova **CEP:** 59.078-970
UF: RN **Município:** NATAL
Telefone: (84)3215-3135 **Fax:** (84)99193-6266 **E-mail:** cepufrn@reitoria.ufrn.br

Natal e Parnamirim, no estado do Rio Grande do Norte. Os participantes são os profissionais que compõem a equipe multiprofissional mínima de saúde, coordenadores e gestores locais desses serviços de assistência. Como critérios de exclusão, àqueles profissionais ausentes do serviço em período de férias e licença médica, e os profissionais que não atenderem a três tentativas de contato. Para a coleta de dados, utilizar-se-á a técnica de associação livre de palavras e de substituição e descontextualização (Zona Muda), a fim de revelar conteúdos não encontrados nos discursos normatizados. O tratamento e organização dos dados será feito, por meio do processamento prototípico no qual, calculam-se as frequências e ordens médias de evocações das palavras das entrevistas, cujos elementos serão base para elaboração do quadro de quatro casas (Teoria do Núcleo Central), distribuídos conforme análise entre núcleo central e a periferia da representação a partir dos elementos de natureza normativa e funcional, e da análise de similitude. E, para a análise propriamente dita, o uso do software Interface de Rapouries Analyses Multidimensionnelles de Textes et de Questionnaires (IRaMuTeQ), versão 7 alpha 2. O projeto será submetido ao Comitê de Ética e Pesquisa da Universidade Federal do Rio Grande do Norte de acordo com os preceitos éticos estabelecidos pela Resolução Nº 466, de 12 de Dezembro de 2012 do Conselho Nacional de Saúde. Dos resultados obtidos, espera-se alcançar o entendimento dos profissionais dos Serviços de Assistência Especializada sobre as pessoas em sorodiferença para o HIV e de que forma esse entendimento se estrutura. Assim como, contribuir para o direcionamento da prática em saúde de outros profissionais envolvidos com o fenômeno, nesse cenário de atenção à saúde.

> Critério de Inclusão:

Ser profissional de saúde da equipe de saúde multiprofissional básica dos SAEs, composta por médico (infectologistas ou clínico geral), assistentes sociais, psicólogos, farmacêuticos e equipe de enfermagem (enfermeiros e técnicos de enfermagem), profissionais médicos ginecologistas da equipe especializada e profissionais responsáveis pela gestão e coordenação local do SAE e do programa DST/HIV/Aids dos municípios citados e do estado.

Critério de Exclusão:

Não participarão do estudo os profissionais ausentes do serviço em período de férias e licença médica, bem como os profissionais que não atenderem as três tentativas de contato.

Objetivo da Pesquisa:

Objetivo Primário:

Analisar as representações sociais na vertente estrutural das pessoas em sorodiferença para o HIV, elaboradas pelos profissionais dos Serviços de Assistência Especializada em IST/HIV/Aids.

Objetivo Secundário:

a) Identificar a estrutura das representações sociais das pessoas em sorodiferença para o HIV, elaboradas pelos profissionais do SAE IST/HIV/Aids;

b) Identificar a zona muda das representações sociais das pessoas em sorodiferença para o HIV, elaboradas pelos profissionais do SAE IST/HIV/Aids;

c) Compreender a estrutura e os significados representacionais da sorodiferença para profissionais do SAE IST/HIV/Aids.

Avaliação dos Riscos e Benefícios:
Riscos:

De acordo com a pesquisadora, este projeto oferece risco mínimo de desconforto e constrangimento durante a aplicação do instrumento de coleta de dados. Para minimizá-los, as entrevistas serão realizadas individualmente, em ambiente tranquilo, reservado e privado de movimento e interferências externa.

Benefícios:

O estudo de representações sociais tem contribuição direta com a geração de debates em torno de um fenômeno, e a escolha em tornar evidente a temática da sorodiferença sob o olhar do profissional de saúde, consiste na tentativa de tornar as representações sociais obtidas, objeto estruturante na contribuição do processo de desmistificação de preconceitos e estigmas enraizados na sociedade e nos ambientes de saúde como um todo acerca dessa temática. Assim, a participação em um estudo que possibilite essa reflexão instigará os profissionais participantes a pensarem em seu conhecimento construído bem como sua relação com as práticas de saúde, favorecendo o processo reflexivo necessário para si e para os demais profissionais de saúde

próximos ou distantes dessa temática.

Comentários e Considerações sobre a Pesquisa:

A pesquisa trará pensamentos reflexivos dos profissionais de saúde com o intuito de desmistificar preconceitos e estigmas sobre o tem AIDS em pacientes soropositivos.

Considerações sobre os Termos de apresentação obrigatória:

O projeto apresentou os seguintes documentos obrigatórios, de acordo com a resolução 466/12- CNS.

- PB_Projeto de pesquisa
- Formulário CEP
- Projeto de Pesquisa na integra
- Justificativa de anuências em PDF
- Termo de confidencialidade
- Folha de Rosto
- Anuência HGT e Secretaria Municipal de Saúde
- TCLE
- Instrumentos de coleta
- Declaração de não inicio

Recomendações:

Conforme procedimentos definidos na Resolução CNS 466/12 do Conselho Nacional de Saúde (CNS) do Ministério da Saúde (MS), cabe ao pesquisador elaborar e apresentar os relatórios parciais, final ou de suspensão para avaliação do CEP. Caro(a) pesquisador(a), se, em decorrência da pandemia do Corona vírus (COVID-19) o cronograma apresentado sofrer alteração, favor encaminhar novo cronograma ao CEP Central/UFRN, sob a forma de notificação do tipo "Comunicação de Início do Projeto".

Conclusões ou Pendências e Lista de Inadequações:

Após a análise dos documentos e projeto de pesquisa, a luz da Resolução 466/12-CNS, não forma identificados óbices éticos. A pesquisa está aprovada.

É de responsabilidade do CEP Central/UFRN alertar que, mesmo o projeto não apresentando óbices éticos e estando, dessa forma, aprovado, o desenvolvimento de etapas com participantes só deverá ocorrer após o fim do distanciamento social e da redução dos riscos relacionados ao Corona vírus (COVID-19), a fim de priorizar a saúde da comunidade, mediante a redução da propagação do vírus e da disseminação da doença. Caso o(a) coordenador(a) da pesquisa decida executar as etapas com participantes de pesquisa antes do fim do distanciamento social, deverá assumir inteira responsabilidade por tal decisão e pelas consequências dessa ação.

Considerações Finais a critério do CEP:
Em conformidade com a Resolução 466/12 e ou a Resolução 510/16 do Conselho Nacional de Saúde - CNS e Manual Operacional para Comitês de Ética - CONEP é da responsabilidade do pesquisador responsável:

1. Elaborar o Termo de Consentimento Livre e Esclarecido - TCLE em duas vias, rubricadas em todas as suas páginas e assinadas, ao seu término, pelo convidado a participar da pesquisa, ou por seu representante legal, assim como pelo pesquisador responsável, ou pela (s) pessoa (s) por ele delegada(s), devendo as páginas de assinatura estar na mesma folha (Res. 466/12 - CNS, item IV.5d);
2. Desenvolver o projeto conforme o delineado (Res. 466/12 - CNS, item XI.2c);
3. Apresentar ao CEP eventuais emendas ou extensões com justificativa (Manual Operacional para Comitês de Ética - CONEP, Brasília - 2007, p. 41);
4. Descontinuar o estudo somente após análise e manifestação, por parte do Sistema CEP/CONEP/CNS/MS que o aprovou, das razões dessa descontinuidade, a não ser em casos de justificada urgência em benefício de seus participantes (Res. 446/12 - CNS, item III.2u);
5. Elaborar e apresentar os relatórios parciais e finais (Res. 446/12 - CNS, item XI.2d);
6. Manter os dados da pesquisa em arquivo, físico ou digital, sob sua guarda e responsabilidade, por um período de 5 anos após o término da pesquisa (Res. 446/12 - CNS, item XI.2f);
7. Encaminhar os resultados da pesquisa para publicação, com os devidos créditos aos pesquisadores associados e ao pessoal técnico integrante do projeto (Res. 446/12 - CNS, item XI.2g) e,
8. Justificar fundamentadamente, perante o CEP ou a CONEP, interrupção do projeto ou não

publicação dos resultados (Res. 446/12 - CNS, item XI.2h).

Este parecer foi elaborado baseado nos documentos abaixo relacionados:

Tipo Documento	Arquivo	Postagem	Autor	Situação
Informações Básicas do Projeto	PB_INFORMAÇÕES_BÁSICAS_DO_PROJETO_1536256.pdf	10/04/2020 19:46:15		Aceito
Outros	FORMULARIOCEPUFRN.docx	10/04/2020 19:45:09	REJANE MARIA PAIVA DE	Aceito
Outros	FORMULARIOCEPUFRN.pdf	10/04/2020 19:44:15	REJANE MARIA PAIVA DE	Aceito
Projeto Detalhado / Brochura Investigador	PROJETODEPESQUISA.pdf	10/04/2020 19:26:24	REJANE MARIA PAIVA DE MENEZES	Aceito
Projeto Detalhado / Brochura Investigador	PROJETODEPESQUISA.docx	10/04/2020 19:25:54	REJANE MARIA PAIVA DE MENEZES	Aceito
Outros	JUSTIFICATIVA.docx	10/04/2020 19:10:18	REJANE MARIA PAIVA DE	Aceito
Outros	JUSTIFICATIVAA.pdf	10/04/2020 19:07:38	REJANE MARIA PAIVA DE	Aceito
Outros	TERMODECONFIDENCIALIDADE.docx	06/04/2020 20:18:31	REJANE MARIA PAIVA DE	Aceito
Outros	TERMODECONFIDENCIALIDADEE.pdf	06/04/2020 20:17:50	REJANE MARIA PAIVA DE	Aceito
Outros	INSTRUMENTOCOLETADEDADOS.docx	06/04/2020 20:10:05	REJANE MARIA PAIVA DE	Aceito
Outros	INSTRUMENTOCOLETADEDADOS.pdf	06/04/2020 20:09:22	REJANE MARIA PAIVA DE	Aceito
Declaração de Pesquisadores	DECLARACAONAOINICIO.docx	06/04/2020 20:07:19	REJANE MARIA PAIVA DE	Aceito
Declaração de Pesquisadores	DECLARACAONAOINICIO.pdf	06/04/2020 20:06:17	REJANE MARIA PAIVA DE	Aceito
Declaração de Instituição e Infraestrutura	CARTADEANUENCIA2.pdf	06/04/2020 20:04:09	REJANE MARIA PAIVA DE MENEZES	Aceito
Declaração de Instituição e Infraestrutura	CARTADEANUENCIA1.pdf	06/04/2020 20:03:28	REJANE MARIA PAIVA DE MENEZES	Aceito
TCLE / Termos de Assentimento / Justificativa de Ausência	TCLE.docx	06/04/2020 19:51:14	REJANE MARIA PAIVA DE MENEZES	Aceito
TCLE / Termos de	TCLE.pdf	06/04/2020	REJANE MARIA	Aceito

Endereço: Av. Senador Salgado Filho, 3000
Bairro: Lagoa Nova **CEP:** 59.078-970
UF: RN **Município:** NATAL
Telefone: (84)3215-3135 **Fax:** (84)99193-6266 **E-mail:** cepufrn@reitoria.ufrn.br

Assentimento / Justificativa de Ausência	TCLE.pdf	19:47:11	PAIVA DE MENEZES	Aceito
Folha de Rosto	FOLHADEROSTO.pdf	06/04/2020 19:41:21	REJANE MARIA PAIVA DE	Aceito

Situação do Parecer:
Aprovado

Necessita Apreciação da CONEP:
Não

NATAL, 05 de Maio de 2020

Assinado por:
LÉLIA MARIA GUEDES QUEIROZ
(Coordenador(a))

Endereço: Av. Senador Salgado Filho, 3000
Bairro: Lagoa Nova **CEP:** 59.078-970
UF: RN **Município:** NATAL
Telefone: (84)3215-3135 **Fax:** (84)99193-6266 **E-mail:** cepufrn@reitoria.ufrn.br
