# Supplementary material for: HIV serodiscordant sexual partners: social representations of health care professionals
Source: Rev Bras Enferm. 2022 Jun 24;75(6):e20210867. doi: 10.1590/0034-7167-2021-0867 (PMC9728823; doi:10.1590/0034-7167-2021-0867)
Supplement: 0034-7167-reben-75-06-e20210867-sup02 [file 0034-7167-reben-75-06-e20210867-sup02.pdf]

ISSN: 1676-4285

OBJN Online Brazilian Journal of Nursing ENGLISH

Federal Fluminense University

AURORA DE AFONSO COSTA NURSING SCHOOL

uff Summary of theses and dissertations

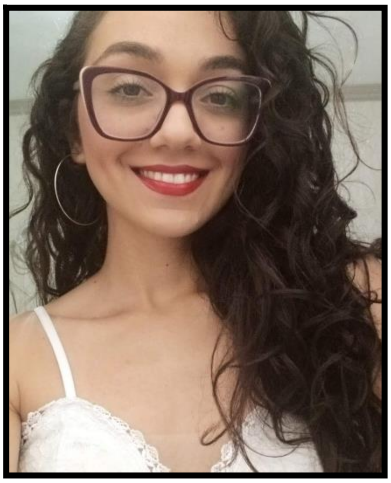

Social representations of serodiscordance to HIV/AIDS by health professionals: a descriptive study

Valéria Gomes Fernandes da Silva[1], Carlos Jordão de Assis Silva[1], Alexandra do Nascimento Cassiano[1], Bruna Ruselly Dantas Silveira[1], Eloísa Araújo de Carvalho[1], Rejane Maria Paiva de Menezes[1]

1 Federal University of Rio Grande do Norte

ABSTRACT

Objective: to analyze the social representations of people in serodiscordance to HIV elaborated by health professionals. **Method:** a descriptive study, based on the structural strand of the Social Representations. The study locus will be three Specialized Assistance Units for people living with HIV in the metropolitan region of Natal-RN. The participants will be professionals of the multi-professional health team, responsible for assisting individuals living with serodiscordance to HIV. Data collection will take place through semi-structured interviews, guided by open questions about the phenomenon in question and analysis of the results, in the light of the Theory of Social Representations and the pertinent literature. **Expected results:** it is expected that the results achieved provide approximation and visibility to the phenomenon and contribute to meeting the biopsychosocial care needs, inherent to the health-disease process of serodiscordance, in the multi-professional field, especially of Nursing care, aimed at the promotion of the health of the subjects in question.

Descriptors: AIDS; HIV; Health promotion; Health services.

Silva VGF, Silva CJA, Cassiano AN, Silveira BRD, Carvalho EA, Menezes RMP. Representações sociais da sorodiferença ao HIV/aids por profissionais de saúde: estudo descritivo. Online Braz J Nurs [Internet]. 2020 xx [cited xx xx xx];19(Suppl):xx-xx. Available from: https://doi.org/10.17665/1676-4285.20206427

INTRODUCTION

The Specialized Assistance Services (SAS) responsible for assisting people living with the Human Immunodeficiency Virus (HIV) has been welcoming users affectively and sexually involved with a partner who has a different serology from theirs, therefore configuring the relation of serodiscordance[1].

In the daily life of the people who live in serodiscordance, it is possible to view challenges to be overcome, as fearing to transmit the virus to the seronegative partner, difficulties in adapting to behavior changes, and fear of not accepting the serologic condition[2].

This problem arises when it is accompanied by a psychological/emotional imbalance because it is understood that the concept of sexuality is not only directed to the sexual act but encompasses human characteristics that have the nature of connecting to people, pleasure, desires, needs, and life[1]. These subjects share such situations on a daily basis with the professionals who assist them, involving them in a direct manner into their wishes and expectations[2].

Faced with the context of doubts, vulnerabilities and fear, the health professionals play a decisive and substantial role in building bonds, information, care actions and welcoming of the partners. They are responsible for spreading knowledge and promoting the deconstruction of deeply-rooted prejudices that involve serodiscordant relationships[2].

The need is highlighted to identify the aspects that involve the context of serodiscordance, in which care requires a look at its wider dimension, passing through the affective, emotional, family, and social follow-up, which has integrality as its principle and not only the performance of procedures to promote comfort or meet the needs of the patients[3].

In this sense, the need is perceived to analyze the meaning assigned to individuals in serodiscordance to HIV among the health professionals responsible for the therapeutic follow-up of these subjects. Therefore, given the importance of this theme, it is considered relevant to answer the following guiding question: What are the social representations of people in serodiscordance to HIV, prepared by the professionals of Specialized HIV/AIDS Assistance Services?

It is then expected that this given question, when analyzed by the health professionals, may achieve not only the identification, but also the perception and/or recognition built about the social representations that permeate the relation involving serodiscordant couples. And, from the results, contribute to the redirection of the health practices and solidification of confrontation policies[3].

OBJECTIVE

To analyze the social representations of people in serodiscordance to HIV, elaborated by the professionals of the Specialized HIV/AIDS Assistance Services.

METHOD

This is a descriptive and exploratory study with a qualitative approach, which will be developed in three units of the Specialized Assistance Service, located in the metropolitan region of the state of Rio Grande do Norte.

Data collection will take place by means of semi-structured interviews, where the population expected to participate in the study totals 52 health professionals and multi-professional teams distributed in these services. The enrollment of the participants will obey the following inclusion criteria: being a professional in the basic multi-professional health team of the SAS, made up of physicians (infectologists and/or general practitioner), social workers, psychologists, pharmacists, nurses, nursing technicians, gynecologists of the specialized team, and professionals responsible for the local management and coordination of the SAS and of the STD/HIV/AIDS program in the cities mentioned and the state.

The project under consideration was submitted to *Plataforma Brasil* and, according to the opinion of the Ethics and Research Committee (*Comitê de Ética e Pesquisa*, CEP) of the Federal University of Rio Grande do Norte, in accordance with Resolution 466/12, was considered approved, under opinion number 4,005,590.

Data collection will begin immediately after authorization of the health services as places for the research scenario, with its research activities being paralyzed because of the consequences generated by the novel coronavirus pandemic. Data analysis will be carried out in accordance with the Theory of Social Representations, from the perspective of the structural strand, proposed by Jean Claude Abric, with the aid of the *Interface de R pour les Analyses Multidimensionelles de Textes et de Questionaires (IRAMUTEQ)* software.

EXPECTED RESULTS

This study has a direct contribution to the generation of debates by highlighting the issue of serodiscordance from the perspective of the health professional. It is intended to turn the social representations obtained into a structuring object in the deconstruction of outdated concepts and stigma prevalent in the social settings, including health environments. Thus, it is expected that the results achieved provide approximation and effective visibility regarding the need for biopsychosocial care inherent to the health-disease process involving serodiscordance, redirecting the health practices, and favoring the reflexive process of the health professionals close to or distant from this theme.

REFERENCES

1. Fernandes NM, Hennington EA, Bernardes JS, Grinsztejn BG. Vulnerabilidade à infecção do HIV entre casais sorodiscordantes no Rio de Janeiro, Brasil. Cad Saúde Pública [internet]. 2017 [Cited 2020 Jul 22];33(4):e00053415. Available from: https://www.scielo.br/scielo.php?pid=S0102311X2017000405014&script=sci_abstract&tlng=pt. doi: https://doi.org/10.1590/0102-311X00053415.

2. Souza Neto VL, Silva BCO, Rodrigues IDCV, Costa CS, Mendonça AEO, Negreiros RV. Serodiscordance in care for people with HIV/AIDS: implications for nurses. Rev Fund Care Online [internet]. 2016 [Cited 2020 Jul 22];8(4):5184-92. Available from: http://www.seer.unirio.br/index.php/cuidadofundamental/article/view/3808/pdf_1. doi: http://dx.doi. org/10.9789/2175-5361.2016.v8i4.5184-5192.

3. Santos FS, Suto CSS, Freitas TOB, Piva SGN, Nascimento RCD, Souza GS. User-embracement for the person with the human immunodeficiency virus: social representations of health professionals. Rev baiana enferm [Internet]. 2019 [Cited 2020 Jul 22];33:e27769. Available from: https://portalseer.ufba.br/index.php/enfermagem/article/view/27769/19114.doi: http://dx.doi.org/10.18471/rbe.v33.27769.

Received: 09/03/2020
Revised: 09/03/2020
Approved: 09/08/2020

Contribution of the authors

Valéria Gomes Fernandes da Silva was responsible for elaborating the research project. Rejane Maria Paiva de Menezes guided the research project. Analysis and final approval of the version to be published: all the authors.
